# Cu-Catalyzed Arylation of Bromo-Difluoro-Acetamides by Aryl Boronic Acids, Aryl Trialkoxysilanes and Dimethyl-Aryl-Sulfonium Salts: New Entries to Aromatic Amides

**DOI:** 10.3390/molecules26102957

**Published:** 2021-05-16

**Authors:** Satenik Mkrtchyan, Michał Jakubczyk, Suneel Lanka, Michael Pittelkow, Viktor O. Iaroshenko

**Affiliations:** 1 Laboratory of Homogeneous Catalysis and Molecular Design at the Center of Molecular and Macromolecular Studies, Polish Academy of Sciences, Sienkiewicza 112, 90-363 Łodź, Poland; drmjakubczyk@gmail.com (M.J.); suneellanka89@gmail.com (S.L.); 2 Institute of Bioorganic Chemistry, Polish Academy of Sciences, Noskowskiego 12/14, 61-704 Poznań, Poland; 3 Institute of General and Ecological Chemistry, Lodz University of Technology, Zeromskiego 116, 90-924 Lodz, Poland; 4 Department of Chemistry, University of Copenhagen, Universitetsparken 5, 2100 Copenhagen, Denmark; pittel@chem.ku.dk; 5 Dipartimento di Chimica e Biologia “A. Zambelli” Università di Salerno, Via Giovanni Paolo II, 84084 Fisciano (SA), Italy; 6 Department of Chemistry, University of Helsinki, A.I. Virtasen aukio 1, 00014 Helsinki, Finland

**Keywords:** fluorine, amides, copper, catalysis, C-C-coupling, boronic acids, aryl trialkoxysilanes, dimethyl-aryl-sulfonium salts

## Abstract

We describe a mechanism-guided discovery of a synthetic methodology that enables the preparation of aromatic amides from 2-bromo-2,2-difluoroacetamides utilizing a copper-catalyzed direct arylation. Readily available and structurally simple aryl precursors such as aryl boronic acids, aryl trialkoxysilanes and dimethyl-aryl-sulfonium salts were used as the source for the aryl substituents. The scope of the reactions was tested, and the reactions were insensitive to the electronic nature of the aryl groups, as both electron-rich and electron-deficient aryls were successfully introduced. A wide range of 2-bromo-2,2-difluoroacetamides as either aliphatic or aromatic secondary or tertiary amides were also reactive under the developed conditions. The described synthetic protocols displayed excellent efficiency and were successfully utilized for the expeditious preparation of diverse aromatic amides in good-to-excellent yields. The reactions were scaled up to gram quantities.

## 1. Introduction

The amide functional group is abundant in peptides and numerous natural products and is also ubiquitous in a vast range of biologically active compounds, marketed drugs, and a broad spectrum of agrochemicals [1,2,3,4,5,6,7]. The presence of the amide motif or its isosteres condition biological activity of many privileged scaffolds [7]. By recent estimates, almost a quarter of all marketed pharmaceuticals possesses an amide bond, making this functional group the most encountered in medicinal chemistry. Amides are prevalent in advanced materials [7,8], and many life science relevant substances; amides also play pivotal roles in supramolecular chemistry [9,10], molecular recognition [9,10,11], and catalysis [12,13]. The amide functional group can be tuned electronically and conformationally to gain desired structural, physical, and biological properties. The chemistry of amide group is vast, and by its virtue amides can be transformed into many other functional groups [14,15,16,17,18,19,20]. Due to the omnipresence and profound importance of the amide functionality, the development of principally new synthetic routes aiming at installation of the amide structural moiety is of current importance in both modern organic and medicinal chemistry. In this context, many new synthetic routes were elaborated [21,22,23,24]. Among those, it is important to mention such game-changing strategy as aminocarbonylations of aryl halides utilizing CO [25,26,27].

One conceptually underexplored strategy to prepare new amides was the installation of the amide structural unit by the attaching an appropriate substituent onto the prefunctionalized CO-N structural motif bearing a tuned leaving group on the amide carbon. Analysis of the literature revealed that this tactic has been realized using C-N synthons bearing Cl [28] and CHal_3_ (Hal = Cl, Br, I) as a leaving group [29,30,31]. These strategies were predominately used for the construction of aromatic amides with different substituents on the nitrogen atom. Another method was developed that is based upon the transition-metal-catalyzed arylation of N-substituted formamides by different aryl-containing reagents, predominantly aryl halides [32,33,34].

**Scheme 1:**
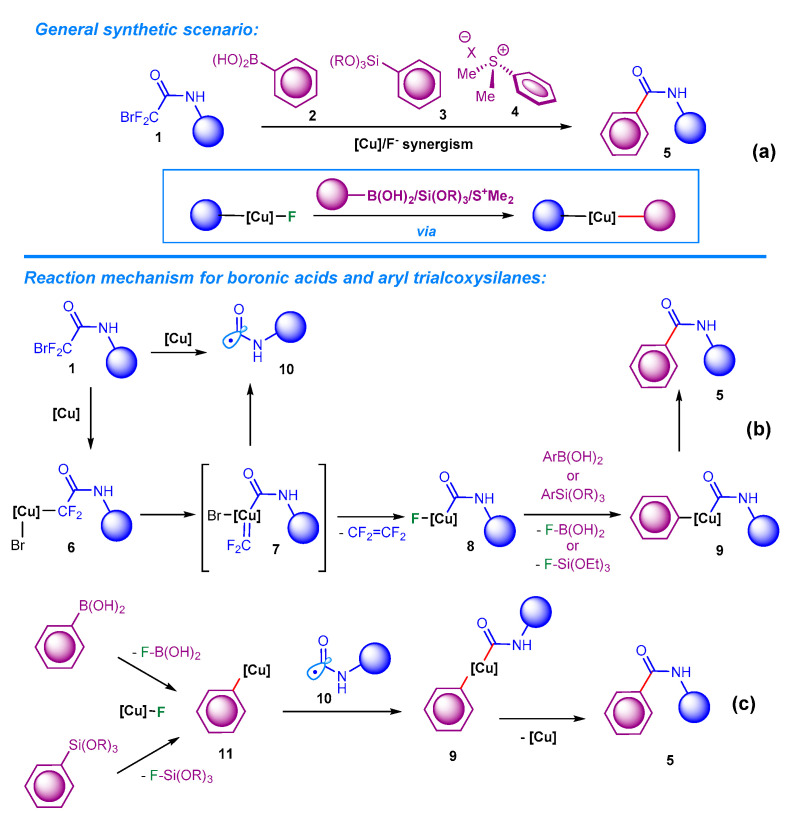
(**a**) Synthetic scenario, (**b**,**c**) Proposed reaction mechanisms.

Based on a mechanistic consideration, we considered that 2-bromo-2,2-difluoroacetamides **1** would be particularly attractive for the formation of aryl-amides by activation using transition-metal catalysis. Combining the halogens in this particular fashion on the trihaloacetamide enables us to harness the attractive features of copper catalysis and fluoride-mediated catalysis. We set out to explore 2-bromo-2,2-difluoroacetamides **1** in coupling reactions with aryl boronic acids **2** and (aryl)trialkoxysilanes **3** arylation agents as donors of aryl or heteroaryl substituents (Scheme 1a). We hypothesized (Scheme 1b) that using transition-metal-assisted catalysis, a 2-bromo-2,2-difluoroacetamide unit could undergo an oxidative addition on an appropriately tuned by ligands metal nuclei, forming an organometallic intermediate (structure **6**) [35,36,37], followed by a rearrangement possibly via a CF_2_-carbene complex **7**, which undergoes loss of difluorocarben and simultaneous exchange of Br versus F giving rise to an organometallic (intermediate **8**) capable of undergoing reaction with aryl boronic acids or aryl trialkoxysilanes to deliver a new intermediate (**9**), which after the reductive elimination would result in the formation of a new C-C bond to yield the desired aryl amide (**5**). An alternative mechanistic pathway could be via copper-intermediate **11** (Scheme 1c), as a result of the reaction between a fluorinated transition-metal catalyst and an aryl boronic acid (or aryl trialkoxysilane). This species could react with a carbon-centered radical **10** to form the species **9,** which then decomposes into the final amide product **5**. The formation of the radical species **10** would be unusual from the mechanistic point of view. A similar mechanism has, however, been suggested on the instance of palladium-catalyzed carboxylate-assisted ethoxycarboxylation of aromatic acids by ethyl bromodifluoroacetate in a very recent study [38,39]. It is worth noting that the concept of F versus B(OR)_2_ (or Si(OR)_3_) exchange on the copper nuclei, which we are postulating here, was suggested by Giri and Brawn for the mechanism in their copper-catalyzed Suzuki–Miyaura C-C couplings. These protocols were operational not only for boronic esters, but also for a broad range of trialkoxysilanes [40,41,42]. Based on the assumption of a fluoride-bearing Ar-Cu-F intermediate being active (similar to structure **8**), and in a view of the resent literature on copper-supported C-C coupling protocols, we envisioned the use of copper catalysts. We also envisioned the preparation of aromatic amides as a result of the C-C coupling between aryl boronic acids, aryl trialkoxysilanes, or sulphonium salts with 2-bromo-2,2-difluoroacetamides according to the general synthetic scenario depicted in the Scheme 1. 

We first considered the use of 2-bromo-2,2-difluoroacetamides as a source of the -CO-NR_2_ synthon. The only literature example known to date where ethoxycarboxylation of aromatic acids occurs using ethyl bromodifluoroacetate was described recently by Zhao et al. [38]. Similar access was proposed by Shi and co-workers in an alkoxycarbonylation of benzamides utilizing chloroformates [28]. Trifluoroacetyl amides have been used for the construction of aromatic and aliphatic amides via C(O)-CF_3_ bond cleavage utilizing the reaction with Grignard reagents [43]. The routes proposed by us utilize commercially or readily available reagents aryl donors and are visibly more atom economic and efficient than those using metalorganic reagents, thus enabling the creation larger amide structural diversities. 

## 2. Results and Discussion 

**Scheme 2:**
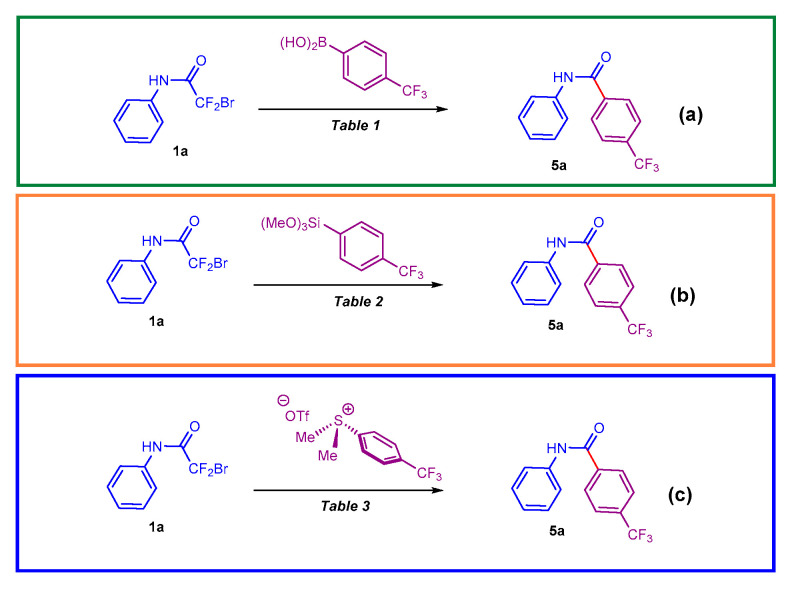
Model reactions for reaction conditions optimization: (**a**) Reaction of 2-bromo-2,2-difluoro-*N*-phenylacetamide with (4-(trifluoromethyl)phenyl)boronic acid, (**b**) Reaction of 2-bromo-2,2-difluoro-*N*-phenylacetamide with trimethoxy(4-(trifluoromethyl)phenyl)silane, (**c**) Reaction of 2-bromo-2,2-difluoro-*N*-phenylacetamide with dimethyl(4-(trifluoromethyl)phenyl)sulfonium triflate.

We selected three model reactions and performed a set of trial experiments to identify the trends and generalities depicted in Scheme 2 and Table 1, Table 2 and Table 3. After testing numerous reaction parameters, among which are catalysts, ligands, solvents, and bases, we noticed that some of the copper salts in combination with nitrogen-containing ligands (not indicated in the optimization Tables), in particular solvents, facilitate the expected C-C-coupling reaction and thus the formation of the desired aromatic amide. Furthermore, we succeeded in establishing the optimal reaction conditions for synthetic protocols (a) and (b), which were identical and consisted in the use of CuBr_2_ (0.1 equiv.), KF (2 equiv.), MgCl_2_ (1 equiv.) with hexafluoropropanol as the solvent, where all reactions were conducted in ACE pressure tubes at 70 °C for 8 h. One crucial aspect appeared to be the addition of calix[4]arene derivatives, which most probably act as ligands for the coper salt. The best efficiency was observed for the corresponding calix[4]arene **L1**. The magnesium salt, due to the high affinity of Mg^2+^ towards electron rich fluoride ion (hardness of Mg^2+^ in terms of the Pearson Hard-Soft acid-base theory), is most probably involved in the activation of one of the C-Hal bonds, like the corresponding C-F bond, by the coordination onto fluorine (where the fluoride ion in turn is a hard base, as per the Pearson Hard-Soft acid-base theory) and formation of the Mg-haloalkane complex [44,45]. The optimized reaction conditions allowed the efficient preparation of the model amide compound **5a** in 87% and 90%, respectively (Table 1 and Table 2). This success encouraged further exploration of the scope and limitation of these two new protocols. We set out to test the scope and limitations of these coupling reactions by selecting twenty-two 2-bromo-2,2-difluoroacetamides **1** and reacting those with a range of aryl boronic acids **2** (twenty-three different substrates) and aryl trialkoxysilanes **3** (seventeen substrates). In a result of this study, we successfully prepared thirty-one amide derivativities **5** in good-to-excellent yields.

**Scheme 3:**
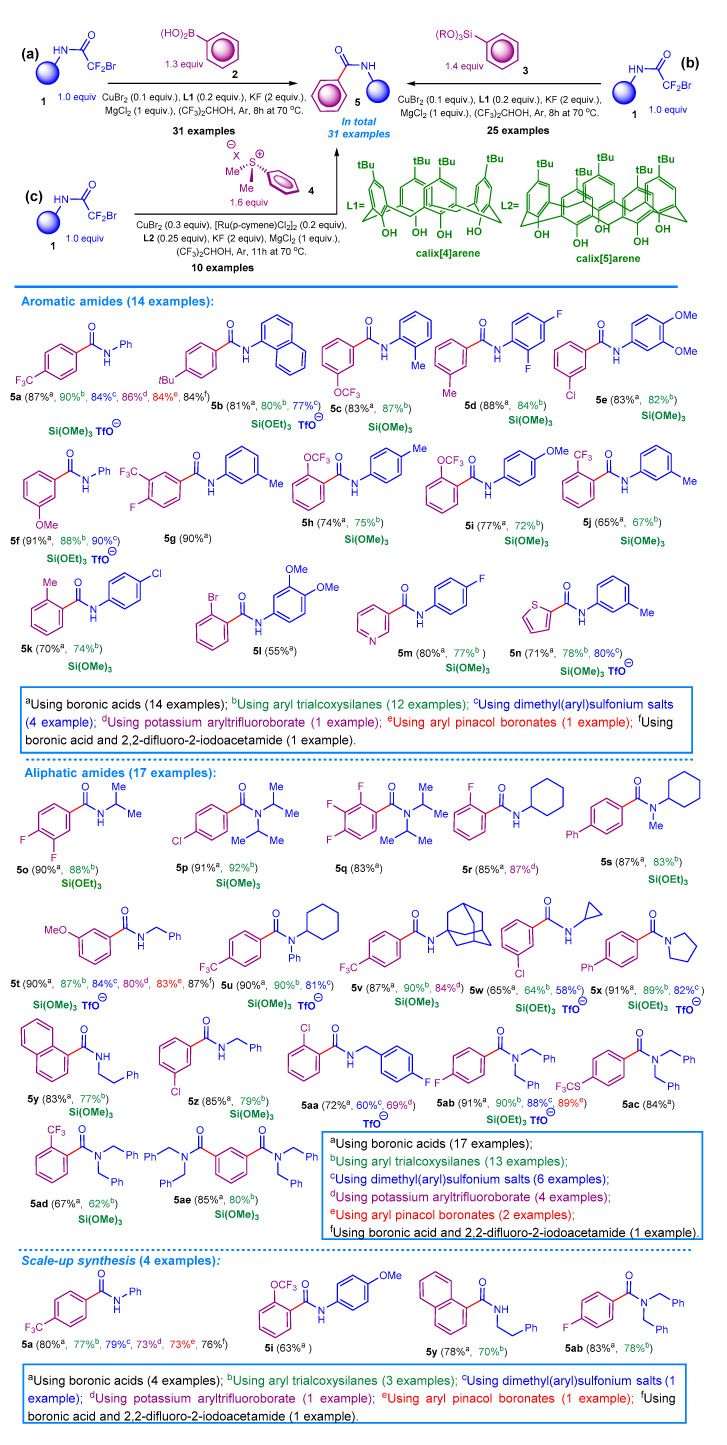
(**a**) Reactions of 2-bromo-2,2-difluoro-acetamides with aryl boronic acid, (**b**) Reactions of 2-bromo-2,2-difluoro-acetamides with aryl trialkoxysilanes, (**c**) Reactions of 2-bromo-2,2-difluoro-acetamides with dimethyl-aryl-sulfonium triflates. Product scope of amides using developed synthetic protocols.

Focusing first on the reactions utilizing aryl boronic acids and aryl trialkoxysilanes, these synthetic protocols were tolerant to numerous functional groups placed on both coupling partners. In particular, both methodologies allowed the coupling of aryl substrates bearing a vast range of electron-withdrawing and electron-donating substituents placed in ortho-, meta-, and para- positions, respectively; among those are alkyl groups, alkoxy groups, Ph, halogens including fluorine, as well as CF_3_, CF_3_O, and CF_3_S groups. Substrates bearing 1-naphthyl, 1-thiophenyl, and 3-pyridyl moieties also showed excellent efficiency with some discrepancy for the formation of the thionyl derivative **5n** (Scheme 3). Interestingly, both protocols were operational for aryl substrates bearing diverse ortho substituents (Me, F, Cl, Br, CF_3_, CF_3_O). Of note, highly fluorinated boronic acids and aryl trialkoxysilanes were prone to enter those protocols readily delivering the corresponding amides **5g**, **5o**, **5q**. Regarding the reactivity of 2-bromo-2,2-difluoroacetamide counterparts **1**, we did not observe any influence on the reaction efficiency of the substituents placed on the amide nitrogen—both alkyl and aryl groups as well as mixed derivatives exerted excellent tolerability within the developed protocols (Scheme 3). These reactions were not affected by changing a substitution pattern on the 2-bromo-2,2-difluoroacetamides: Species with alkyl as well as aryl substituents on the amide motif were equally effective within both synthetic protocols (Scheme 2). To further demonstrate the synthetic utility of these methodologies, the gram-scale reactions were successfully performed using 10 mmol of the 2-bromo-2,2-difluoroacetamides, which yielded the expected products in high yields. 

To the general scope and limitations, it is also important to note: (1) Within both described syntactic protocols we tried numerous other N-substituted and N-unsubstituted derivatives of 2-bromo-2,2-difluoroacetic acid, for instance: 2-bromo-2,2-difluoroethane-thioamides, 2-bromo-2,2-difluoroacetimidamides, 2-bromo-2,2-difluoroacetohydrazonamides; all these substrates were not prone to enter the developed arylation protocols; (2) Aryl pinacol borates as well as aryl trifluoroborates in the form of potassium salts act as arylation agents in the frames of both synthetic protocols (2 and 4 examples respectively, Scheme 3); (3) 2,2-Difluoro-2-iodoacetamides exerted similar activity as the corresponding bromo derivatives (2 examples, Scheme 3). 

As the final accord of this work, we turned our attention to aryl sulphonium salts **4**. These are donors of aryl groups and are often considered as equivalents of aryl halides, possessing low reduction potentials [46,47,48]. We assumed that those species might have capacity to enter the title synthetic protocol (Scheme 2c). These compounds did not react well under previously optimized reaction conditions, where the model compound **5a** was obtained in 47% yield (Table 3, Entry 1). Thus, we embarked once more on the search for new operational reaction conditions for the model reaction. It is worthwhile to note that in the case of this reaction, we had to increase the amount of copper salt to 0.3 equiv. and add 0.2 equiv. of [Ru(p-cymene)Cl_2_]_2_, which was superior to other TM co-catalysts (Table 3). Finally, by employing CuBr_2_ (0.3 equiv.), [Ru(p-cymene)Cl_2_]_2_ (0.2 equiv.), KF (2 equiv.), MgCl_2_ (1 equiv.) and 0.25 equiv. of calix[5]arene derivative (**L2**), in hexafluoropropanol, the model amide **5a** was prepared in 84% yield. Further study of the scope resulted in the preparation of ten amides in total (Scheme 3c).

To gain the insight to the reaction mechanism, we performed several control experiments: (a) Reactions without addition of calixarenes; (b) reactions without CuBr_2_ and MgCl_2_; (c) reactions in the dark and (d) reactions with 2 equiv. and 3 equiv. of TEMPO, which led to the modest decrease of the yield of title model amid compound. All these experiments are depicted in the Table 1, Table 2 and Table 3.

## 3. Materials and Methods

Commercially available starting materials, reagents, catalysts, anhydrous, and degassed solvents were used without further purification. Flash column chromatography was performed with Merck Silica gel 60 (230–400 mesh). The solvents for column chromatography were distilled before the use. Thin layer chromatography was carried out using Merck TLC Silica gel 60 F254 and visualized by short-wavelength ultraviolet light or by treatment with potassium permanganate (KMnO_4_) stain. ^1^H, ^13^C, and ^19^F-NMR spectra were recorded on a Bruker 250 and 500 MHz at 20 °C. All **^1^**H-NMR spectra are reported in parts per million (ppm) downfield of TMS and were measured relative to the signals for CHCl3 (7.26 ppm) and DMSO (2.50 ppm). All ^13^C{^1^H}-NMR spectra were reported in ppm relative to residual CHCl_3_ (77.00 ppm) or DMSO (39.70 ppm) and were obtained with ^1^H decoupling. Coupling constants, J, are reported in Hertz (Hz). Gas chromatographic analyses was performed on Gas Chromatograph Mass Spectrometer GCMS-QP2010 Ultra instrument.

The optimal reaction conditions were identified by microscale high-†hroughput experimentation screening. Parallel synthesis was accomplished in an MBraun glovebox operating with a constant Ar-purge (oxygen and water <5 ppm). Screening reactions were carried out in 10 mL vials using suitable heating blocks. Liquid chemicals were dosed using gas tight micro syringes. Isolation of obtained compounds was achieved by column chromatography on Silica gel.

All used boronic acids **2** and some aryl trialkoxysilanes **3** are commercially available and were purchased from appropriate vendors. 2-Bromo-2,2-difluoroacetamides [49,50,51,52,53,54,55,56,57], 1, 2-iodo-2,2-difluoroacetamides [54], aryl trialkoxysilanes **3** [58,59,60,61,62,63,64], sulfonium salts **4** [65,66,67], and calixarenes **L1**, **L2** [68,69] are known compounds in the literature and were prepared according to the known literature, and the spectral data are identical with the corresponding literature. Copies ^1^H and ^13^C-NMR spectra are placed in Supplementary Materials.

*General procedure for the synthesis of amides **5** by the reaction of 2-bromo-2,2-difluoroacetamides **1** with aryl boronic acids **2**.*

Under inert atmosphere (glovebox operating with a constant Ar-purge), to an 18 mL ACE pressure tube equipped with a stir bar, consequently, an appropriate 2-bromo-2,2-difluoroacetamide (1.0 mmol, 1.0 equiv.), KF (116 mg, 2.0 mmol, 2.0 equiv.), MgCl_2_ (95 mg, 1.0 mmol, 1.0 equiv.), appropriate aryl boronic acid (1.3 mmol, 1.3 equiv.), the **L1** (0.2 mmol, 0.2 equiv.), and finally CuBr_2_ (0.1 mmol, 0.1 equiv.) were placed; then the hexafluoropropanol (0.12 mmol/mL) was added and the reaction vessel was properly capped by Teflon stopper. Finally, the reaction vessel was removed from the glovebox and subjected to heating under vigorous stirring for 8 h. The progress of the reaction was controlled by TLC. After completion, the reaction mixture was evaporated until it reached dryness using a rotary evaporator, the content of the flask was generously treated with distilled water, filtered, and finally properly dried in vacuum. The resulting crude was directly subjected to gradient flash chromatography on silica gel using a mixture of hexane/ethyl acetate as eluent to isolate the desired amide derivative.

The gram scale synthesis was performed on 10 mmol of the starting 2-bromo-2,2-difluoroacetamide.

*General procedure for the synthesis of amides **5** by the reaction of 2-bromo-2,2-difluoroacetamides 1 with aryl trialkoxysilanes **3**.*

Under inert atmosphere (glovebox operating with a constant Ar-purge), to an 18 mL ACE pressure tube equipped with a stir bar, an appropriate 2-bromo-2,2-difluoroacetamide (1.0 mmol, 1.0 equiv.), KF (116 mg, 2.0 mmol, 2.0 equiv.), MgCl_2_ (95 mg, 1.0 mmol, 1.0 equiv.), appropriate trialkoxysilane (1.4 mmol, 1.4 equiv.), the **L1** (0.2 mmol, 0.2 equiv.), and finally CuBr_2_ (0.1 mmol, 0.1 equiv.) was consequently placed; then the hexafluoropropanol (0.12 mmol/mL) was added and the reaction vessel was properly capped by Teflon stopper. Finally, the reaction vessel was removed from the glovebox and subjected to heating under vigorous stirring for 8 h. The progress of the reaction was controlled by TLC. After completion, the reaction mixture was evaporated until it reached dryness using a rotary evaporator, the content of the flask was generously treated with distilled water, filtered, and finally properly dried in vacuum. The resulting crude was directly subjected to gradient flash chromatography on silica gel using a mixture of hexane/ethyl acetate as eluent to isolate the desired amide derivative.

The gram scale synthesis was performed on 10 mmol of the starting 2-bromo-2,2-difluoroacetamide.

*General procedure for the synthesis of amides **5** by the reaction of 2-bromo-2,2-difluoroacetamides **1** with (aryl)dimethylsulfonium salts **4**.*

Under inert atmosphere (glovebox operating with a constant Ar-purge), to an 18 mL ACE pressure equipped with a stir bar, an appropriate 2-bromo-2,2-difluoroacetamide (1.0 mmol, 1.0 equiv.), KF (116 mg, 2.0 mmol, 2.0 equiv.), MgCl_2_ (95 mg, 1.0 mmol, 1.0 equiv.), aryl sulphonium salt (1.6 mmol, 1.6 equiv.), the **L2** (0.25 mmol, 0.25 equiv.), [Ru(p-cymene)Cl_2_]_2_ (0.2 mmol, 0.2 equiv.), and finally CuBr_2_ (0.3 mmol, 0.3 equiv.) was consequently placed; then the hexafluoropropanol (0.12 mmol/mL) was added and the reaction vessel was properly capped by Teflon stopper. Finally, the reaction vessel was removed from the glovebox and subjected to heating under vigorous stirring for 11 h. The progress of the reaction was controlled by TLC. After completion, the reaction mixture was evaporated until it reached dryness using rotary evaporator, the content of the flask was generously treated with distilled water, filtered, and finally properly dried in vacuum. The resulting crude was directly subjected to gradient flash chromatography on silica gel using a mixture of hexane/ethyl acetate as eluent to isolate the desired amide derivative. The gram scale synthesis was performed on 10 mmol of the starting 2-bromo-2,2-difluoroacetamide.

***N-phenyl-4-(trifluoromethyl)benzamide 5a.*** The title compound was prepared, starting with 2-bromo-2,2-difluoroacetamide **1a** (250 mg, 1.0 mmol, 1.0 equiv.), KF (116 mg, 2.0 mmol, 2.0 equiv.), MgCl_2_ (95 mg, 1.0 mmol, 1.0 equiv.), aryl boronic acid **2n** (247 mg, 1.3 mmol, 1.3 equiv.), **L1** (130 mg, 0.2 mmol, 0.2 equiv.), CuBr_2_ (22.3 mg, 0.1 mmol, 0.1 equiv.), and hexafluoropropanol (0.12 mmol/mL). The purification of the dry crude performed by column chromatography on silica gel provides the amide **5a** (231 mg, 0.87 mmol, 87%). The gram scale synthesis was performed on 10 mmol of the starting 2-bromo-2,2-difluoroacetamide **1a** and the amide **5a** was prepared in 80% yield (2.12 g, 8 mmol). Alternatively, the title compound was prepared starting with an appropriate 2-bromo-2,2-difluoroacetamide **1a** (250 mg, 1.0 mmol, 1.0 equiv.), KF (116 mg, 2.0 mmol, 2.0 equiv.), MgCl_2_ (95 mg, 1.0 mmol, 1.0 equiv.), appropriate trialkoxysilane **3k** (372 mg, 1.4 mmol, 1.4 equiv.), the **L1** (130 mg, 0.2 mmol, 0.2 equiv.), CuBr_2_ (22.3 mg, 0.1 mmol, 0.1 equiv.), and hexafluoropropanol (0.12 mmol/mL). The purification of the dry crude performed by column chromatography on silica gel provides the amide **5a** (239 mg, 0.90 mmol, 90%). The gram scale synthesis was performed on 10 mmol of the starting 2-bromo-2,2-difluoroacetamide **1a** and the amide **5a** was prepared in 77% yield (2.04 g, 7.7 mmol).

Alternatively, the title compound was prepared starting with an appropriate 2-bromo-2,2-difluoroacetamide **1a** (250 mg, 1.0 mmol, 1.0 equiv.), KF (116 mg, 2.0 mmol, 2.0 equiv.), MgCl_2_ (95 mg, 1.0 mmol, 1.0 equiv.), aryl sulphonium salt **4a** (584 mg, 1.6 mmol, 1.6 equiv.), the **L2** (202 mg, 0.25 mmol, 0.25 equiv.), [Ru(p-cymene)Cl_2_]_2_ (122 mg, 0.2 mmol, 0.2 equiv.), CuBr_2_ (67 mg, 0.3 mmol, 0.3 equiv.), and hexafluoropropanol (0.12 mmol/mL). The purification of the dry crude performed by column chromatography on silica gel provides the amide **5a** (222 mg, 0.84 mmol, 84%). The gram scale synthesis was performed on 10 mmol of the starting 2-bromo-2,2-difluoroacetamide **1a** and the amide **5a** was prepared in 79% yield (2.09 g, 7.9 mmol).

Alternatively, the title compound was prepared starting with an appropriate 2-bromo-2,2-difluoroacetamide **1a** (250 mg, 1.0 mmol, 1.0 equiv.), KF (116 mg, 2.0 mmol, 2.0 equiv.), MgCl_2_ (95 mg, 1.0 mmol, 1.0 equiv.), appropriate potassium trifluoro(4-(trifluoromethyl)phenyl)borate (328 mg, 1.3 mmol, 1.3 equiv.), the **L1** (130 mg, 0.2 mmol, 0.2 equiv.), CuBr_2_ (22.3 mg, 0.1 mmol, 0.1 equiv.), and hexafluoropropanol (0.12 mmol/mL). The purification of the dry crude performed by column chromatography on silica gel provides the amide **5a** (228 mg, 0.86 mmol, 86%). The gram scale synthesis was performed on 10 mmol of the starting 2-bromo-2,2-difluoroacetamide **1a** and the amide **5a** was prepared in 73% yield (1.93 g, 7.3 mmol).

Alternatively, the title compound was prepared starting with an appropriate 2-bromo-2,2-difluoroacetamide **1a** (250 mg, 1.0 mmol, 1.0 equiv.), KF (116 mg, 2.0 mmol, 2.0 equiv.), MgCl_2_ (95 mg, 1.0 mmol, 1.0 equiv.), appropriate 4,4,5,5-tetramethyl-2-(4-(trifluoromethyl)phenyl)-1,3,2-dioxaborolane (354 mg, 1.3 mmol, 1.3 equiv.), the **L1** (130 mg, 0.2 mmol, 0.2 equiv.), CuBr_2_ (22.3 mg, 0.1 mmol, 0.1 equiv.), and hexafluoropropanol (0.12 mmol/mL). The purification of the dry crude performed by column chromatography on silica gel provides the amide **5a** (222 mg, 0.84 mmol, 84%). The gram scale synthesis was performed on 10 mmol of the starting 2-bromo-2,2-difluoroacetamide **1a** and the amide **5a** was prepared in 73% yield (1.93 g, 7.3 mmol).

Alternatively, the title compound was prepared starting with an appropriate 2,2-difluoro-2-iodo-N-phenylacetamide (297 mg, 1.0 mmol, 1.0 equiv.), KF (116 mg, 2.0 mmol, 2.0 equiv.), MgCl_2_ (95 mg, 1.0 mmol, 1.0 equiv.), appropriate aryl boronic acid **2n** (247 mg, 1.3 mmol, 1.3 equiv.), the **L1** (130 mg, 0.2 mmol, 0.2 equiv.), CuBr_2_ (22.3 mg, 0.1 mmol, 0.1 equiv.), and hexafluoropropanol (0.12 mmol/mL). The purification of the dry crude performed by column chromatography on silica gel provides the amide **5a** (222 mg, 0.84 mmol, 84%). The gram scale synthesis was performed on 10 mmol of the starting 2-bromo-2,2-difluoroacetamide **1a** and the amide **5a** was prepared in 76% yield (2.01 g, 7.6 mmol).

Flash column chromatography was performed using a mixture of hexane/ethyl acetate 3:1 as an eluent to provide the corresponding amide product.

White solid, mp 184–185 °C. **^1^H-NMR** (500 MHz, DMSO-*d_6_*): δ 7.12 (t, 1H, ^3^*J* = 7.3 Hz, CH_Ar_), 7.37 (t, 2H, ^3^*J* = 8.3 Hz, CH_Ar_), 7.80 (d, 2H, ^3^*J* = 7.6 Hz, CH_Ar_), 7.89 (d, 2H, ^3^*J* = 8.2 Hz, CH_Ar_), 8.16 (d, 2H, ^3^*J* = 8.1 Hz, CH_Ar_), 10.5 (s, 1H, NH).

**^13^C{^1^H}-NMR** (126 MHz, DMSO-*d_6_*): δ 120.5, 123.9 (q, ^1^*J_CF_* = 273.8 Hz, CF_3_), 124.0, 125.4 (d, *J_CF_* = 3.1 Hz), 128.6, 128.7, 131.4 (q, ^2^*J_CF_* = 30.3 Hz, *C*CF_3_), 138.8 (d, *J_CF_* = 11.3 Hz), 164.4.

HRMS (TOF MS ES+): Calcd for C_14_H_11_NOF_3_ (M+H) 266.0809. Found 266.0793.

***4-(tert-butyl)-N-(naphthalen-1-yl)benzamide* 5b.** The title compound was prepared starting with an appropriate 2-bromo-2,2-difluoroacetamide **1e** (300 mg, 1.0 mmol, 1.0 equiv.), KF (116 mg, 2.0 mmol, 2.0 equiv.), MgCl_2_ (95 mg, 1.0 mmol, 1.0 equiv.), appropriate aryl boronic acid **2a** (231 mg, 1.3 mmol, 1.3 equiv.), the **L1** (130 mg, 0.2 mmol, 0.2 equiv.), CuBr_2_ (22.3 mg, 0.1 mmol, 0.1 equiv.), and hexafluoropropanol (0.12 mmol/mL). The purification of the dry crude performed by column chromatography on silica gel provides the amide **5b** (245 mg, 0.81 mmol, 81%).

Alternatively, the title compound was prepared starting with an appropriate 2-bromo-2,2-difluoroacetamide **1e** (300 mg, 1.0 mmol, 1.0 equiv.), KF (116 mg, 2.0 mmol, 2.0 equiv.), MgCl_2_ (95 mg, 1.0 mmol, 1.0 equiv.), appropriate trialkoxysilane **3a** (414 mg, 1.4 mmol, 1.4 equiv.), the **L1** (130 mg, 0.2 mmol, 0.2 equiv.), CuBr_2_ (22.3 mg, 0.1 mmol, 0.1 equiv.), and hexafluoropropanol (0.12 mmol/mL). The purification of the dry crude performed by column chromatography on silica gel provides the amide **5b** (242 mg, 0.80 mmol, 80%).

Alternatively, the title compound was prepared starting with an appropriate 2-bromo-2,2-difluoroacetamide **1e** (300 mg, 1.0 mmol, 1.0 equiv.), KF (116 mg, 2.0 mmol, 2.0 equiv.), MgCl_2_ (95 mg, 1.0 mmol, 1.0 equiv.), aryl sulphonium salt **4h** (312 mg, 1.6 mmol, 1.6 equiv.), the **L2** (202 mg, 0.25 mmol, 0.25 equiv.), [Ru(p-cymene)Cl_2_]_2_ (122 mg, 0.2 mmol, 0.2 equiv.), CuBr_2_ (67 mg, 0.3 mmol, 0.3 equiv.), and hexafluoropropanol (0.12 mmol/mL). The purification of the dry crude performed by column chromatography on silica gel provides the amide **5b** (233 mg, 0.77 mmol, 77%).

Flash column chromatography was performed using a mixture of hexane/ethyl acetate 3:1 as an eluent to provide the corresponding amide product.

White solid, mp 146–147 °C. **^1^H-NMR** (500 MHz, CDCl_3_): δ 1.38 (s, 9H, *t*Bu), 7.46–7.51 (m, 5H, CH_Ar_), 7.72 (d, 1H, ^3^*J* = 8.4 Hz, CH_Ar_), 7.87–7.90 (m, 3H, CH_Ar_), 7.92 (s, 1H, CH_Ar_), 7.97 (br. s, 1H, CH_Ar_), 8.33 (s, 1H, NH).

**^13^C{^1^H}-NMR** (126 MHz, CDCl_3_): δ 31.2, 35.0, 120.79, 120.80, 121.2, 125.7, 125.9, 126.3, 127.1, 127.5, 127.51, 128.7, 131.9, 132.5, 134.1.

HRMS (TOF MS ES+): Calcd for C_21_H_22_NO (M + H) 304.1707. Found 304.1701.

***N-(o-tolyl)-3-(trifluoromethoxy)benzamide 5c.*** The title compound was prepared starting with an appropriate 2-bromo-2,2-difluoroacetamide **1d** (264 mg, 1.0 mmol, 1.0 equiv.), KF (116 mg, 2.0 mmol, 2.0 equiv.), MgCl_2_ (95 mg, 1.0 mmol, 1.0 equiv.), appropriate aryl boronic acid **2r** (268 mg, 1.3 mmol, 1.3 equiv.), the **L1** (130 mg, 0.2 mmol, 0.2 equiv.), CuBr_2_ (22.3 mg, 0.1 mmol, 0.1 equiv.), and hexafluoropropanol (0.12 mmol/mL). The purification of the dry crude performed by column chromatography on silica gel provides the amide **5c** (245 mg, 0.83 mmol, 83%).

Alternatively, the title compound was prepared starting with an appropriate 2-bromo-2,2-difluoroacetamide **1d** (264 mg, 1.0 mmol, 1.0 equiv.), KF (116 mg, 2.0 mmol, 2.0 equiv.), MgCl_2_ (95 mg, 1.0 mmol, 1.0 equiv.), appropriate trialkoxysilane **3n** (395 mg, 1.4 mmol, 1.4 equiv.), the **L1** (130 mg, 0.2 mmol, 0.2 equiv.) CuBr_2_ (22.3 mg, 0.1 mmol, 0.1 equiv.), and hexafluoropropanol (0.12 mmol/mL). The purification of the dry crude performed by column chromatography on silica gel provides the amide **5c** (257 mg, 0.87 mmol, 87%).

Flash column chromatography was performed using a mixture of hexane/ethyl acetate 5:1 as an eluent to provide the corresponding amide product.

White solid, mp 94–95 °C. **^1^H-NMR** (500 MHz, CDCl_3_): δ 2.30 (s, 3H, Me), 7.12–7.15 (m, 1H, CH_Ar_), 7.21–7.24 (m, 2H, CH_Ar_), 7.40 (d, 1H, ^3^*J* = 8.1 Hz, CH_Ar_), 7.50 (t, 1H, ^3^*J* = 7.9 Hz, CH_Ar_), 7.74–7.78 (m, 3H, CH_Ar_), 7.81 (br s, 1H, NH).

**^13^C{^1^H}-NMR** (126 MHz, CDCl_3_): δ 17.8, 120.1, 120.4 (q, ^1^*J_CF_* = 258.6 Hz), 123.6, 124.1, 125.1, 125.9, 126.8, 130.0, 130.2, 130.6, 135.2, 137.0, 149.5, 164.2.

HRMS (TOF MS ES+): Calcd for C_15_H_13_NO_2_F_3_ (M + H) 296.0901. Found 296.0898.

***N-(2,4-difluorophenyl)-3-methylbenzamide 5d.*** The title compound was prepared starting with an appropriate 2-bromo-2,2-difluoroacetamide **1j** (286 mg, 1.0 mmol, 1.0 equiv.), KF (116 mg, 2.0 mmol, 2.0 equiv.), MgCl_2_ (95 mg, 1.0 mmol, 1.0 equiv.), appropriate aryl boronic acid **2b** (177 mg, 1.3 mmol, 1.3 equiv.), the **L1** (130 mg, 0.2 mmol, 0.2 equiv.), CuBr_2_ (22.3 mg, 0.1 mmol, 0.1 equiv.), and hexafluoropropanol (0.12 mmol/mL). The purification of the dry crude performed by column chromatography on silica gel provides the amide **5d** (217 mg, 0.88 mmol, 88%).

Alternatively, the title compound was prepared starting with an appropriate 2-bromo-2,2-difluoroacetamide **1j** (286 mg, 1.0 mmol, 1.0 equiv.), KF (116 mg, 2.0 mmol, 2.0 equiv.), MgCl_2_ (95 mg, 1.0 mmol, 1.0 equiv.), appropriate trialkoxysilane **3b** (297 mg, 1.4 mmol, 1.4 equiv.), the **L1** (130 mg, 0.2 mmol, 0.2 equiv.), CuBr_2_ (22.3 mg, 0.1 mmol, 0.1 equiv.), and hexafluoropropanol (0.12 mmol/mL). The purification of the dry crude performed by column chromatography on silica gel provides the amide **5d** (207 mg, 0.84 mmol, 84%).

Flash column chromatography was performed using a mixture of hexane/ethyl acetate 4:1 as an eluent to provide the corresponding amide product.

White solid, mp 106–107 °C. **^1^H-NMR** (500 MHz, CDCl_3_): δ 2.41 (s, 3H, Me), 6.85–6.90 (m, 2H, CH_Ar_), 7.35–7.36 (m, 2H, CH_Ar_), 7.63–7.65 (m, 1H, CH_Ar_), 7.68 (s, 1H, CH_Ar_), 8.02 (br s, 1H, NH), 8.29–8.33 (m, 1H, CH_Ar_).

**^13^C{^1^H}-NMR** (126 MHz, CDCl_3_): δ 21.4, 103.4 (d, *J_CF_* = 26.5 Hz), 103.6 (d, *J_CF_* = 26.5 Hz), 111.2 (dd, *J_CF_* = 21.7 Hz, *J_CF_* = 3.6 Hz), 122.6 (dd, *J_CF_* = 10.5 Hz, *J_CF_* = 3.7 Hz), 123.1 (d, *J_CF_* = 9.3 Hz), 124.0, 127.8, 128.6, 132.9, 134.1, 138.7, 152.9 (dd, ^1^*J_CF_* = 246.4 Hz, *J_CF_* = 11.9 Hz), 158.6 (dd, ^1^*J_CF_* = 246.5 Hz, *J_CF_* = 11.4 Hz), 165.7.

HRMS (TOF MS ES+): Calcd for C_14_H_12_NOF_2_ (M + H) 248.0894. Found 248.0898.

***3-chloro-N-(3,4-dimethoxyphenyl)benzamide 5e.*** The title compound was prepared starting with an appropriate 2-bromo-2,2-difluoroacetamide **1g** (310 mg, 1.0 mmol, 1.0 equiv.), KF (116 mg, 2.0 mmol, 2.0 equiv.), MgCl_2_ (95 mg, 1.0 mmol, 1.0 equiv.), appropriate aryl boronic acid **2a** (203 mg, 1.3 mmol, 1.3 equiv.), the **L1** (130 mg, 0.2 mmol, 0.2 equiv.), CuBr_2_ (22.3 mg, 0.1 mmol, 0.1 equiv.), and hexafluoropropanol (0.12 mmol/mL). The purification of the dry crude performed by column chromatography on silica gel provides the amide **5e** (242 mg, 0.83 mmol, 83%).

Alternatively, the title compound was prepared starting with an appropriate 2-bromo-2,2-difluoroacetamide **1g** (310 mg, 1.0 mmol, 1.0 equiv.), KF (116 mg, 2.0 mmol, 2.0 equiv.), MgCl_2_ (95 mg, 1.0 mmol, 1.0 equiv.), appropriate trialkoxysilane **3i** (326 mg, 1.4 mmol, 1.4 equiv.), the **L1** (130 mg, 0.2 mmol, 0.2 equiv.), CuBr_2_ (22.3 mg, 0.1 mmol, 0.1 equiv.), and hexafluoropropanol (0.12 mmol/mL). The purification of the dry crude performed by column chromatography on silica gel provides the amide **5e** (239 mg, 0.82 mmol, 82%).

Flash column chromatography was performed using a mixture of hexane/ethyl acetate 4:1 as an eluent to provide the corresponding amide product.

Light purple, solid mp 127–128 °C. **^1^H-NMR** (500 MHz, CDCl_3_): δ 3.79 (s, 3H, OMe), 3.83 (s, 3H, OMe), 6.77 (d, 1H, ^3^*J* = 8.4 Hz, CH_Ar_), 7.02 (dd, 1H, ^3^*J* = 8.7 Hz, ^4^*J* = 2.1 Hz, CH_Ar_), 7.32 (t, 1H, ^3^*J* = 7.9 Hz, CH_Ar_), 7.36–7.37 (m, 1H, CH_Ar_), 7.44 (dd, 1H, ^3^*J* = 8.0 Hz, ^4^*J* = 1.0 Hz, CH_Ar_). 7.70 (d, 1H, ^3^*J* = 7.0 Hz, CH_Ar_), 7.81 (s, 1H, CH_Ar_), 8.26 (s, 1H, NH).

**^13^C{^1^H}-NMR** (126 MHz, CDCl_3_): δ 55.7, 55.9, 105.2, 111.1, 112.6, 125.1, 127.3, 129.9, 131.1, 131.6, 134.7, 136.6, 146.1, 148.8, 164.5.

HRMS (TOF MS ES+): Calcd for C_15_H_15_NO_3_Cl (M + H) 292.0738. Found 292.0740.

***3-methoxy-N-phenylbenzamide 5f.*** The title compound was prepared starting with an appropriate 2-bromo-2,2-difluoroacetamide **1a** (250 mg, 1.0 mmol, 1.0 equiv.), KF (116 mg, 2.0 mmol, 2.0 equiv.), MgCl_2_ (95 mg, 1.0 mmol, 1.0 equiv.), appropriate aryl boronic acid **2w** (198 mg, 1.3 mmol, 1.3 equiv.), the **L1** (130 mg, 0.2 mmol, 0.2 equiv.), CuBr_2_ (22.3 mg, 0.1 mmol, 0.1 equiv.), and hexafluoropropanol (0.12 mmol/mL). The purification of the dry crude performed by column chromatography on silica gel provides the amide **5f** (207 mg, 0.91 mmol, 91%).

Alternatively, the title compound was prepared starting with an appropriate 2-bromo-2,2-difluoroacetamide **1a** (250 mg, 1.0 mmol, 1.0 equiv.), KF (116 mg, 2.0 mmol, 2.0 equiv.), MgCl_2_ (95 mg, 1.0 mmol, 1.0 equiv.), appropriate trialkoxysilane **3j** (378 mg, 1.4 mmol, 1.4 equiv.), the **L1** (130 mg, 0.2 mmol, 0.2 equiv.), CuBr_2_ (22.3 mg, 0.1 mmol, 0.1 equiv.), and hexafluoropropanol (0.12 mmol/mL). The purification of the dry crude performed by column chromatography on silica gel provides the amide **5f** (200 mg, 0.88 mmol, 88%).

Alternatively, the title compound was prepared starting with an appropriate 2-bromo-2,2-difluoroacetamide **1a** (250 mg, 1.0 mmol, 1.0 equiv.), KF (116 mg, 2.0 mmol, 2.0 equiv.), MgCl_2_ (95 mg, 1.0 mmol, 1.0 equiv.), aryl sulphonium salt **4b** (410 mg, 1.6 mmol, 1.6 equiv.), the **L2** (202 mg, 0.25 mmol, 0.25 equiv.), [Ru(p-cymene)Cl_2_]_2_ (122 mg, 0.2 mmol, 0.2 equiv.), CuBr_2_ (67 mg, 0.3 mmol, 0.3 equiv.), and hexafluoropropanol (0.12 mmol/mL). The purification of the dry crude performed by column chromatography on silica gel provides the amide **5f** (204 mg, 0.90 mmol, 90%).

Flash column chromatography was performed using a mixture of hexane/ethyl acetate 4:1 as an eluent to provide the corresponding amide product.

White solid, mp 116–117 °C. **^1^H-NMR** (500 MHz, CDCl_3_): δ 3.78 (s, 3H, OMe), 7.02 (dd, 1H, ^3^*J* = 8.2 Hz, ^4^*J* = 2.5 Hz, CH_Ar_), 7.13 (t, 1H, ^3^*J* = 7.5 Hz, CH_Ar_), 7.28–7.40 (m, 5H, CH_Ar_), 7.64 (d, 2H, ^3^*J* = 7.9 Hz, CH_Ar_), 8.23 (s, 1H, NH).

**^13^C{^1^H}-NMR** (126 MHz, CDCl_3_): δ 55.3, 112.3, 117.9, 118.8, 120.3, 124.5, 128.9, 129.6, 136.3, 137.9, 159.8, 165.8.

HRMS (TOF MS ES+): Calcd for C_14_H_14_NO_2_ (M + H) 228.1025. Found 228.1025.

***4-fluoro-N-(m-tolyl)-3-(trifluoromethyl)benzamide 5g.*** The title compound was prepared starting with an appropriate 2-bromo-2,2-difluoroacetamide **1c** (264 mg, 1.0 mmol, 1.0 equiv.), KF (116 mg, 2.0 mmol, 2.0 equiv.), MgCl_2_ (95 mg, 1.0 mmol, 1.0 equiv.), appropriate aryl boronic acid **2p** (270 mg, 1.3 mmol, 1.3 equiv.), the **L1** (130 mg, 0.2 mmol, 0.2 equiv.), CuBr_2_ (22.3 mg, 0.1 mmol, 0.1 equiv.), and hexafluoropropanol (0.12 mmol/mL). The purification of the dry crude performed by column chromatography on silica gel provides the amide **5g** (267 mg, 0.90 mmol, 90%).

Flash column chromatography was performed using a mixture of hexane/ethyl acetate 5:1 as an eluent to provide the corresponding amide product.

Colorless solid, mp 121–122 °C. **^1^H-NMR** (500 MHz, DMSO-*d_6_*): δ 2.31 (s, 3H, Me), 6.94 (d, 1H, ^3^*J* = 7.4 Hz, CH_Ar_), 7.24 (d, 1H, ^3^*J* = 8.2 Hz, CH_Ar_), 7.56 (d, 2H, ^3^*J* = 8.3 Hz, CH_Ar_), 7.60 (s, 1H, CH_Ar_), 8.33–8.36 (m, 2H, CH_Ar_), 10.36 (s, 1H, NH),

**^13^C{^1^H}-NMR** (126 MHz, DMSO-*d_6_*): δ 21.2, 116.5 (dd, *J_CF_* = 33.5 Hz, *J_CF_* = 12.1 Hz), 117.5 (d, *J_CF_* = 20.8 Hz), 117.7, 121.1, 122.4 (q, ^1^*J_CF_* = 272.3 Hz, CF_3_), 124.8, 126.9, 128.5, 131.7 (d, *J_CF_* = 3.1 Hz), 135.1 (d, *J_CF_* = 9.4 Hz), 137.9, 138.7, 160.6 (d, ^1^*J_CF_* = 257.0 Hz), 163.0.

HRMS (TOF MS ES+): Calcd for C_15_H_12_NOF_4_ (M + H) 298.0861. Found 298.0855.

***N-(p-tolyl)-2-(trifluoromethoxy)benzamide 5h.*** The title compound was prepared starting with an appropriate 2-bromo-2,2-difluoroacetamide **1b** (264 mg, 1.0 mmol, 1.0 equiv.), KF (116 mg, 2.0 mmol, 2.0 equiv.), MgCl_2_ (95 mg, 1.0 mmol, 1.0 equiv.), appropriate aryl boronic acid **2q** (268 mg, 1.3 mmol, 1.3 equiv.), the **L1** (130 mg, 0.2 mmol, 0.2 equiv.), CuBr_2_ (22.3 mg, 0.1 mmol, 0.1 equiv.), and hexafluoropropanol (0.12 mmol/mL). The purification of the dry crude performed by column chromatography on silica gel provides the amide **5h** (218 mg, 0.74 mmol, 74%).

Alternatively, the title compound was prepared starting with an appropriate 2-bromo-2,2-difluoroacetamide **1b** (264 mg, 1.0 mmol, 1.0 equiv.), KF (116 mg, 2.0 mmol, 2.0 equiv.), MgCl_2_ (95 mg, 1.0 mmol, 1.0 equiv.), appropriate trialkoxysilane **3m** (395 mg, 1.4 mmol, 1.4 equiv.), the **L1** (130 mg, 0.2 mmol, 0.2 equiv.), CuBr_2_ (22.3 mg, 0.1 mmol, 0.1 equiv.), and hexafluoropropanol (0.12 mmol/mL). The purification of the dry crude performed by column chromatography on silica gel provides the amide **5h** (221 mg, 0.75 mmol, 75%).

Flash column chromatography was performed using a mixture of hexane/ethyl acetate 5:1 as an eluent to provide the corresponding amide product.

White solid, mp 108–109 °C. **^1^H-NMR** (500 MHz, CDCl_3_): δ 2.35 (s, 3H, Me), 7.18 (d, 2H, ^3^*J* = 8.0 Hz, CH_Ar_), 7.32 (d, 1H, ^3^*J* = 8.0 Hz, CH_Ar_), 7.42 (t, 1H, ^3^*J* = 7.5 Hz, CH_Ar_), 7.51–7.55 (m, 3H, CH_Ar_), 8.04 (d, 1H, ^3^*J* = 7.7 Hz, CH_Ar_), 8.30 (s, 1H, NH).

**^13^C{^1^H}-NMR** (126 MHz, CDCl_3_): δ 20.9, 120.3 (q, ^1^*J_CF_* = 261.0 Hz, OCF_3_), 120.4, 121.2, 127.6, 128.3, 129.6, 131.9, 132.6, 134.6, 135.0, 145.7, 162.1.

HRMS (TOF MS ES+): Calcd for C_15_H_13_NO_3_F_3_ (M + H) 312.0847. Found 312.0848.

***N-(4-methoxyphenyl)-2-(trifluoromethoxy)benzamide 5i.*** The title compound was prepared starting with an appropriate 2-bromo-2,2-difluoroacetamide **1f** (280 mg, 1.0 mmol, 1.0 equiv.), KF (116 mg, 2.0 mmol, 2.0 equiv.), MgCl_2_ (95 mg, 1.0 mmol, 1.0 equiv.), appropriate aryl boronic acid **2q** (268 mg, 1.3 mmol, 1.3 equiv.), the **L1** (130 mg, 0.2 mmol, 0.2 equiv.), CuBr_2_ (22.3 mg, 0.1 mmol, 0.1 equiv.), and hexafluoropropanol (0.12 mmol/mL). The purification of the dry crude performed by column chromatography on silica gel provides the amide **5i** (239 mg, 0.77 mmol, 77%). The gram scale synthesis was performed on 10 mmol of the starting 2-bromo-2,2-difluoroacetamide **1f** and the amide **5i** was prepared in 63% yield (1.96 g, 0.63 mmol).

Alternatively, the title compound was prepared starting with an appropriate 2-bromo-2,2-difluoroacetamide **1f** (280 mg, 1.0 mmol, 1.0 equiv.), KF (116 mg, 2.0 mmol, 2.0 equiv.), MgCl_2_ (95 mg, 1.0 mmol, 1.0 equiv.), appropriate trialckoxysilane **3m** (395 mg, 1.4 mmol, 1.4 equiv.), the **L1** (130 mg, 0.2 mmol, 0.2 equiv.), CuBr_2_ (22.3 mg, 0.1 mmol, 0.1 equiv.), and hexafluoropropanol (0.12 mmol/mL). The purification of the dry crude performed by column chromatography on silica gel provides the amide **5i** (224 mg, 0.72 mmol, 72%).

Flash column chromatography was performed using a mixture of hexane/ethyl acetate 6:1 as an eluent to provide the corresponding amide product.

Pink solid, mp 117–118 °C. **^1^H-NMR** (500 MHz, CDCl_3_): δ 3.81 (s, 3H, OMe), 6.88–6.92 (m, 2H, CH_Ar_), 7.32 (d, 1H, ^3^*J* = 8.2 Hz, CH_Ar_), 7.42 (dt, 1H, ^3^*J* = 7.6 Hz, ^4^*J* = 0.7 Hz, CH_Ar_), 7.51–7.54 (m, 3H, CH_Ar_), 8.02 (dd, 1H, ^3^*J* = 7.8 Hz, ^4^*J* = 1.7 Hz, CH_Ar_), 8.25 (s, 1H, NH).

**^13^C{^1^H}-NMR** (126 MHz, CDCl_3_): δ 55.5, 114.2, 120.3 (q, ^1^*J_CF_* = 260.4 Hz, OCF_3_), 121.2, 122.2, 127.6, 128.3, 130.6, 131.8, 132.5, 145.7, 156.8, 162.1.

HRMS (TOF MS ES+): Calcd for C_15_H_13_NO_2_F_3_ (M + H) 296.0904. Found 296.0898.

***N-(m-tolyl)-2-(trifluoromethyl)benzamide 5j.*** The title compound was prepared starting with an appropriate 2-bromo-2,2-difluoroacetamide **1c** (264 mg, 1.0 mmol, 1.0 equiv.), KF (116 mg, 2.0 mmol, 2.0 equiv.), MgCl_2_ (95 mg, 1.0 mmol, 1.0 equiv.), appropriate aryl boronic acid **2o** (247 mg, 1.3 mmol, 1.3 equiv.), the **L1** (130 mg, 0.2 mmol, 0.2 equiv.), CuBr_2_ (22.3 mg, 0.1 mmol, 0.1 equiv.), and hexafluoropropanol (0.12 mmol/mL). The purification of the dry crude performed by column chromatography on silica gel provides the amide **5j** (239 mg, 0.65 mmol, 65%).

Alternatively, the title compound was prepared starting with an appropriate 2-bromo-2,2-difluoroacetamide **1c** (264 mg, 1.0 mmol, 1.0 equiv.), KF (116 mg, 2.0 mmol, 2.0 equiv.), MgCl_2_ (95 mg, 1.0 mmol, 1.0 equiv.), appropriate trialkoxysilane **3m** (372 mg, 1.4 mmol, 1.4 equiv.), the **L1** (130 mg, 0.2 mmol, 0.2 equiv.), CuBr_2_ (22.3 mg, 0.1 mmol, 0.1 equiv.), and hexafluoropropanol (0.12 mmol/mL). The purification of the dry crude performed by column chromatography on silica gel provides the amide **5j** (224 mg, 0.67 mmol, 67%).

Flash column chromatography was performed using a mixture of hexane/ethyl acetate 5:1 as an eluent to provide the corresponding amide product.

Colorless solid, mp 120–121 °C. **^1^H-NMR** (500 MHz, CDCl_3_): δ 2.35 (s, 3H, Me), 6.98 (d, 1H, ^3^*J* = 7.57 Hz, CH_Ar_), 7.22 (t, 1H, ^3^*J* = 8.1 Hz, CH_Ar_), 7.33 (d, 1H, ^3^*J* = 7.6 Hz, CH_Ar_), 7.44 (s, 1H, CH_Ar_), 7.54–7.57 (m, 3H, CH_Ar_), 7.69–7.71 (m, 3H, NH, CH_Ar_).

**^13^C{^1^H}-NMR** (126 MHz, CDCl_3_): δ 21.4, 117.3, 120.8, 123.7 (q, ^1^*J_CF_* = 276.1 Hz, CF_3_), 125.7, 126.4 (q, *J_CF_* = 5.2 Hz, CF_3_), 127.1 (q, ^2^*J_CF_* = 31.5 Hz, *C*CF_3_), 128.5, 128.9, 130.0, 132.1, 135.7, 137.4, 139.0, 165.7.

HRMS (TOF MS ES+): Calcd for C_15_H_13_NOF_3_ (M + H) 280.0957. Found 280.0949.

***N-(4-chlorophenyl)-2-methylbenzamide 5k.*** The title compound was prepared starting with an appropriate 2-bromo-2,2-difluoroacetamide **1h** (285 mg, 1.0 mmol, 1.0 equiv.), KF (116 mg, 2.0 mmol, 2.0 equiv.), MgCl_2_ (95 mg, 1.0 mmol, 1.0 equiv.), appropriate aryl boronic acid **2c** (177 mg, 1.3 mmol, 1.3 equiv.), the **L1** (130 mg, 0.2 mmol, 0.2 equiv.), CuBr_2_ (22.3 mg, 0.1 mmol, 0.1 equiv.), and hexafluoropropanol (0.12 mmol/mL). The purification of the dry crude performed by column chromatography on silica gel provides the amide **5k** (172 mg, 0.70 mmol, 70%).

Alternatively, the title compound was prepared starting with an appropriate 2-bromo-2,2-difluoroacetamide **1h** (285 mg, 1.0 mmol, 1.0 equiv.), KF (116 mg, 2.0 mmol, 2.0 equiv.), MgCl_2_ (95 mg, 1.0 mmol, 1.0 equiv.), appropriate trialkoxysilane **3c** (297 mg, 1.4 mmol, 1.4 equiv.), the **L1** (130 mg, 0.2 mmol, 0.2 equiv.), CuBr_2_ (22.3 mg, 0.1 mmol, 0.1 equiv.), and hexafluoropropanol (0.12 mmol/mL). The purification of the dry crude performed by column chromatography on silica gel provides the amide **5k** (182 mg, 0.74 mmol, 74%).

Flash column chromatography was performed using a mixture of hexane/ethyl acetate 3:1 as an eluent to provide the corresponding amide product.

White solid, mp 136–138 °C. **^1^H-NMR** (500 MHz, CDCl_3_): δ 2.41 (s, 3H, Me), 7.17 (t, 1H, ^3^*J* = 7.2 Hz, CH_Ar_), 7.20 (d, 1H, ^3^*J* = 7.2 Hz, CH_Ar_), 7.25 (d, 2H, ^3^*J* = 8.7 Hz, CH_Ar_), 7.31 (dt, 2H, ^3^*J* = 7.6 Hz, ^4^*J* = 0.8 Hz, CH_Ar_), 7.36 (d, 1H, ^3^*J* = 7.2 Hz, CH_Ar_), 7.50 (d, 1H, ^3^*J* = 8.4 Hz, CH_Ar_), 7.87 (br s, 1H, NH).

**^13^C{^1^H}-NMR** (126 MHz, CDCl_3_): δ 19.7, 121.2, 125.8, 126.6, 129.0, 129.4, 130.3, 131.2, 135.9, 136.3, 136.5, 168.2.

HRMS (TOF MS ES+): Calcd for C_14_H_13_NOCl (M + H) 246.0690. Found 246.0686.

***2-bromo-N-(3,4-dimethoxyphenyl)benzamide 5l.*** The title compound was prepared starting with an appropriate 2-bromo-2,2-difluoroacetamide **1g** (310 mg, 1.0 mmol, 1.0 equiv.), KF (116 mg, 2.0 mmol, 2.0 equiv.), MgCl_2_ (95 mg, 1.0 mmol, 1.0 equiv.), appropriate aryl boronic acid **2m** (261 mg, 1.3 mmol, 1.3 equiv.), the **L1** (130 mg, 0.2 mmol, 0.2 equiv.), CuBr_2_ (22.3 mg, 0.1 mmol, 0.1 equiv.), and hexafluoropropanol (0.12 mmol/mL). The purification of the dry crude performed by column chromatography on silica gel provides the amide **5l** (185 mg, 0.55 mmol, 55%). Flash column chromatography was performed using a mixture of hexane/ethyl acetate 2:1 as an eluent to provide the corresponding amide product.

Purple solid, mp 140–141 °C. **^1^H-NMR** (500 MHz, CDCl_3_): δ 3.83 (s, 3H, OMe), 3.84 (s, 3H, OMe), 6.79 (d, 1H, ^3^*J* = 8.7 Hz, CH_Ar_), 7.00 (dd, 1H, ^3^*J* = 8.4 Hz, ^4^*J* = 2.6 Hz, CH_Ar_), 7.22–7.26 (m, 1H, CH_Ar_), 7.29–7.32 (m, 1H, CH_Ar_), 7.41 (d, 1H, ^4^*J* = 2.3 Hz, CH_Ar_), 7.51 (dd, 1H, ^3^*J* = 7.6 Hz, ^4^*J* = 1.6 Hz, CH_Ar_), 7.55 (d, 1H, ^3^*J* = 8.3 Hz, CH_Ar_), 7.96 (s, 1H, NH).

**^13^C{^1^H}-NMR** (126 MHz, CDCl_3_): δ 55.8, 56.0, 104.8, 111.2, 112.0, 119.2, 127.5, 129.4, 131.2, 131.4, 133.3, 137.7, 146.0, 148.9, 165.5.

HRMS (TOF MS ES+): Calcd for C_15_H_15_NO_3_Br (M + H) 266.0809. Found 266.0793.

***N-(4-fluorophenyl)nicotinamide 5m.*** The title compound was prepared starting with an appropriate 2-bromo-2,2-difluoroacetamide **1i** (268 mg, 1.0 mmol, 1.0 equiv.), KF (116 mg, 2.0 mmol, 2.0 equiv.), MgCl_2_ (95 mg, 1.0 mmol, 1.0 equiv.), appropriate aryl boronic acid **2t** (160 mg, 1.3 mmol, 1.3 equiv.), the **L1** (130 mg, 0.2 mmol, 0.2 equiv.), CuBr_2_ (22.3 mg, 0.1 mmol, 0.1 equiv.), and hexafluoropropanol (0.12 mmol/mL). The purification of the dry crude performed by column chromatography on silica gel provides the amide **5m** (173 mg, 0.80 mmol, 80%).

Alternatively, the title compound was prepared starting with an appropriate 2-bromo-2,2-difluoroacetamide **1i** (268 mg, 1.0 mmol, 1.0 equiv.), KF (116 mg, 2.0 mmol, 2.0 equiv.), MgCl_2_ (95 mg, 1.0 mmol, 1.0 equiv.), appropriate trialkoxysilane **3o** (277 mg, 1.4 mmol, 1.4 equiv.), the **L1** (130 mg, 0.2 mmol, 0.2 equiv.), CuBr_2_ (22.3 mg, 0.1 mmol, 0.1 equiv.), and hexafluoropropanol (0.12 mmol/mL). The purification of the dry crude performed by column chromatography on silica gel provides the amide **5m** (166 mg, 0.77 mmol, 77%).

Flash column chromatography was performed using a mixture of hexane/ethyl acetate 1:1 as an eluent to provide the corresponding amide product.

White solid, mp 130–131 °C. **^1^H-NMR** (500 MHz, CDCl_3_): δ 7.02 (t, 2H, ^3^*J* = 8.7 Hz, CH_Ar_), 7.34–7.37 (m, 1H, CH_Ar_), 7.55–7.58 (m, 2H, CH_Ar_), 8.15 (d, 1H, ^3^*J* = 8.3 Hz, CH_Ar_), 8.66 (dd, 1H, ^3^*J* = 4.7 Hz, ^4^*J* = 1.4 Hz, CH_Ar_), 8.80 (s, 1H, NH), 9.03 (s, 1H, CH_Ar_).

**^13^C{^1^H}-NMR** (126 MHz, CDCl_3_): δ 115.7 (d, *J_CF_* = 22.2 Hz), 122.5 (d, *J_CF_* = 7.0 Hz), 123.7, 130.6, 133.5, 135.6, 147.9, 152.2, 159.7 (d, ^1^*J_CF_* = 244.1 Hz), 164.1.

HRMS (TOF MS ES+): Calcd for C_15_H_12_NOF_3_Na (M + Na) 302.0769. Found 302.0769.

***N-(m-tolyl)thiophene-2-carboxamide 5n.*** The title compound was prepared starting with an appropriate 2-bromo-2,2-difluoroacetamide **1c** (264 mg, 1.0 mmol, 1.0 equiv.), KF (116 mg, 2.0 mmol, 2.0 equiv.), MgCl_2_ (95 mg, 1.0 mmol, 1.0 equiv.), appropriate aryl boronic acid **2u** (166 mg, 1.3 mmol, 1.3 equiv.), the **L1** (130 mg, 0.2 mmol, 0.2 equiv.), CuBr_2_ (22.3 mg, 0.1 mmol, 0.1 equiv.), and hexafluoropropanol (0.12 mmol/mL). The purification of the dry crude performed by column chromatography on silica gel provides the amide **5n** (154 mg, 0.71 mmol, 71%).

Alternatively, the title compound was prepared starting with an appropriate 2-bromo-2,2-difluoroacetamide **1c** (264 mg, 1.0 mmol, 1.0 equiv.), KF (116 mg, 2.0 mmol, 2.0 equiv.), MgCl_2_ (95 mg, 1.0 mmol, 1.0 equiv.), appropriate trialkoxysilane **3p** (286 mg, 1.4 mmol, 1.4 equiv.), the **L1** (130 mg, 0.2 mmol, 0.2 equiv.), CuBr_2_ (22.3 mg, 0.1 mmol, 0.1 equiv.), and hexafluoropropanol (0.12 mmol/mL). The purification of the dry crude performed by column chromatography on silica gel provides the amide **5n** (169 mg, 0.78 mmol, 78%).

Alternatively, the title compound was prepared starting with an appropriate 2-bromo-2,2-difluoroacetamide **1c** (264 mg, 1.0 mmol, 1.0 equiv.), KF (116 mg, 2.0 mmol, 2.0 equiv.), MgCl_2_ (95 mg, 1.0 mmol, 1.0 equiv.), aryl sulphonium salt **4c** (470 mg, 1.6 mmol, 1.6 equiv.), the **L2** (202 mg, 0.25 mmol, 0.25 equiv.), [Ru(p-cymene)Cl_2_]_2_ (122 mg, 0.2 mmol, 0.2 equiv.), CuBr_2_ (67 mg, 0.3 mmol, 0.3 equiv.), and hexafluoropropanol (0.12 mmol/mL). The purification of the dry crude performed by column chromatography on silica gel provides the amide **5n** (174 mg, 0.80 mmol, 80%).

Flash column chromatography was performed using a mixture of hexane/ethyl acetate 6:1 as an eluent to provide the corresponding amide product.

Brownish solid, mp 160–161 °C. **^1^H-NMR** (500 MHz, CDCl_3_): δ 2.32 (s, 3H, Me), 6.94 (t, 1H, ^3^*J* = 7.8 Hz, CH_Ar_), 7.01–7.08 (m, 1H, Thiophene), 7.21 (t, 1H, ^3^*J* = 7.8 Hz, CH_Ar_), 7.39 (d, 1H, ^3^*J* = 8.1 Hz, CH_Ar_), 7.47 (s, 1H, CH_Ar_), 7.51 (d, 1H, ^3^*J* = 4.8 Hz, Thiophene), 7.64 (d, 1H, ^3^*J* = 3.6 Hz, Thiophene).

**^13^C{^1^H}-NMR** (126 MHz, CDCl_3_): δ 21.4, 117.4, 121.0, 125.4, 127.8, 128.4, 128.8, 130.7, 137.5, 138.9, 139.4, 160.1.

HRMS (TOF MS ES+): Calcd for C_12_H_12_NOS (M + H) 240.1394. Found 218.0640.

***3,4-difluoro-N-isopropylbenzamide* 5o.** The title compound was prepared starting with an appropriate 2-bromo-2,2-difluoroacetamide **1k** (216 mg, 1.0 mmol, 1.0 equiv.), KF (116 mg, 2.0 mmol, 2.0 equiv.), MgCl_2_ (95 mg, 1.0 mmol, 1.0 equiv.), appropriate aryl boronic acid **2g** (205 mg, 1.3 mmol, 1.3 equiv.), the **L1** (130 mg, 0.2 mmol, 0.2 equiv.), CuBr_2_ (22.3 mg, 0.1 mmol, 0.1 equiv.), and hexafluoropropanol (0.12 mmol/mL). The purification of the dry crude performed by column chromatography on silica gel provides the amide **5o** (238 mg, 0.90 mmol, 90%).

Alternatively, the title compound was prepared starting with an appropriate 2-bromo-2,2-difluoroacetamide **1k** (216 mg, 1.0 mmol, 1.0 equiv.), KF (116 mg, 2.0 mmol, 2.0 equiv.), MgCl_2_ (95 mg, 1.0 mmol, 1.0 equiv.), appropriate trialkoxysilane **3g** (386 mg, 1.4 mmol, 1.4 equiv.), the **L1** (130 mg, 0.2 mmol, 0.2 equiv.), CuBr_2_ (22.3 mg, 0.1 mmol, 0.1 equiv.), and hexafluoropropanol (0.12 mmol/mL). The purification of the dry crude performed by column chromatography on silica gel provides the amide **5o** (233 mg, 0.88 mmol, 88%).

Flash column chromatography was performed using a mixture of hexane/ethyl acetate 8:1 as an eluent to provide the corresponding amide product.

White solid, mp 105–106 °C. **^1^H-NMR** (500 MHz, CDCl_3_): δ 1.14 (d, 6H, ^3^*J* = 6.2 Hz, Me), 4.08 (m, 1H, CH), 7.49–7.54 (m, 1H, CH_Ar_), 7.73–7374 (m, 1H, CH_Ar_), 7.87–7.91 (m, 1H, CH_Ar_), 8.32 (d, 1H, ^3^*J* = 6.8 Hz, NH).

**^13^C{^1^H}-NMR** (126 MHz, CDCl_3_): δ 22.2, 41.3, 116.6 (d, *J_CF_* = 18.3 Hz), 117.3 (d, *J_CF_* = 17.5 Hz), 124.7 (m), 132.2 (m), 149.1 (dd, ^1^*J_CF_* = 247.3 Hz, ^1^*J_CF_* = 12.3 Hz), 151.2 (dd, ^1^*J_CF_* = 250.2 Hz, ^1^*J_CF_* = 12.3 Hz), 163.0.

HRMS (TOF MS ES+): Calcd for C_13_H_19_NOCl (M + H) 240.1157. Found 240.1155.

***4-chloro-N,N-diisopropylbenzamide 5p.*** The title compound was prepared starting with an appropriate 2-bromo-2,2-difluoroacetamide **1l** (258 mg, 1.0 mmol, 1.0 equiv.), KF (116 mg, 2.0 mmol, 2.0 equiv.), MgCl_2_ (95 mg, 1.0 mmol, 1.0 equiv.), appropriate aryl boronic acid **2j** (203 mg, 1.3 mmol, 1.3 equiv.), the **L1** (130 mg, 0.2 mmol, 0.2 equiv.), CuBr_2_ (22.3 mg, 0.1 mmol, 0.1 equiv.), and hexafluoropropanol (0.12 mmol/mL). The purification of the dry crude performed by column chromatography on silica gel provides the amide **5p** (263 mg, 0.91 mmol, 91%).

Alternatively, the title compound was prepared starting with an appropriate 2-bromo-2,2-difluoroacetamide **1l** (258 mg, 1.0 mmol, 1.0 equiv.), KF (116 mg, 2.0 mmol, 2.0 equiv.), MgCl_2_ (95 mg, 1.0 mmol, 1.0 equiv.), appropriate trialkoxysilane **3h** (326 mg, 1.4 mmol, 1.4 equiv.), the **L1** (130 mg, 0.2 mmol, 0.2 equiv.), CuBr_2_ (22.3 mg, 0.1 mmol, 0.1 equiv.), and hexafluoropropanol (0.12 mmol/mL). The purification of the dry crude performed by column chromatography on silica gel provides the amide **5p** (266 mg, 0.92 mmol, 92%).

Flash column chromatography was performed using a mixture of hexane/ethyl acetate 8:1 as an eluent to provide the corresponding amide product.

White solid, mp 129–130 °C. **^1^H-NMR** (500 MHz, CDCl_3_): δ 1.15 (s, 6H, Me), 1.48 (s, 6H, Me), 3.58 (m, 2H, 2xCH), 7.24 (dt, 2H, ^3^*J* = 8.5 Hz, ^4^*J* = 2.0 Hz, CH_Ar_), 7.34 (dt, 2H, ^3^*J* = 8.3 Hz, ^4^*J* = 1.7 Hz, CH_Ar_).

**^13^C{^1^H}-NMR** (126 MHz, CDCl_3_): δ 20.7, 127.1, 128.7, 134.6, 137.2, 169.9.

HRMS (TOF MS ES+): Calcd for C_13_H_19_NOCl (M + H) 240.1157. Found 240.1155.

***2,3,4-trifluoro-N,N-diisopropylbenzamide 5q.*** Alternatively, the title compound was prepared starting with an appropriate 2-bromo-2,2-difluoroacetamide **1l** (258 mg, 1.0 mmol, 1.0 equiv.), KF (116 mg, 2.0 mmol, 2.0 equiv.), MgCl_2_ (95 mg, 1.0 mmol, 1.0 equiv.), appropriate aryl boronic acid **2h** (229 mg, 1.3 mmol, 1.3 equiv.), the **L1** (130 mg, 0.2 mmol, 0.2 equiv.), CuBr_2_ (22.3 mg, 0.1 mmol, 0.1 equiv.), and hexafluoropropanol (0.12 mmol/mL). The purification of the dry crude performed by column chromatography on silica gel provides the amide **5q** (215 mg, 0.83 mmol, 83%).

Flash column chromatography was performed using a mixture of hexane/ethyl acetate 11:1 as an eluent to provide the corresponding amide product.

White solid, mp 48–50 °C. **^1^H-NMR** (500 MHz, CDCl_3_): δ 1.10 (m, 6H, 2xMe), 1.50 (s, 3H, Me), 1.52 (s, 3H, Me), 3.50–3.53 (m, 1H, NCH), 3.65–3.68 53 (m, 1H, NCH), 6.97–7.00 (m, 2H, CH_Ar_).

**^13^C{^1^H}-NMR** (126 MHz, CDCl_3_): δ 20.5 (m), 46.2, 51.3, 112.9 (dd, *J_CF_* = 18.0 Hz, *J_CF_* = 3.3 Hz), 121.3 (m), 124.3 (dd, *J_CF_* = 16.4 Hz, *J_CF_* = 2.2 Hz), 139.7 (dt, ^1^*J_CF_* = 253.7 Hz, *J_CF_* = 15.2 Hz), 147.2 (ddd, ^1^*J_CF_* = 249.8 Hz, *J_CF_* = 10.9 Hz, *J_CF_* = 3.1 Hz), 151.2 (ddd, ^1^*J_CF_* = 251.3 Hz, *J_CF_* = 9.8 Hz, *J_CF_* = 2.4 Hz), 163.5.

HRMS (TOF MS ES+): Calcd for C_13_H_17_NOF_3_ (M + H) 260.1262. Found 260.1262.

***N-cyclohexyl-2-fluorobenzamide 5r.*** The title compound was prepared starting with an appropriate 2-bromo-2,2-difluoroacetamide **1p** (258 mg, 1.0 mmol, 1.0 equiv.), KF (116 mg, 2.0 mmol, 2.0 equiv.), MgCl_2_ (95 mg, 1.0 mmol, 1.0 equiv.), appropriate aryl boronic acid **2i** (203 mg, 1.3 mmol, 1.3 equiv.), the **L1** (130 mg, 0.2 mmol, 0.2 equiv.), CuBr_2_ (22.3 mg, 0.1 mmol, 0.1 equiv.), and hexafluoropropanol (0.12 mmol/mL). The purification of the dry crude performed by column chromatography on silica gel provides the amide **5r** (188 mg, 0.85 mmol, 85%).

Alternatively, the title compound was prepared starting with an appropriate 2-bromo-2,2-difluoroacetamide **1p** (258 mg, 1.0 mmol, 1.0 equiv.), KF (116 mg, 2.0 mmol, 2.0 equiv.), MgCl_2_ (95 mg, 1.0 mmol, 1.0 equiv.), appropriate potassium trifluoro(2-fluorophenyl)borate (263 mg, 1.3 mmol, 1.3 equiv.), the **L1** (130 mg, 0.2 mmol, 0.2 equiv.), CuBr_2_ (22.3 mg, 0.1 mmol, 0.1 equiv.), and hexafluoropropanol (0.12 mmol/mL). The purification of the dry crude performed by column chromatography on silica gel provides the amide **5r** (192 mg, 0.87 mmol, 87%).

Flash column chromatography was performed using a mixture of hexane/ethyl acetate 10:1 as an eluent to provide the corresponding amide product.

Colorless solid, mp 45–46 °C. **^1^H-NMR** (500 MHz, CDCl_3_): δ 1.19–1.29 (m, 3H, Cy), 1.36–1.46 (m, 2H, Cy), 1.58–1.62 (m, 1H, Cy), 1.69–1.73 (m, 2H, Cy), 1.98–2.01 (m, 2H, Cy), 3.98–4.00 (m, 1H, Cy), 6.61 (s, 1H, NH), 7.04–7.08 (m, 1H, CH_Ar_), 7.19–7.22 (m, 1H, CH_Ar_), 7.38–7.42 (m, 1H, CH_Ar_), 8.03 (dd, 1H, ^3^*J* = 7.9 Hz, ^4^*J* = 1.8 Hz, CH_Ar_).

**^13^C{^1^H}-NMR** (126 MHz, CDCl_3_): δ 24.7, 25.5, 32.9, 48.5, 115.8 (d, *J_CF_* = 23.6 Hz), 121.5 (d, *J_CF_* = 11.0 Hz), 124.6 (d, *J_CF_* = 2.7 Hz), 131.9, 133.0 (d, *J_CF_* = 9.0 Hz), 160.4 (d, ^1^*J_CF_* = 246.0 Hz, CF), 162.1 (d, *J_CF_* = 2.4 Hz).

HRMS (TOF MS ES+): Calcd for C_13_H_17_NOF (M + H) 222.1294. Found 222.1294.

***N-cyclohexyl-N-methyl-[1,1′-biphenyl]-4-carboxamide 5s.*** The title compound was prepared starting with an appropriate 2-bromo-2,2-difluoroacetamide **1o** (270 mg, 1.0 mmol, 1.0 equiv.), KF (116 mg, 2.0 mmol, 2.0 equiv.), MgCl_2_ (95 mg, 1.0 mmol, 1.0 equiv.), appropriate aryl boronic acid **2e** (257 mg, 1.3 mmol, 1.3 equiv.), the **L1** (130 mg, 0.2 mmol, 0.2 equiv.), CuBr_2_ (22.3 mg, 0.1 mmol, 0.1 equiv.), and hexafluoropropanol (0.12 mmol/mL). The purification of the dry crude performed by column chromatography on silica gel provides the amide **5s** (255 mg, 0.87 mmol, 87%).

Alternatively, the title compound was prepared starting with an appropriate 2-bromo-2,2-difluoroacetamide **1o** (270 mg, 1.0 mmol, 1.0 equiv.), KF (116 mg, 2.0 mmol, 2.0 equiv.), MgCl_2_ (95 mg, 1.0 mmol, 1.0 equiv.), appropriate trialkoxysilane **3e** (442 mg, 1.4 mmol, 1.4 equiv.), the **L1** (130 mg, 0.2 mmol, 0.2 equiv.), CuBr_2_ (22.3 mg, 0.1 mmol, 0.1 equiv.), and hexafluoropropanol (0.12 mmol/mL). The purification of the dry crude performed by column chromatography on silica gel provides the amide **5s** (243 mg, 0.83 mmol, 83%).

Flash column chromatography was performed using a mixture of hexane/ethyl acetate 8:1 as an eluent to provide the corresponding amide product.

Colorless solid, mp 105 - 106 °C. **^1^H-NMR** (500 MHz, CDCl_3_): δ 1.07–1.09 (m, 2H, Cy), 1.47–1.56 (m, 4H, Cy), 1.72–1.82 (m, 4H, Cy), 2.83, 3.00 (s, 3H, Me cis/trans), 3.54, 4.51 (s, 1H, Cy cis/trans), 7.33–7.36 (m, 1H, CH_Ar_), 7.42–7.45 (m, 4H, CH_Ar_), 7.60–7.61 (m, 4H, CH_Ar_).

**^13^C{^1^H}-NMR** (126 MHz, CDCl_3_): δ 25.0, 25.3, 25.4, 27.4, 29.4, 29.5, 30.7, 31.9, 52.7, 58.1, 126.5 126.7, 126.9, 127.1, 127.2, 127.5, 128.7, 135.8, 140.1, 141.8, 171.4.

HRMS (TOF MS ES+): Calcd for C_20_H_24_NO (M + H) 294.1856. Found 294.1858.

***N-benzyl-3-methoxybenzamide 5t.*** The title compound was prepared starting with an appropriate 2-bromo-2,2-difluoroacetamide **1u** (264 mg, 1.0 mmol, 1.0 equiv.), KF (116 mg, 2.0 mmol, 2.0 equiv.), MgCl_2_ (95 mg, 1.0 mmol, 1.0 equiv.), appropriate aryl boronic acid **2w** (198 mg, 1.3 mmol, 1.3 equiv.), the **L1** (130 mg, 0.2 mmol, 0.2 equiv.), CuBr_2_ (22.3 mg, 0.1 mmol, 0.1 equiv.), and hexafluoropropanol (0.12 mmol/mL). The purification of the dry crude performed by column chromatography on silica gel provides the amide **5t** (217 mg, 0.90 mmol, 90%).

Alternatively, the title compound was prepared starting with an appropriate 2-bromo-2,2-difluoroacetamide **1u** (264 mg, 1.0 mmol, 1.0 equiv.), KF (116 mg, 2.0 mmol, 2.0 equiv.), MgCl_2_ (95 mg, 1.0 mmol, 1.0 equiv.), appropriate trialkoxysilane **3j** (378 mg, 1.4 mmol, 1.4 equiv.), the **L1** (130 mg, 0.2 mmol, 0.2 equiv.), CuBr_2_ (22.3 mg, 0.1 mmol, 0.1 equiv.), and hexafluoropropanol (0.12 mmol/mL). The purification of the dry crude performed by column chromatography on silica gel provides the amide **5t** (210 mg, 0.87 mmol, 87%).

Alternatively, the title compound was prepared starting with an appropriate 2-bromo-2,2-difluoroacetamide **1u** (264 mg, 1.0 mmol, 1.0 equiv.), KF (116 mg, 2.0 mmol, 2.0 equiv.), MgCl_2_ (95 mg, 1.0 mmol, 1.0 equiv.), aryl sulphonium salt **4b** (410 mg, 1.6 mmol, 1.6 equiv.), the **L2** (202 mg, 0.25 mmol, 0.25 equiv.), [Ru(p-cymene)Cl_2_]_2_ (122 mg, 0.2 mmol, 0.2 equiv.), CuBr_2_ (67 mg, 0.3 mmol, 0.3 equiv.), and hexafluoropropanol (0.17 mmol/mL). The purification of the dry crude performed by column chromatography on silica gel provides the amide **5t** (202 mg, 0.84 mmol, 84%).

Alternatively, the title compound was prepared starting with an appropriate 2-bromo-2,2-difluoroacetamide **1u** (264 mg, 1.0 mmol, 1.0 equiv.), KF (116 mg, 2.0 mmol, 2.0 equiv.), MgCl_2_ (95 mg, 1.0 mmol, 1.0 equiv.), appropriate potassium trifluoro(3-methoxyphenyl)borate (278 mg, 1.3 mmol, 1.3 equiv.), the **L1** (130 mg, 0.2 mmol, 0.2 equiv.), CuBr_2_ (22.3 mg, 0.1 mmol, 0.1 equiv.), and hexafluoropropanol (0.12 mmol/mL). The purification of the dry crude performed by column chromatography on silica gel provides the amide **5t** (193 mg, 0.80 mmol, 80%).

Alternatively, the title compound was prepared starting with an appropriate 2-bromo-2,2-difluoroacetamide **1u** (264 mg, 1.0 mmol, 1.0 equiv.), KF (116 mg, 2.0 mmol, 2.0 equiv.), MgCl_2_ (95 mg, 1.0 mmol, 1.0 equiv.), appropriate 2-(3-methoxyphenyl)-4,4,5,5-tetramethyl-1,3,2-dioxaborolane (304 mg, 1.3 mmol, 1.3 equiv.), the **L1** (130 mg, 0.2 mmol, 0.2 equiv.), CuBr_2_ (22.3 mg, 0.1 mmol, 0.1 equiv.), and hexafluoropropanol (0.12 mmol/mL). The purification of the dry crude performed by column chromatography on silica gel provides the amide **5t** (200 mg, 0.83 mmol, 83%).

Alternatively, the title compound was prepared starting with an appropriate N-benzyl-2,2-difluoro-2-iodoacetamide (311 mg, 1.0 mmol, 1.0 equiv.), KF (116 mg, 2.0 mmol, 2.0 equiv.), MgCl_2_ (95 mg, 1.0 mmol, 1.0 equiv.), appropriate aryl boronic acid **2w** (198 mg, 1.3 mmol, 1.3 equiv.), the **L1** (130 mg, 0.2 mmol, 0.2 equiv.), CuBr_2_ (22.3 mg, 0.1 mmol, 0.1 equiv.), and hexafluoropropanol (0.12 mmol/mL). The purification of the dry crude performed by column chromatography on silica gel provides the amide **5t** (210 mg, 0.87 mmol, 87%).

Flash column chromatography was performed using a mixture of hexane/ethyl acetate 7:1 as an eluent to provide the corresponding amide product.

White solid, mp 77–78 °C. **^1^H-NMR** (500 MHz, CDCl_3_): δ 2.36 (s, 3H, Me), 4.60 (d, 2H, ^3^*J* = 5.6 Hz, CH_2_), 6.78 (br s, 1H, NH), 7.26–7.33 (m, 7H, CH_Ar_), 7.57 (d, 1H, ^3^*J* = 7.2 Hz, CH_Ar_), 7.63 (s, 1H, CH_Ar_).

**^13^C{^1^H}-NMR** (126 MHz, CDCl_3_): δ 21.2, 43.9, 123.9, 127.4, 127.7, 127.8, 128.3, 128.6, 132.1, 134.2, 138.3, 167.6.

HRMS (TOF MS ES+): Calcd for C_15_H_16_NO (M + H) 226.1234. Found 226.1232.

***N-cyclohexyl-N-phenyl-4-(trifluoromethyl)benzamide 5u.*** The title compound was prepared starting with an appropriate 2-bromo-2,2-difluoroacetamide **1n** (332 mg, 1.0 mmol, 1.0 equiv.), KF (116 mg, 2.0 mmol, 2.0 equiv.), MgCl_2_ (95 mg, 1.0 mmol, 1.0 equiv.), appropriate aryl boronic acid **2n** (247 mg, 1.3 mmol, 1.3 equiv.), the **L1** (130 mg, 0.2 mmol, 0.2 equiv.), CuBr_2_ (22.3 mg, 0.1 mmol, 0.1 equiv.), and hexafluoropropanol (0.12 mmol/mL). The purification of the dry crude performed by column chromatography on silica gel provides the amide **5u** (312 mg, 0.90 mmol, 90%).

Alternatively, the title compound was prepared starting with an appropriate 2-bromo-2,2-difluoroacetamide **1n** (332 mg, 1.0 mmol, 1.0 equiv.), KF (116 mg, 2.0 mmol, 2.0 equiv.), MgCl_2_ (95 mg, 1.0 mmol, 1.0 equiv.), appropriate trialkoxysilane **3k** (372 mg, 1.4 mmol, 1.4 equiv.), the **L1** (130 mg, 0.2 mmol, 0.2 equiv.), CuBr_2_ (22.3 mg, 0.1 mmol, 0.1 equiv.), and hexafluoropropanol (0.12 mmol/mL). The purification of the dry crude performed by column chromatography on silica gel provides the amide **5u** (312 mg, 0.90 mmol, 90%).

Alternatively, the title compound was prepared starting with an appropriate 2-bromo-2,2-difluoroacetamide **1n** (332 mg, 1.0 mmol, 1.0 equiv.), KF (116 mg, 2.0 mmol, 2.0 equiv.), MgCl_2_ (95 mg, 1.0 mmol, 1.0 equiv.), aryl sulphonium salt **4a** (584 mg, 1.6 mmol, 1.6 equiv.), the **L2** (202 mg, 0.25 mmol, 0.25 equiv.), [Ru(p-cymene)Cl_2_]_2_ (122 mg, 0.2 mmol, 0.2 equiv.), CuBr_2_ (67 mg, 0.3 mmol, 0.3 equiv.), and hexafluoropropanol (0.12 mmol/mL). The purification of the dry crude performed by column chromatography on silica gel provides the amide **5u** (281 mg, 0.81 mmol, 81%).

Flash column chromatography was performed using a mixture of hexane/ethyl acetate 8:1 as an eluent to provide the corresponding amide product.

Colorless solid, mp 195–196 °C. **^1^H-NMR** (500 MHz, CDCl_3_): δ 0.97 (tq, 1H, ^3^*J* = 13.4 Hz, ^4^*J* = 2.9 Hz, Cy), 1.22 (dq, 2H, ^3^*J* = 12.9 Hz, ^4^*J* = 2.9 Hz, Cy), 1.45 (d, 2H, ^3^*J* = 12.4 Hz, Cy), 1.61 (d, 1H, ^3^*J* = 13.3 Hz, Cy), 1.78 (d, 2H, ^3^*J* = 13.3 Hz, Cy), 1.96 (d, 2H, ^3^*J* = 11.1 Hz, Cy), 4.72 (s, 1H, Cy), 7.00 (d, 2H, ^3^*J* = 6.9 Hz, CH_Ar_), 7.19–7.20 (m, 3H, CH_Ar_), 7.31 (d, 2H, ^3^*J* = 7.3 Hz, CH_Ar_), 7.36 (d, 2H, ^3^*J* = 6.9 Hz, CH_Ar_).

**^13^C{^1^H}-NMR** (126 MHz, CDCl_3_): δ 25.3, 25.8, 31.5, 55.3, 123.7 (q, ^1^*J_CF_* = 271.2 Hz, CF_3_), 124.6, 127.8, 128.3, 128.6, 130.4 (q, ^2^*J_CF_* = 28.0 Hz, *C*CF_3_), 130.6, 139.2, 140.8, 169.1.

HRMS (TOF MS ES+): Calcd for C_20_H_21_NOF_3_ (M + H) 348.1581. Found 348.1575.

***N-((1s,3s)-adamantan-1-yl)-4-(trifluoromethyl)benzamide 5v.*** The title compound was prepared starting with an appropriate 2-bromo-2,2-difluoroacetamide **1q** (308 mg, 1.0 mmol, 1.0 equiv.), KF (116 mg, 2.0 mmol, 2.0 equiv.), MgCl_2_ (95 mg, 1.0 mmol, 1.0 equiv.), appropriate aryl boronic acid **2n** (247 mg, 1.3 mmol, 1.3 equiv.), the **L1** (130 mg, 0.2 mmol, 0.2 equiv.), CuBr_2_ (22.3 mg, 0.1 mmol, 0.1 equiv.), and hexafluoropropanol (0.12 mmol/mL). The purification of the dry crude performed by column chromatography on silica gel provides the amide **5v** (281 mg, 0.87 mmol, 87%).

Alternatively, the title compound was prepared starting with an appropriate 2-bromo-2,2-difluoroacetamide **1q** (308 mg, 1.0 mmol, 1.0 equiv.), KF (116 mg, 2.0 mmol, 2.0 equiv.), MgCl_2_ (95 mg, 1.0 mmol, 1.0 equiv.), appropriate trialkoxysilane **3k** (372 mg, 1.4 mmol, 1.4 equiv.), the **L1** (130 mg, 0.2 mmol, 0.2 equiv.), CuBr_2_ (22.3 mg, 0.1 mmol, 0.1 equiv.), and hexafluoropropanol (0.12 mmol/mL). The purification of the dry crude performed by column chromatography on silica gel provides the amide **5v** (291 mg, 0.90 mmol, 90%).

Alternatively, the title compound was prepared starting with an appropriate 2-bromo-2,2-difluoroacetamide **1q** (308 mg, 1.0 mmol, 1.0 equiv.), KF (116 mg, 2.0 mmol, 2.0 equiv.), MgCl_2_ (95 mg, 1.0 mmol, 1.0 equiv.), appropriate potassium trifluoro(4-(trifluoromethyl)phenyl)borate (328 mg, 1.3 mmol, 1.3 equiv.), the **L1** (130 mg, 0.2 mmol, 0.2 equiv.), CuBr_2_ (22.3 mg, 0.1 mmol, 0.1 equiv.), and hexafluoropropanol (0.12 mmol/mL). The purification of the dry crude performed by column chromatography on silica gel provides the amide **5v** (271 mg, 0.84 mmol, 84%).

Flash column chromatography was performed using a mixture of hexane/ethyl acetate 12:1 as an eluent to provide the corresponding amide product.

White solid, mp 158–159 °C. **^1^H-NMR** (500 MHz, CDCl_3_): δ 1.70 (s, 6H, Adam), 2.11 (s, 9H, Adam), 5.91 (s, 1H, NH), 7.61 (d, 2H, ^3^*J* = 8.6 Hz, CH_Ar_), 7.78 (d, 2H, ^3^*J* = 7.9 Hz, CH_Ar_).

**^13^C{^1^H}-NMR** (126 MHz, CDCl_3_): δ 29.4, 36.2, 41.5, 52.6, 123.7 (q, ^1^*J_CF_* = 273.6 Hz, CF_3_), 125.4 (m), 127.2, 132.6 (q, ^2^*J*_CF_ = 31.1 Hz, CCF_3_), 139.3.

HRMS (TOF MS ES+): Calcd for C_18_H_21_NOF_3_ (M + H) 324.1583. Found 324.1575.

***3-chloro-N-cyclopropylbenzamide 5w.*** The title compound was prepared starting with an appropriate 2-bromo-2,2-difluoroacetamide **1r** (214 mg, 1.0 mmol, 1.0 equiv.), KF (116 mg, 2.0 mmol, 2.0 equiv.), MgCl_2_ (95 mg, 1.0 mmol, 1.0 equiv.), appropriate aryl boronic acid **2k** (203 mg, 1.3 mmol, 1.3 equiv.), the **L1** (130 mg, 0.2 mmol, 0.2 equiv.), CuBr_2_ (22.3 mg, 0.1 mmol, 0.1 equiv.), and hexafluoropropanol (0.12 mmol/mL). The purification of the dry crude performed by column chromatography on silica gel provides the amide **5w** (127 mg, 0.65 mmol, 65%).

Alternatively, the title compound was prepared starting with an appropriate 2-bromo-2,2-difluoroacetamide **1r** (214 mg, 1.0 mmol, 1.0 equiv.), KF (116 mg, 2.0 mmol, 2.0 equiv.), MgCl_2_ (95 mg, 1.0 mmol, 1.0 equiv.), appropriate trialkoxysilane **3i** (326 mg, 1.4 mmol, 1.4 equiv.), the **L1** (130 mg, 0.2 mmol, 0.2 equiv.), CuBr_2_ (22.3 mg, 0.1 mmol, 0.1 equiv.), and hexafluoropropanol (0.12 mmol/mL). The purification of the dry crude performed by column chromatography on silica gel provides the amide **5w** (125 mg, 0.64 mmol, 64%).

Alternatively, the title compound was prepared starting with an appropriate 2-bromo-2,2-difluoroacetamide **1r** (214 mg, 1.0 mmol, 1.0 equiv.), KF (116 mg, 2.0 mmol, 2.0 equiv.), MgCl_2_ (95 mg, 1.0 mmol, 1.0 equiv.), aryl sulphonium salt **4d** (516 mg, 1.6 mmol, 1.6 equiv.), the **L2** (202 mg, 0.25 mmol, 0.25 equiv.), [Ru(p-cymene)Cl_2_]_2_ (122 mg, 0.2 mmol, 0.2 equiv.), CuBr_2_ (67 mg, 0.3 mmol, 0.3 equiv.), and hexafluoropropanol (0.12 mmol/mL). The purification of the dry crude performed by column chromatography on silica gel provides the amide **5w** (113 mg, 0.58 mmol, 58%).

Flash column chromatography was performed using a mixture of hexane/ethyl acetate 7:1 as an eluent to provide the corresponding amide product.

Colorless solid, mp 142—143 °C. **^1^H-NMR** (500 MHz, CDCl_3_): δ 0.83–0.86 (m, 2H, CH_2_), 1.06–1.09 (m, 2H, CH_2_), 1.48- 1.53 (m, 1H, CH), 7.04 (d, 1H, ^3^*J* = 7.9 Hz, CH_Ar_), 7.20 (t, 1H, ^3^*J* = 8.7 Hz, CH_Ar_), 7.32 (d, 1H, ^3^*J* = 7.9 Hz, CH_Ar_), 7.63 (br. s, 1H, CH_Ar_), 7.74 (s, 1H, NH).

**^13^C{^1^H}-NMR** (126 MHz, CDCl_3_): δ 8.2, 15.7, 17.7, 19.9, 124.0, 129.9, 134.6, 139.2, 172.3.

HRMS (TOF MS ES+): Calcd for C_10_H_11_NOCl (M + H) 196.0532. Found 196.0529.

***[1,1′-biphenyl]-4-yl(pyrrolidin-1-yl)methanone 5x.*** The title compound was prepared starting with an appropriate 2-bromo-2,2-difluoroacetamide **1s** (228 mg, 1.0 mmol, 1.0 equiv.), KF (116 mg, 2.0 mmol, 2.0 equiv.), MgCl_2_ (95 mg, 1.0 mmol, 1.0 equiv.), appropriate aryl boronic acid **2e** (257 mg, 1.3 mmol, 1.3 equiv.), the **L1** (130 mg, 0.2 mmol, 0.2 equiv.), CuBr_2_ (22.3 mg, 0.1 mmol, 0.1 equiv.), and hexafluoropropanol (0.12 mmol/mL). The purification of the dry crude performed by column chromatography on silica gel provides the amide **5x** (326 mg, 0.91 mmol, 91%).

Alternatively, the title compound was prepared starting with an appropriate 2-bromo-2,2-difluoroacetamide **1s** (228 mg, 1.0 mmol, 1.0 equiv.), KF (116 mg, 2.0 mmol, 2.0 equiv.), MgCl_2_ (95 mg, 1.0 mmol, 1.0 equiv.), appropriate trialkoxysilane **3e** (442 mg, 1.4 mmol, 1.4 equiv.), the **L1** (130 mg, 0.2 mmol, 0.2 equiv.), CuBr_2_ (22.3 mg, 0.1 mmol, 0.1 equiv.), and hexafluoropropanol (0.12 mmol/mL). The purification of the dry crude performed by column chromatography on silica gel provides the amide **5x** (223 mg, 0.89 mmol, 89%).

Alternatively, the title compound was prepared starting with an appropriate 2-bromo-2,2-difluoroacetamide **1s** (228 mg, 1.0 mmol, 1.0 equiv.), KF (116 mg, 2.0 mmol, 2.0 equiv.), MgCl_2_ (95 mg, 1.0 mmol, 1.0 equiv.), aryl sulphonium salt **4e** (582 mg, 1.6 mmol, 1.6 equiv.), the **L2** (202 mg, 0.25 mmol, 0.25 equiv.), [Ru(p-cymene)Cl_2_]_2_ (122 mg, 0.2 mmol, 0.2 equiv.), CuBr_2_ (67 mg, 0.3 mmol, 0.3 equiv.), and hexafluoropropanol (0.12 mmol/mL). The purification of the dry crude performed by column chromatography on silica gel provides the amide **5x** (206 mg, 0.82 mmol, 82%).

Flash column chromatography was performed using a mixture of hexane/ethyl acetate 8:1 as an eluent to provide the corresponding amide product.

White solid, mp 139–140 °C. **^1^H-NMR** (500 MHz, CDCl_3_): δ 1.86 (m, 4H, Pyrr), 3.39 (s, 2H, Pyrr), 3.58 (s, 2H, Pyrr), 7.34 (t, 1H, ^3^*J* = 7.7 Hz, CH_Ar_), 7.43 (t, 2H, ^3^*J* = 7.7 Hz, CH_Ar_), 7.57 – 7.59 (m, 6H, CH_Ar_).

**^13^C{^1^H}-NMR** (126 MHz, CDCl_3_): δ 24.3, 26.3, 46.1, 49.5, 126.7, 127.0, 127.6, 128.7, 135.8, 140.1, 142.4, 169.3.

HRMS (TOF MS ES+): Calcd for C_17_H_18_NO (M + H) 252.1391. Found 252.1388.

***N-phenethyl-1-naphthamide 5y.*** The title compound was prepared starting with an appropriate 2-bromo-2,2-difluoroacetamide **1t** (278 mg, 1.0 mmol, 1.0 equiv.), KF (116 mg, 2.0 mmol, 2.0 equiv.), MgCl_2_ (95 mg, 1.0 mmol, 1.0 equiv.), appropriate aryl boronic acid **2d** (224 mg, 1.3 mmol, 1.3 equiv.), the **L1** (130 mg, 0.2 mmol, 0.2 equiv.), CuBr_2_ (22.3 mg, 0.1 mmol, 0.1 equiv.), and hexafluoropropanol (0.12 mmol/mL). The purification of the dry crude performed by column chromatography on silica gel provides the amide **5y** (228 mg, 0.83 mmol, 83%). The gram scale synthesis was performed on 10 mmol of the starting 2-bromo-2,2-difluoroacetamide **1t** and the amide **5y** was prepared in 78% yield (2.15 g, 7.8 mmol).

Alternatively, the title compound was prepared starting with an appropriate 2-bromo-2,2-difluoroacetamide **1t** (278 mg, 1.0 mmol, 1.0 equiv.), KF (116 mg, 2.0 mmol, 2.0 equiv.), MgCl_2_ (95 mg, 1.0 mmol, 1.0 equiv.), appropriate trialkoxysilane **3d** (347 mg, 1.4 mmol, 1.4 equiv.), the **L1** (130 mg, 0.2 mmol, 0.2 equiv.), CuBr_2_ (22.3 mg, 0.1 mmol, 0.1 equiv.), and hexafluoropropanol (0.12 mmol/mL). The purification of the dry crude performed by column chromatography on silica gel provides the amide **5y** (212 mg, 0.77 mmol, 77%). The gram scale synthesis was performed on 10 mmol of the starting 2-bromo-2,2-difluoroacetamide **1t** and the amide **5y** was prepared in 70% yield (1.93 g, 7 mmol).

Flash column chromatography was performed using a mixture of hexane/ethyl acetate 5:1 as an eluent to provide the corresponding amide product.

White solid, mp 117–118 °C. **^1^H-NMR** (500 MHz, CDCl_3_): δ 3.00 (t, 2H, ^3^*J* = 6.1 Hz, CH_2_), 3.80 (q, 2H, ^3^*J* = 7.2 Hz, CH_2_), 6.13 (s, 1H, NH), 7.27–7.28 (m, 3H, CH_Ar_), 7.33–7.37 (m, 2H, CH_Ar_), 7.40–7.43 (m, 1H, CH_Ar_), 7.48–7.54 (m, 3H, CH_Ar_), 7.86–7.88 (m, 1H, CH_Ar_), 7.89 (d, 1H, ^3^*J* = 8.0 Hz, CH_Ar_), 8.20–8.22 (m, 1H, CH_Ar_).

**^13^C{^1^H}-NMR** (126 MHz, CDCl_3_): δ 35.6, 41.0, 124.6, 124.85, 125.3, 126.3, 126.5, 127.0, 128.2, 128.7, 128.8, 130.0, 130.4, 133.6, 134.5, 138.7.

HRMS (TOF MS ES+): Calcd for C_19_H_18_NO (M + H) 276.1396. Found 276.1388.

***N-benzyl-3-chlorobenzamide 5z.*** The title compound was prepared starting with an appropriate 2-bromo-2,2-difluoroacetamide **1u** (264 mg, 1.0 mmol, 1.0 equiv.), KF (116 mg, 2.0 mmol, 2.0 equiv.), MgCl_2_ (95 mg, 1.0 mmol, 1.0 equiv.), appropriate aryl boronic acid **2k** (203 mg, 1.3 mmol, 1.3 equiv.), the **L1** (130 mg, 0.2 mmol, 0.2 equiv.), CuBr_2_ (22.3 mg, 0.1 mmol, 0.1 equiv.), and hexafluoropropanol (0.12 mmol/mL). The purification of the dry crude performed by column chromatography on silica gel provides the amide **5z** (209 mg, 0.85 mmol, 85%).

Alternatively, the title compound was prepared starting with an appropriate 2-bromo-2,2-difluoroacetamide **1u** (264 mg, 1.0 mmol, 1.0 equiv.), KF (116 mg, 2.0 mmol, 2.0 equiv.), MgCl_2_ (95 mg, 1.0 mmol, 1.0 equiv.), appropriate trialkoxysilane **3i** (372 mg, 1.4 mmol, 1.4 equiv.), the **L1** (130 mg, 0.2 mmol, 0.2 equiv.), CuBr_2_ (22.3 mg, 0.1 mmol, 0.1 equiv.), and hexafluoropropanol (0.12 mmol/mL). The purification of the dry crude performed by column chromatography on silica gel provides the amide **5z** (194 mg, 0.79 mmol, 79%).

Flash column chromatography was performed using a mixture of hexane/ethyl acetate 2:1 as an eluent to provide the corresponding amide product.

Yellowish solid, mp 93–94 °C. **^1^H-NMR** (500 MHz, CDCl_3_): δ 4.57 (d, 2H, ^3^*J* = 5.5 Hz, CH_2_), 6.77 (s, 1H, NH), 7.27–7.35 (m, 6H, CH_Ar_), 7.44 (dd, 1H, ^3^*J* = 8.0 Hz, ^4^*J* = 1.0 Hz, CH_Ar_), 7.64 (d, 1H, ^3^*J* = 8.0 Hz, CH_Ar_), 7.77 (d, 1H, ^4^*J* = 1.8 Hz, CH_Ar_).

**^13^C{^1^H}-NMR** (126 MHz, CDCl_3_): δ 44.1, 125.1, 127.3, 127.6, 127.8, 128.7, 129.8, 131.5, 134.7, 136.1, 137.8, 166.1.

HRMS (TOF MS ES+): Calcd for C_14_H_13_NOCl (M + H) 246.0686. Found 246.0686.

***N-(4-fluorobenzyl)-2-methylbenzamide 5aa.*** The title compound was prepared starting with an appropriate 2-bromo-2,2-difluoroacetamide **1v** (282 mg, 1.0 mmol, 1.0 equiv.), KF (116 mg, 2.0 mmol, 2.0 equiv.), MgCl_2_ (95 mg, 1.0 mmol, 1.0 equiv.), appropriate aryl boronic acid **2l** (203 mg, 1.3 mmol, 1.3 equiv.), the **L1** (130 mg, 0.2 mmol, 0.2 equiv.), CuBr_2_ (22.3 mg, 0.1 mmol, 0.1 equiv.), and hexafluoropropanol (0.12 mmol/mL). The purification of the dry crude performed by column chromatography on silica gel provides the amide **5aa** (189 mg, 0.72 mmol, 72%).

Alternatively, the title compound was prepared starting with an appropriate 2-bromo-2,2-difluoroacetamide **1v** (282 mg, 1.0 mmol, 1.0 equiv.), KF (116 mg, 2.0 mmol, 2.0 equiv.), MgCl_2_ (95 mg, 1.0 mmol, 1.0 equiv.), aryl sulphonium salt **4f** (516 mg, 1.6 mmol, 1.6 equiv.), the **L2** (202 mg, 0.25 mmol, 0.25 equiv.), [Ru(p-cymene)Cl_2_]_2_ (122 mg, 0.2 mmol, 0.2 equiv.), CuBr_2_ (67 mg, 0.3 mmol, 0.3 equiv.), and hexafluoropropanol (0.12 mmol/mL). The purification of the dry crude performed by column chromatography on silica gel provides the amide **5aa** (158 mg, 0.60 mmol, 60%).

Alternatively, the title compound was prepared starting with an appropriate 2-bromo-2,2-difluoroacetamide **1v** (282 mg, 1.0 mmol, 1.0 equiv.), KF (116 mg, 2.0 mmol, 2.0 equiv.), MgCl_2_ (95 mg, 1.0 mmol, 1.0 equiv.), appropriate potassium (2-chlorophenyl)trifluoroborate (283 mg, 1.3 mmol, 1.3 equiv.), the **L1** (130 mg, 0.2 mmol, 0.2 equiv.), CuBr_2_ (22.3 mg, 0.1 mmol, 0.1 equiv.), and hexafluoropropanol (0.12 mmol/mL). The purification of the dry crude performed by column chromatography on silica gel provides the amide **5aa** (181 mg, 0.69 mmol, 69%).

Flash column chromatography was performed using a mixture of hexane/ethyl acetate 4:1 as an eluent to provide the corresponding amide product.

Colorless solid, mp 119–120 °C. **^1^H-NMR** (500 MHz, CDCl_3_): δ 2.36 (s, 3H, Me), 4.46 (d, 2H, ^3^*J* = 5.7 Hz, CH_2_), 6.40 (s, 1H, NH), 6.97 (t, 2H, ^3^*J* = 8.7 Hz, CH_Ar_), 7.11–7.17 (m, 2H, CH_Ar_), 7.23–7.28 (m, 4H, CH_Ar_).

**^13^C{^1^H}-NMR** (126 MHz, CDCl_3_): δ 19.7, 42.9, 115.4 (d, *J_CF_* = 22.0 Hz), 125.6, 126.6, 129.3 (d, *J_CF_* = 8.9 Hz), 129.9, 130.9, 134.1 (d, *J_CF_* = 2.3 Hz), 136.0 (d, *J_CF_* = 2.7 Hz), 126.1 (d, ^1^*J_CF_* = 243.3 Hz), 169.9.

HRMS (TOF MS ES+): Calcd for C_15_H_15_NOF (M + H) 244.1143. Found 244.1138.

***N,N-dibenzyl-4-fluorobenzamide 5ab.*** The title compound was prepared starting with an appropriate 2-bromo-2,2-difluoroacetamide **1m** (354 mg, 1.0 mmol, 1.0 equiv.), KF (116 mg, 2.0 mmol, 2.0 equiv.), MgCl_2_ (95 mg, 1.0 mmol, 1.0 equiv.), appropriate aryl boronic acid **2f** (174 mg, 1.3 mmol, 1.3 equiv.), the **L1** (130 mg, 0.2 mmol, 0.2 equiv.), CuBr_2_ (22.3 mg, 0.1 mmol, 0.1 equiv.), and hexafluoropropanol (0.12 mmol/mL). The purification of the dry crude performed by column chromatography on silica gel provides the amide **5ab** (290 mg, 0.91 mmol, 91%). The gram scale synthesis was performed on 10 mmol of the starting 2-bromo-2,2-difluoroacetamide **1m** and the amide **5ab** was prepared in 83% yield (2.65 g, 8.3 mmol).

Alternatively, the title compound was prepared starting with an appropriate 2-bromo-2,2-difluoroacetamide **1m** (354 mg, 1.0 mmol, 1.0 equiv.), KF (116 mg, 2.0 mmol, 2.0 equiv.), MgCl_2_ (95 mg, 1.0 mmol, 1.0 equiv.), appropriate trialkoxysilane **3f** (361 mg, 1.4 mmol, 1.4 equiv.), the **L1** (130 mg, 0.2 mmol, 0.2 equiv.), CuBr_2_ (22.3 mg, 0.1 mmol, 0.1 equiv.), and hexafluoropropanol (0.12 mmol/mL). The purification of the dry crude performed by column chromatography on silica gel provides the amide **5ab** (287 mg, 0.90 mmol, 90%). The gram scale synthesis was performed on 10 mmol of the starting 2-bromo-2,2-difluoroacetamide **1m** and the amide **5ab** was prepared in 78% yield (2.45 g, 7.8 mmol).

Alternatively, the title compound was prepared starting with an appropriate 2-bromo-2,2-difluoroacetamide **1m** (354 mg, 1.0 mmol, 1.0 equiv.), KF (116 mg, 2.0 mmol, 2.0 equiv.), MgCl_2_ (95 mg, 1.0 mmol, 1.0 equiv.), aryl sulphonium salt **4g** (490 mg, 1.6 mmol, 1.6 equiv.), the **L2** (202 mg, 0.25 mmol, 0.25 equiv.), [Ru(p-cymene)Cl_2_]_2_ (122 mg, 0.2 mmol, 0.2 equiv.), CuBr_2_ (67 mg, 0.3 mmol, 0.3 equiv.), and hexafluoropropanol (0.12 mmol/mL). The purification of the dry crude performed by column chromatography on silica gel provides the amide **5ab** (281 mg, 0.88 mmol, 88%).

Alternatively, the title compound was prepared starting with an appropriate 2-bromo-2,2-difluoroacetamide **1m** (354 mg, 1.0 mmol, 1.0 equiv.), KF (116 mg, 2.0 mmol, 2.0 equiv.), MgCl_2_ (95 mg, 1.0 mmol, 1.0 equiv.), appropriate 2-(4-fluorophenyl)-4,4,5,5-tetramethyl-1,3,2-dioxaborolane (289 mg, 1.3 mmol, 1.3 equiv.), the **L1** (130 mg, 0.2 mmol, 0.2 equiv.), CuBr_2_ (22.3 mg, 0.1 mmol, 0.1 equiv.), and hexafluoropropanol (0.12 mmol/mL). The purification of the dry crude performed by column chromatography on silica gel provides the amide **5ab** (284 mg, 0.89 mmol, 89%).

Flash column chromatography was performed using a mixture of hexane/ethyl acetate 3:1 as an eluent to provide the corresponding amide product.

White solid, mp 86–87 °C. **^1^H-NMR** (500 MHz, CDCl_3_): δ 4.20 (s, 2H, CH_2_), 4.71 (s, 2H, CH_2_), 7.07 (t, 2H, ^3^*J* = 7.6 Hz, CH_Ar_), 7.15 (br. s, 2H, CH_Ar_), 7.30–7.33 (m, 4H, CH_Ar_), 7.36–7.39 (m, 4H, CH_Ar_), 8.50–7.53 (m, 2H, CH_Ar_).

**^13^C{^1^H}-NMR** (126 MHz, CDCl_3_): δ 47.2, 51.6, 115.6 (d, *J_CF_* = 22.2 Hz), 126.8 (m), 127.7 (m), 128.4 (m), 128.7 (m), 128.9, 129.0, 132.0 (m), 136.5 (d, *J_CF_* = 67.9 Hz), 163.3 (d, ^1^*J_CF_* = 247.5 Hz), 171.3.

HRMS (TOF MS ES+): Calcd for C_21_H_19_NOF (M + H) 320.1455. Found 320.1451.

***N,N-dibenzyl-4-((trifluoromethyl)thio)benzamide 5ac.*** The title compound was prepared starting with an appropriate 2-bromo-2,2-difluoroacetamide **1m** (354 mg, 1.0 mmol, 1.0 equiv.), KF (116 mg, 2.0 mmol, 2.0 equiv.), MgCl_2_ (95 mg, 1.0 mmol, 1.0 equiv.), appropriate aryl boronic acid **2s** (289 mg, 1.3 mmol, 1.3 equiv.), the **L1** (130 mg, 0.2 mmol, 0.2 equiv.), CuBr_2_ (22.3 mg, 0.1 mmol, 0.1 equiv.), and hexafluoropropanol (0.12 mmol/mL). The purification of the dry crude performed by column chromatography on silica gel provides the amide **5ac** (337 mg, 0.84 mmol, 84%).

Flash column chromatography was performed using a mixture of hexane/ethyl acetate 3:1 as an eluent to provide the corresponding amide product.

Colorless solid, mp 68–70 °C. **^1^H-NMR** (500 MHz, CDCl_3_): δ 4.39 (s, 2H, CH_2_), 4.74 (s, 2H, CH_2_), 7.13 (d, 2H, ^3^*J* = 6.9 Hz, CH_Ar_), 7.30–7.34 (m, 4H, CH_Ar_), 7.37–7.40 (m, 4H, CH_Ar_), 7.54 (d, 2H, ^3^*J* = 8.1 Hz, CH_Ar_), 7.67 (d, 2H, ^3^*J* = 8.1 Hz, CH_Ar_).

**^13^C{^1^H}-NMR** (126 MHz, CDCl_3_): δ 47.1, 51.5, 127.1 (q, ^1^*J_CF_* = 258.2 Hz, SCF_3_), 126.9, 127.7, 128.2, 128.4, 128.8, 129.0, 135.9, 136.2, 136.5, 138.6, 170.8.

HRMS (TOF MS ES+): Calcd for C_22_H_19_NOF_3_S (M + H) 402.1141. Found 402.1139.

***N,N-dibenzyl-2-(trifluoromethyl)benzamide 5ad.*** The title compound was prepared starting with an appropriate 2-bromo-2,2-difluoroacetamide **1m** (354 mg, 1.0 mmol, 1.0 equiv.), KF (116 mg, 2.0 mmol, 2.0 equiv.), MgCl_2_ (95 mg, 1.0 mmol, 1.0 equiv.), appropriate aryl boronic acid **2o** (247 mg, 1.3 mmol, 1.3 equiv.), the **L1** (130 mg, 0.2 mmol, 0.2 equiv.), CuBr_2_ (22.3 mg, 0.1 mmol, 0.1 equiv.), and hexafluoropropanol (0.12 mmol/mL). The purification of the dry crude performed by column chromatography on silica gel provides the amide **5ac** (247 mg, 0.67 mmol, 67%).

Alternatively, the title compound was prepared starting with an appropriate 2-bromo-2,2-difluoroacetamide **1m** (354 mg, 1.0 mmol, 1.0 equiv.), KF (116 mg, 2.0 mmol, 2.0 equiv.), MgCl_2_ (95 mg, 1.0 mmol, 1.0 equiv.), appropriate trialkoxysilane **3l** (372 mg, 1.4 mmol, 1.4 equiv.), the **L1** (130 mg, 0.2 mmol, 0.2 equiv.), CuBr_2_ (22.3 mg, 0.1 mmol, 0.1 equiv.), and hexafluoropropanol (0.12 mmol/mL). The purification of the dry crude performed by column chromatography on silica gel provides the amide **5ab** (229 mg, 0.62 mmol, 62%).

Flash column chromatography was performed using a mixture of hexane/ethyl acetate 4:1 as an eluent to provide the corresponding amide product.

Colorless solid, mp 135–136 °C. **^1^H-NMR** (500 MHz, CDCl_3_): δ 4.11 (t, 2H, ^3^*J* = 15.3 Hz, CH_2_), 4.25 (d, 1H, ^3^*J* = 15.8 Hz, CH_2_), 5.33 (d, 1H, ^3^*J* = 14.8 Hz, CH_2_), 7.11 (m, 2H, ^3^*J* = 7.1 Hz, CH_Ar_), 7.29–7.37 (m, 8H, CH_Ar_), 7.47–7.51 (m, 2H, CH_Ar_), 7.56 (t, 1H, ^3^*J* = 7.1 Hz, CH_Ar_), 7.71 (d, 1H, ^3^*J* = 7.8 Hz, CH_Ar_).

**^13^C{^1^H}-NMR** (126 MHz, CDCl_3_): δ 46.4, 51.1, 123.7 (q, ^1^*J_CF_* = 274.8 Hz, CF_3_), 126.6 (m), 127.3, 127.4, 127.7, 127.8, 128.5, 128.8, 129.1, 129.1, 132.1, 135.0, 135.5, 136.1, 169.2.

HRMS (TOF MS ES+): Calcd for C_22_H_19_NOF_3_ (M + H) 370.1422. Found 370.1419.

***N1,N1,N3,N3-tetrabenzylisophthalamide 5ae.*** The title compound was prepared starting with an appropriate 2-bromo-2,2-difluoroacetamide **1m** (885 mg, 2.5 mmol, 2.5 equiv.), KF (232 mg, 2.0 mmol, 2.0 equiv.), MgCl_2_ (190 mg, 2.0 mmol, 2.0 equiv.), appropriate aryl boronic acid **2v** (166 mg, 1.0 mmol, 1.0 equiv.), the **L1** (260 mg, 0.4 mmol, 0.4 equiv.), CuBr_2_ (44.6 mg, 0.2 mmol, 0.2 equiv.), and hexafluoropropanol (0.08 mmol/mL). The purification of the dry crude performed by column chromatography on silica gel provides the amide **5ae** (445 mg, 0.85 mmol, 85%).

Alternatively, the title compound was prepared starting with an appropriate 2-bromo-2,2-difluoroacetamide **1m** (885 mg, 2.5 mmol, 2.5 equiv.), KF (232 mg, 4.0 mmol, 4.0 equiv.), MgCl_2_ (190 mg, 2.0 mmol, 2.0 equiv.), appropriate trialkoxysilane **3q** (318 mg, 1.0 mmol, 1.0 equiv.), the **L1** (260 mg, 0.4 mmol, 0.4 equiv.), CuBr_2_ (44.6 mg, 0.2 mmol, 0.2 equiv.), and hexafluoropropanol (0.08 mmol/mL). The purification of the dry crude performed by column chromatography on silica gel provides the amide **5ae** (419 mg, 0.80 mmol, 80%).

Flash column chromatography was performed using a mixture of hexane/ethyl acetate 1:2 as an eluent to provide the corresponding amide product.

White solid, mp 172–173 °C. **^1^H-NMR** (500 MHz, CDCl_3_): δ 4.39 (s, 4H, 2xCH_2_), 4.71 (s, 4H, 2xCH_2_), 7.12 (d, 4H, ^3^*J* = 6.8 Hz, CH_Ar_), 7.28–7.37 (m, 16H, CH_Ar_), 7.53 (s, 4H, CH_Ar_).

**^13^C{^1^H}-NMR** (126 MHz, CDCl_3_): δ 46.9, 51.4, 126.8, 126.9, 127.6, 127.7, 128.4, 128.7, 128.9, 136.0, 136.6, 137.4, 171.3.

HRMS (TOF MS ES+): Calcd for C_36_H_33_N_2_O_2_ (M + H) 525.2540. Found 525.2539.

## 4. Conclusions

Summing up, basing on the mechanism assumption, we described three new mechanism-guided copper-catalyzed protocols for the direct arylation of 2-bromo-2,2-difluoroacetamides using aryl boronic acids, aryl trialloxysilanes, and aryl sulphonium salts as the aryl donors. The deployment of the scope of the reactions showcased the unique tolerance of the developed methodologies towards vast range of structural patterns and substituents on all coupling parts. These methods offer rapid entry to structurally diverse aromatic amides from simple and commercially availed precursors. Noteworthily, all methodologies were prone for scale-up to gram quantities.

## Figures and Tables

**Table 1:** Optimization of the reaction conditions for synthetic protocol (a).

Reaction (a) ^b^			
Entry	Reagent (Equiv)/Catalyst/Ligand/Additive	Solvent/Temperature/Time	Yield (%) 5a ^a^
1	boronic acid (1.5)/CuI (0.1)/KF (2.0)	DMF/100 °C/12 h	0
2	boronic acid (1.5)/CuBr_2_ (0.1)/KF (2.0)	DMF/100 °C/12 h	Trace
3	boronic acid (1.5)/CuI (0.1)/KF (2.0), MgCl_2_ (1.0)	(CF_3_)_2_CHOH/70 °C/12 h	12
4	boronic acid (1.5)/CuI (0.1)/L1 (0.2)/KF (2.0), MgCl_2_ (1.0)	(CF_3_)_2_CHOH/70 °C/12 h	38
5	boronic acid (1.5)/CuF_2_ (0.1)/L1 (0.2)/KF (2.0), MgCl_2_ (1.0)	(CF_3_)_2_CHOH/70 °C/12 h	49
6	boronic acid (1.5)/CuCl_2_ (0.1)/L1 (0.2)/KF (2.0), MgCl_2_ (1.0)	(CF_3_)_2_CHOH/70 °C/12 h	67
7	boronic acid (1.5)/CuBr_2_ (0.1)/L1 (0.2)/KF (2.0), MgCl_2_ (1.0)	(CF_3_)_2_CHOH/70 °C/12 h	88
**8**	**boronic acid (1.3)/CuBr_2_ (0.1)/L1 (0.2)/KF (2.0), MgCl_2_ (1.0)**	**(CF_3_)_2_CHOH/70 °C/8 h**	**87**
9	boronic acid (1.3)/CuBr_2_ (0.1)/L1 (0.2)/KF (2.0), MgCl_2_ (1.0)	DMF/70 °C/8 h	11
10	boronic acid (1.3)/CuBr_2_ (0.1)/KF (2.0), MgCl_2_ (1.0)	(CF_3_)_2_CHOH/70 °C/8 h	18
11	boronic acid (1.3)/L1 (0.2)/KF (2.0), MgCl_2_ (1.0)	(CF_3_)_2_CHOH/70 °C/8 h	0
12	boronic acid (1.3)/CuBr_2_ (0.1)/L1 (0.2)/KF (2.0)	(CF_3_)_2_CHOH/70 °C/8 h	27
13	boronic acid (1.3)/CuBr_2_ (0.1)/L1 (0.2)/KF (2.0), MgCl_2_ (1.0)/TEMPO (2.0)	CF_3_)_2_CHOH/70 °C/8 h	75
14	boronic acid (1.3)/CuBr_2_ (0.1)/L1 (0.2)/KF (2.0), MgCl_2_ (1.0)/TEMPO (3.0)	(CF_3_)_2_CHOH/70 °C/8 h	60
15	boronic acid (1.3)/CuBr_2_ (0.1)/L1 (0.2)/KF (2.0), MgCl_2_ (1.0)—*in dark*	(CF_3_)_2_CHOH/70 °C/8 h	84

^a^ Isolated yield. ^b^ All reactions were conducted in inert atmosphere.

**Table 2:** Optimization of the reaction conditions for synthetic protocol (b).

Reaction (b) ^b^			
Entry	Reagent (Equiv)/Catalyst/Ligand/Additive	Solvent/Temperature/Time	Yield (%) 5a ^a^
**1**	**aryl trialkoxysilane (1.4)/CuBr_2_ (0.1)/L1 (0.2)/KF (2.0), MgCl_2_ (1.0)**	**(CF_3_)_2_CHOH/70 °C/8 h**	**90**
2	aryl trialkoxysilane (1.4)/CuBr_2_ (0.1)/L1 (0.2)/KF (2.0), MgCl_2_ (1.0)	DMF/70 °C/8 h	22
3	aryl trialkoxysilane (1.4)/CuBr_2_ (0.1)/KF (2.0), MgCl_2_ (1.0)	(CF_3_)_2_CHOH/70 °C/8 h	21
4	aryl trialkoxysilane (1.4)/L1 (0.2)/KF (2.0), MgCl_2_ (1.0)	(CF_3_)_2_CHOH/70 °C/8 h	0
5	aryl trialkoxysilane (1.4)/CuBr_2_ (0.1)/L1 (0.2)/KF (2.0)	(CF_3_)_2_CHOH/70 °C/8 h	25
6	aryl trialkoxysilane (1.4)/CuBr_2_ (0.1)/L1 (0.2)/KF (2.0), MgCl_2_ (1.0)/TEMPO (2.0)	(CF_3_)_2_CHOH/70 °C/8 h	72
7	aryl trialkoxysilane (1.4)/CuBr_2_ (0.1)/L1 (0.2)/KF (2.0), MgCl_2_ (1.0)/TEMPO (3.0)	(CF_3_)_2_CHOH/70 °C/8 h	58
8	aryl trialkoxysilane (1.4)/CuBr_2_ (0.1)/L1 (0.2)/KF (2.0), MgCl_2_ (1.0)—*in dark*	(CF_3_)_2_CHOH/70 °C/8 h	91

^a^ Isolated yield. ^b^ All reactions were conducted at room temperature in inert atmosphere.

**Table 3:** Optimization of the reaction conditions for synthetic protocol (c).

Reaction (c) ^b^			
Entry	Reagent (Equiv)/Catalyst/Ligand/Additive	Solvent/Temperature/Time	Yield (%) 5a ^a^
1	sulphonium salt (1.4)/CuBr_2_ (0.1)/L1 (0.2)/KF (2.0), MgCl_2_ (1.0)	(CF_3_)_2_CHOH/70 °C/12 h	47
2	sulphonium salt (2)/CuCl_2_ (0.1), /L1 (0.2)/KF (2.0), MgCl_2_ (1.0)	(CF_3_)_2_CHOH/70 °C/12 h	Trace
3	sulphonium salt (2)/CuI (0.1), /L1 (0.2)/KF (2.0), MgCl_2_ (1.0)	(CF_3_)_2_CHOH/70 °C/12 h	Trace
4	sulphonium salt (1.6)/CuBr_2_ (0.3), Pd(OAc)_2_ (0.2)/L1 (0.25)/KF (2.0), MgCl_2_ (1.0)	(CF_3_)_2_CHOH/70 °C/12 h	17
5	sulphonium salt (1.6)/CuBr_2_ (0.3)/L2 (0.25)/KF (2.0), MgCl_2_ (1.0)	(CF_3_)_2_CHOH/70 °C/12 h	29
6	sulphonium salt (1.6)/CuBr_2_ (0.3), PdCl_2_ (0.2)/L2 (0.25)/KF (2.0), MgCl_2_ (1.0)	(CF_3_)_2_CHOH/70 °C/12 h	48
7	sulphonium salt (1.6)/CuBr_2_ (0.3), Pd(OAc)_2_ (0.2)/L2 (0.25)/KF (2.0), MgCl_2_ (1.0)	(CF_3_)_2_CHOH/70 °C/12 h	53
**8**	**sulphonium salt (1.6)/CuBr_2_ (0.3), [Ru(p-cymene)Cl_2_]_2_ (0.2)/L2 (0.25)/KF (2.0), MgCl_2_ (1.0)**	**(CF_3_)_2_CHOH/70 °C/11 h**	**84**
9	sulphonium salt (1.6)/CuBr_2_ (0.3), [Ru(p-cymene)Cl_2_]_2_ (0.2)/KF (2.0), MgCl_2_ (1.0)	(CF_3_)_2_CHOH/70 °C/11 h	27
10	sulphonium salt (1.6)/[Ru(p-cymene)Cl_2_]_2_ (0.2)/L2 (0.25)/KF (2.0), MgCl_2_ (1.0)	(CF_3_)_2_CHOH/70 °C/11 h	0
11	sulphonium salt (1.6)/CuBr_2_ (0.3), [Ru(p-cymene)Cl_2_]_2_ (0.2)/L2 (0.25)/KF (2.0)	(CF_3_)_2_CHOH/70 °C/11 h	18
12	sulphonium salt (1.6)/CuBr_2_ (0.3), [Ru(p-cymene)Cl_2_]_2_ (0.2)/L2 (0.25)/KF (2.0), MgCl_2_ (1.0)/TEMPO (2.0)	(CF_3_)_2_CHOH/70 °C/11 h	69
13	sulphonium salt (1.6)/CuBr_2_ (0.3), [Ru(p-cymene)Cl_2_]_2_ (0.2)/L2 (0.25)/KF (2.0), MgCl_2_ (1.0)/TEMPO (3.0)	(CF_3_)_2_CHOH/70 °C/11 h	55
14	sulphonium salt (1.6)/CuBr_2_ (0.3), [Ru(p-cymene)Cl_2_]_2_ (0.2)/L2 (0.25)/KF (2.0), MgCl_2_ (1.0)—*in dark*	(CF_3_)_2_CHOH/70 °C/11 h	86

^a^ Isolated yield. ^b^ All reactions were conducted at room temperature in inert atmosphere.

## Data Availability

Data is contained within the article or Supplementary Materials.

## Supplementary Materials

Scheme S1: List of 2-bromo-2,2-difluoroacetamides **1** used for preparation of amides **5**; Scheme S2: List of aryl boronic acids **2** used for preparation of amides **5**; Scheme S3: List of (aryl)trialkoxysilanes **3** used for preparation of amides **5**; Scheme S4: List of (aryl)dimethylsulfonium salts **4** used for preparation of amides **5**; Copies ^1^H and ^13^C-NMR spectra.

## Sample Availability

Samples of all compounds are available from the authors.
