# Supplementary material for: Cu-Catalyzed Arylation of Bromo-Difluoro-Acetamides by Aryl Boronic Acids, Aryl Trialkoxysilanes and Dimethyl-Aryl-Sulfonium Salts: New Entries to Aromatic Amides
Source: Molecules. 2021 May 16;26(10):2957. doi: 10.3390/molecules26102957 (PMC8156957; doi:10.3390/molecules26102957)
Supplement: Supplementary file 1 [file molecules-26-02957-s001.zip › molecules-1225113-supplementary.pdf]

Cu-catalyzed arylation of bromo-difluoro-acetamides by aryl boronic acids, aryl trialkoxysilanes and dimethyl-aryl-sulfonium salts: New entries to aromatic amides.

Satenik Mkrtchyan,[a]* Michał Jakubczyk,[a,b] Suneel Lanka,[a,c] Michael Pittelkow,[d] Viktor O. Iaroshenko.[a,e,f]*

a. Laboratory of Homogeneous Catalysis and Molecular Design at the Center of Molecular and Macromolecular Studies, Polish Academy of Sciences, Sienkiewicza 112, 90-363 Łodź, Poland.
b. Institute of Bioorganic Chemistry, Polish Academy of Sciences, Noskowskiego 12/14, 61-704 Poznań, Poland.
c. Institute of General and Ecological Chemistry, Lodz University of Technology, Zeromskiego 116, 909-24, Poland.
d. Department of Chemistry, University of Copenhagen, Universitetsparken 5, 2100 Copenhagen Ø, Denmark.
e. Dipartimento di Chimica e Biologia "A. Zambelli" Università di Salerno, Via Giovanni Paolo II, 84084, Fisciano (SA), Italy.
f. Department of Chemistry, University of Helsinki, A.I. Virtasen aukio 1, 00014 Helsinki, Finland.

Table of Contents

(A) List of reagents used ---- 3
(B) Copies ^{1}H and ^{13}C NMR spectra ---- 6

(A) List of reagents used.

Scope of 2-bromo-2,2-difluoroacetamides:

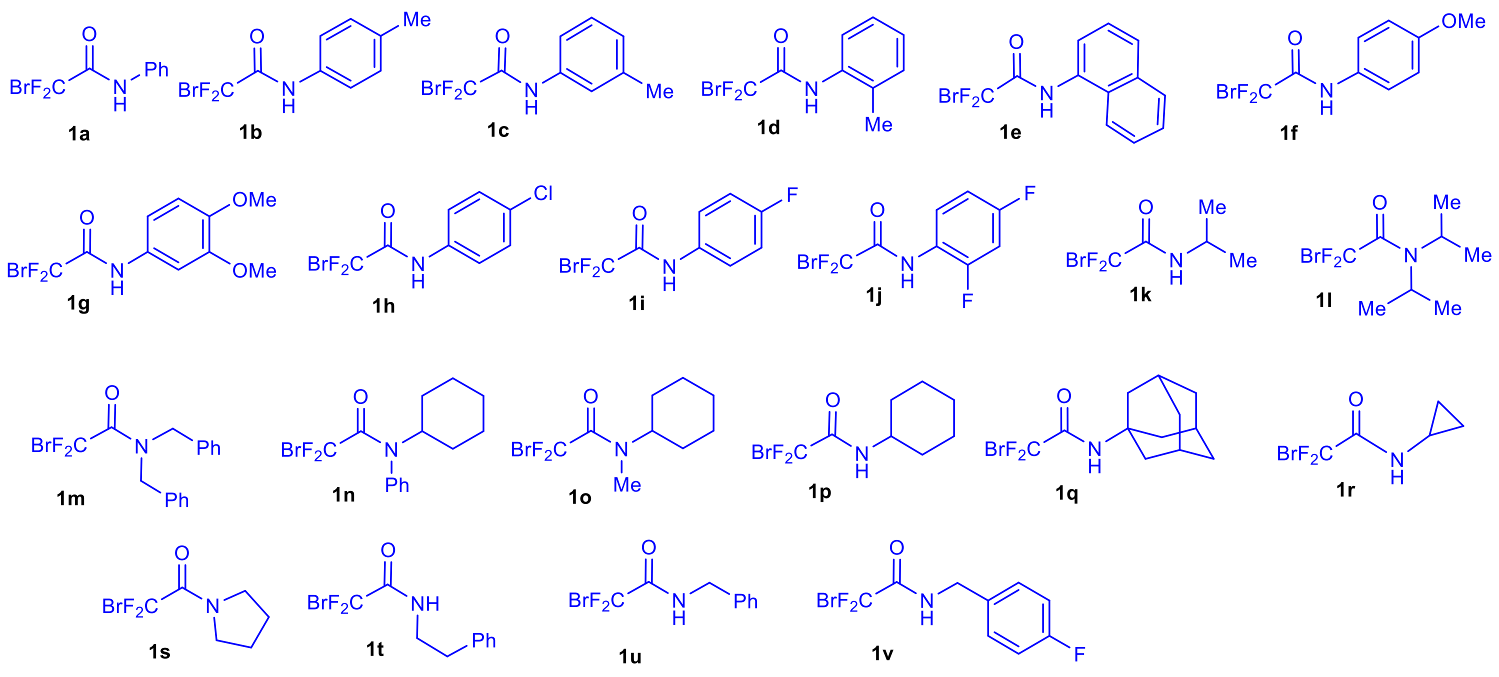

Scheme S1. List of 2-bromo-2,2-difluoroacetamides **1** used for preparation of amides **5**.

Scope of aryl boronic acids:

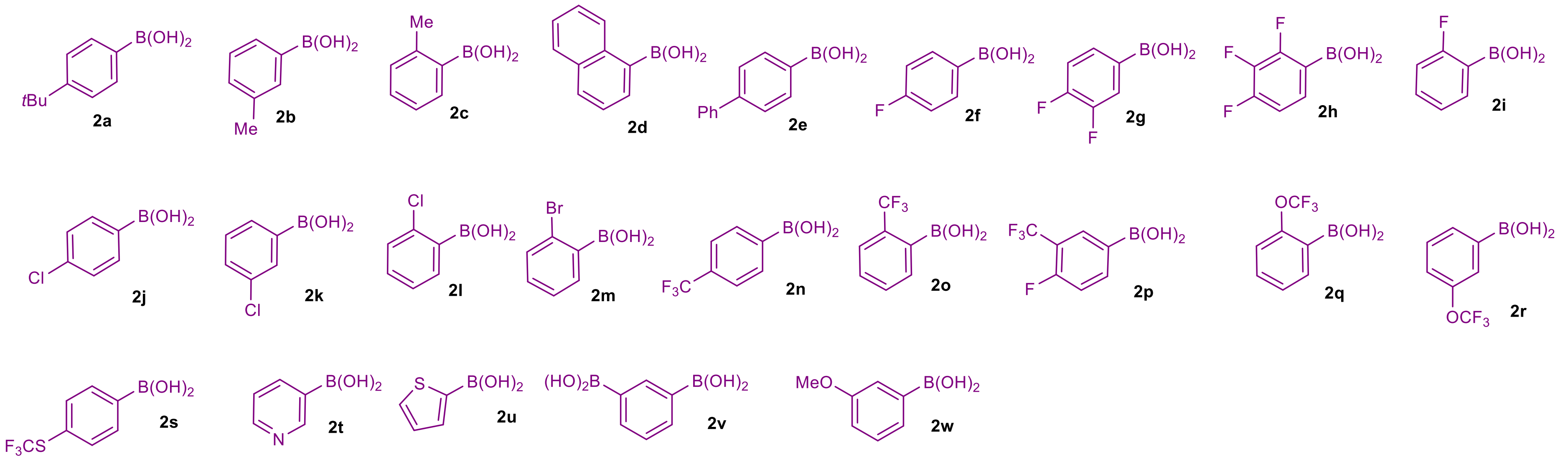

Scheme S2. List of aryl boronic acids **2** used for preparation of amides **5**.

Scope of (aryl)trialkoxysilanes:

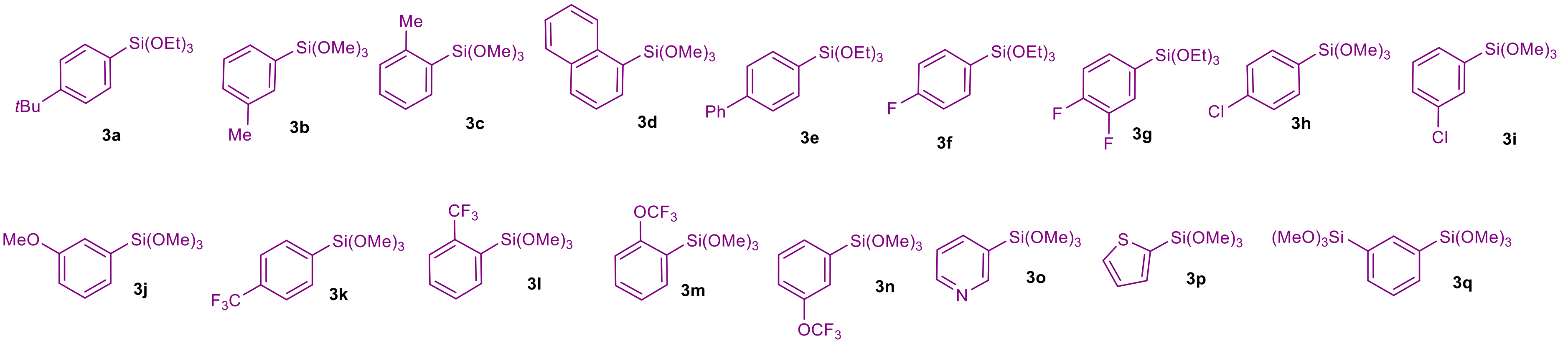

Scheme S3. List of (aryl)trialkoxysilanes **3** used for preparation of amides **5**.

Scope of dimethyl(aryl)sulfoniums:

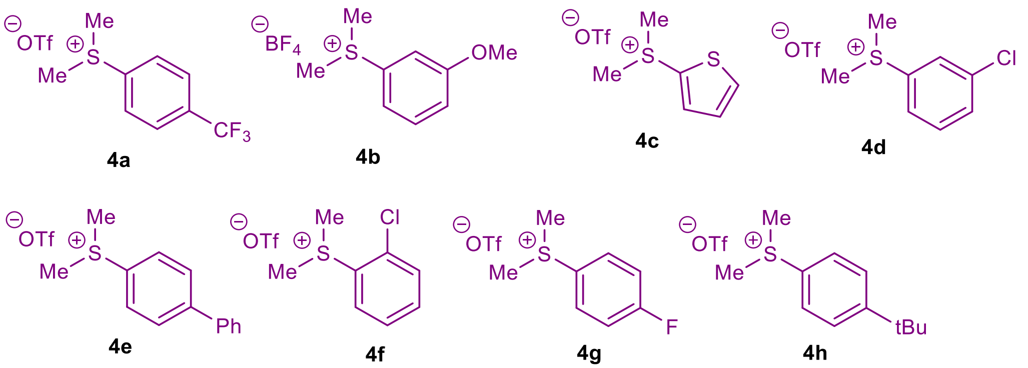

Scheme S4. List of (aryl)dimethylsulfonium salts **4** used for preparation of amides **5**.

(B) Copies ^{1}H and ^{13}C NMR spectra.

Compound 5a (NMR in DMSO-d_6)

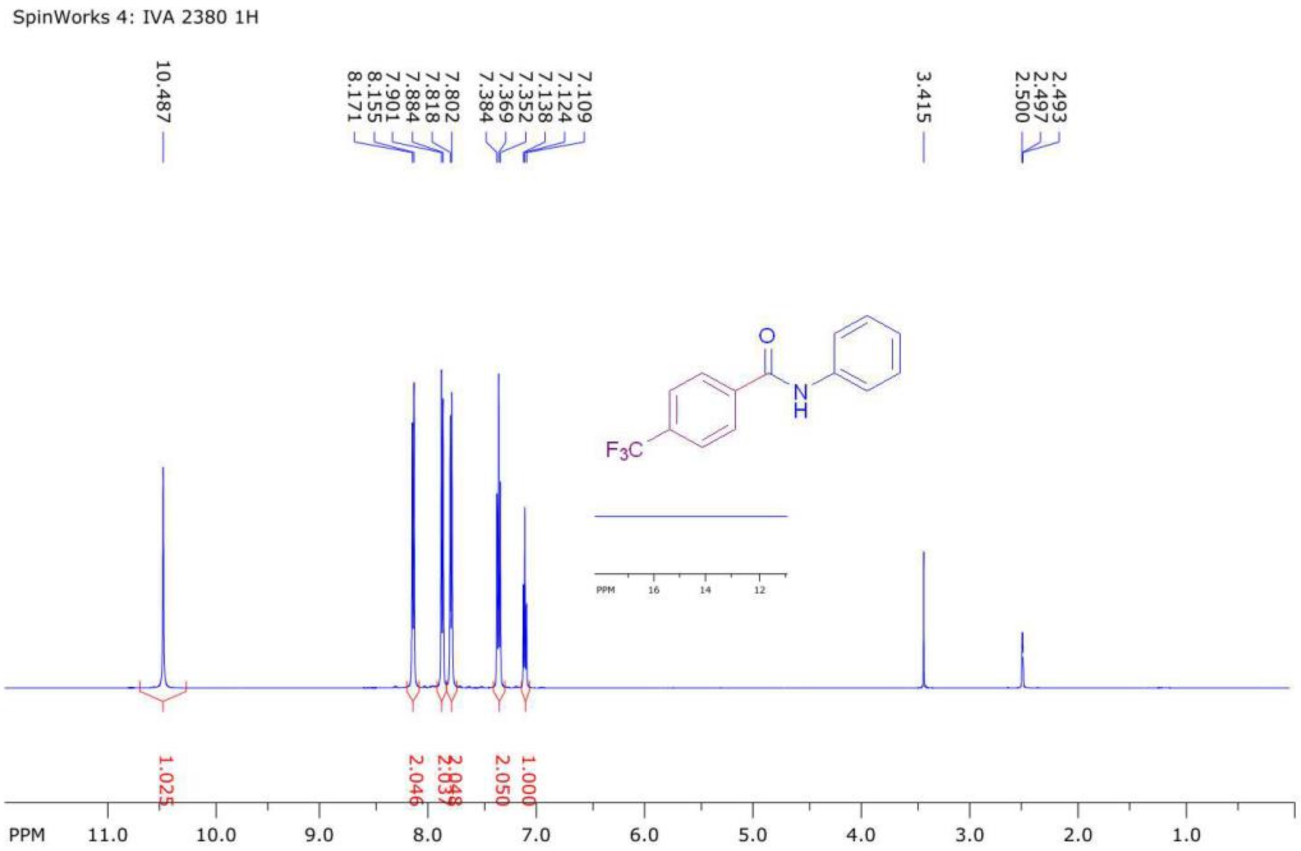

file: ...NAPO\NMR\500-2\mkr10207\9 2380\fid expt: <zg30>
transmitter freq.: 500.133001 MHz
time domain size: 65536 points
width: 12335.53 Hz = 24.6645 ppm = 0.188225 Hz/pt
number of scans: 24

freq. of 0 ppm: 500.130007 MHz
processed size: 65536 complex points
LB: 0.300 GF: 0.0000
Hz/cm: 239.069 ppm/cm: 0.47801

Compound 5a (NMR in DMSO-d_6)

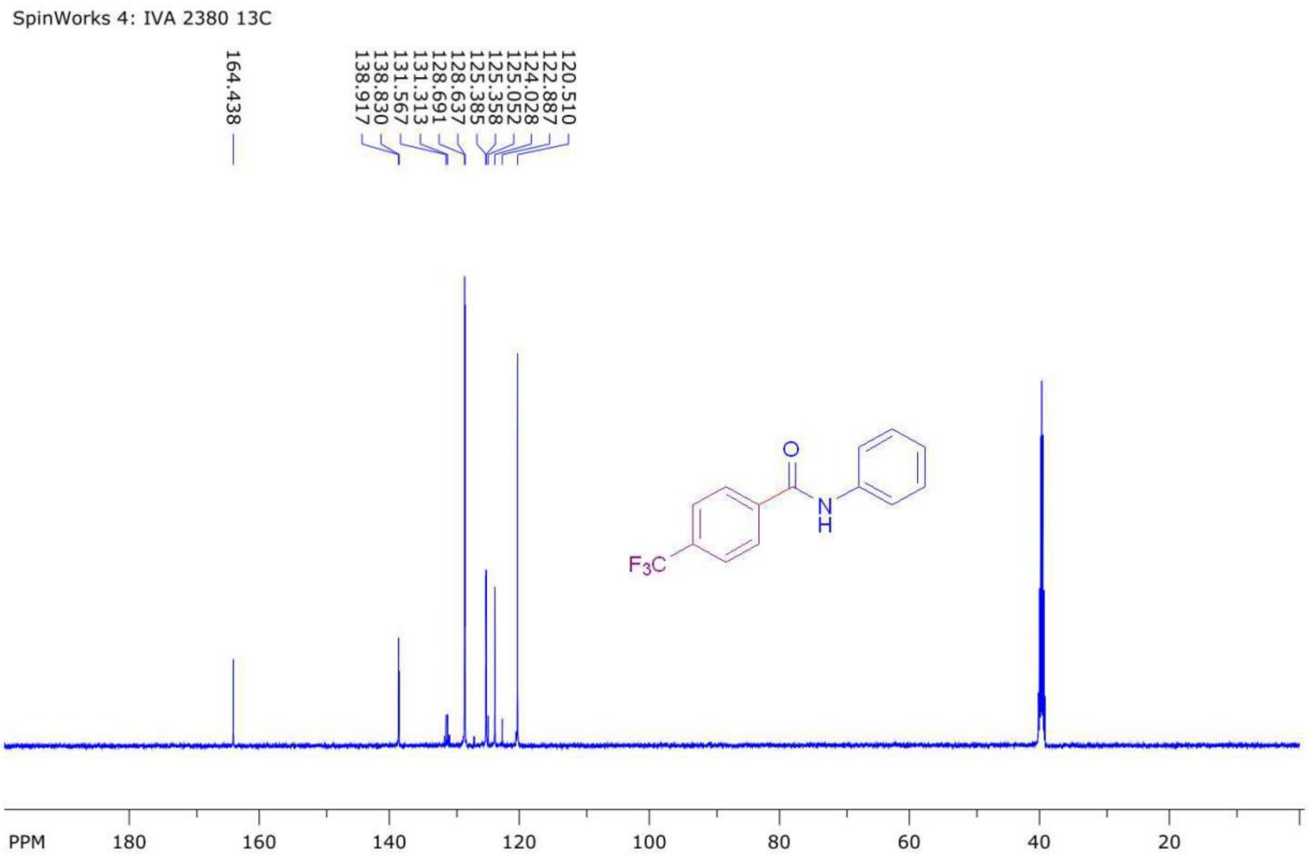

file: D:\NAPO\NMR\500-2\mkr10207\10\fid expt: <zgpg30>
transmitter freq.: 125.772879 MHz
time domain size: 65536 points
width: 36057.69 Hz = 286.6889 ppm = 0.550197 Hz/pt
number of scans: 512

freq. of 0 ppm: 125.757839 MHz
processed size: 32768 complex points
LB: 2.000 GF: 0.0000
Hz/cm: 1006.744 ppm/cm: 8.00446

Compound 5b (NMR in $CDCl_3$)

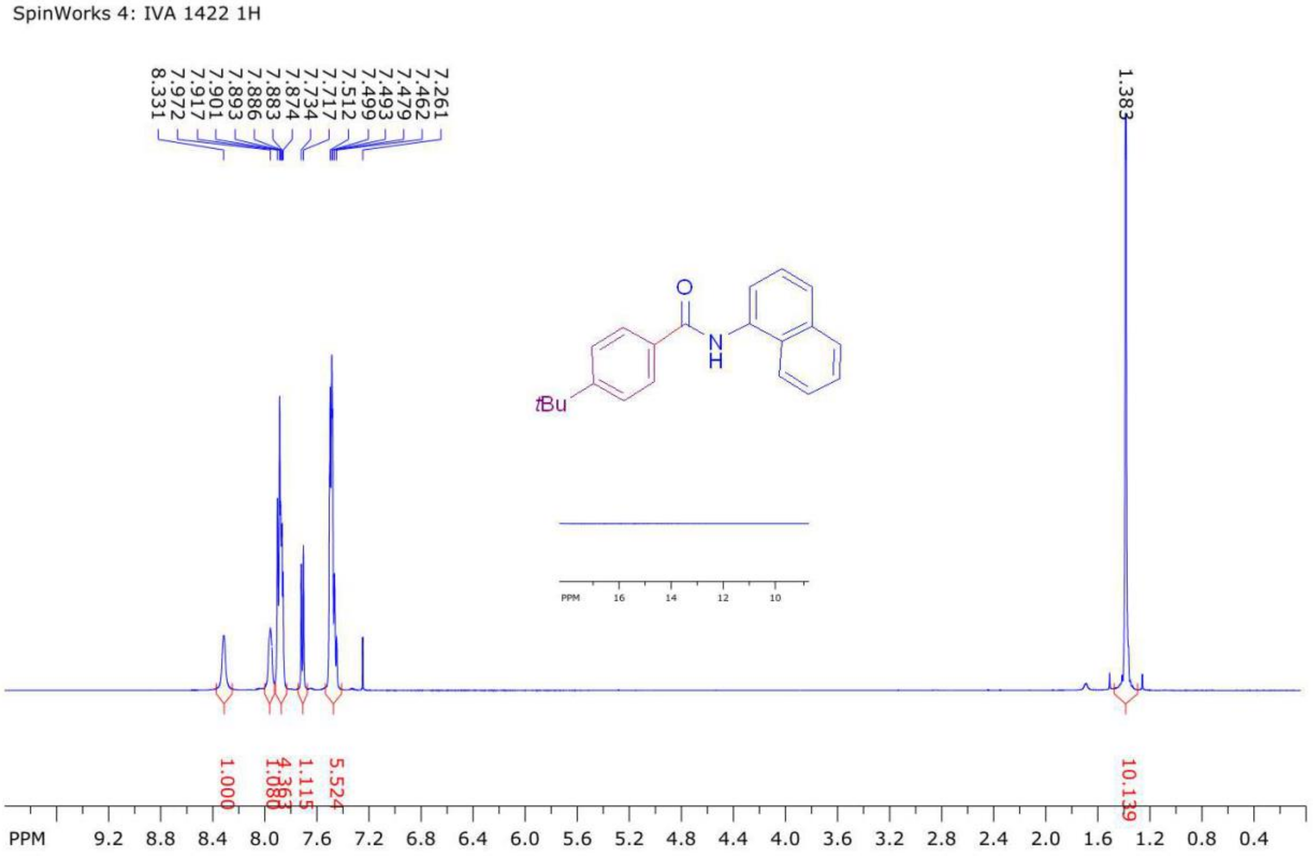

file: D:\NAPO\NMR\500-2\mkr12812\9\fid expt: <zg30>
transmitter freq.: 500.133001 MHz
time domain size: 65536 points
width: 12335.53 Hz = 24.6645 ppm = 0.188225 Hz/pt
number of scans: 24

freq. of 0 ppm: 500.130023 MHz
processed size: 65536 complex points
LB: 0.300 GF: 0.0000
Hz/cm: 199.770 ppm/cm: 0.39943

Compound 5b (NMR in $CDCl_3$)

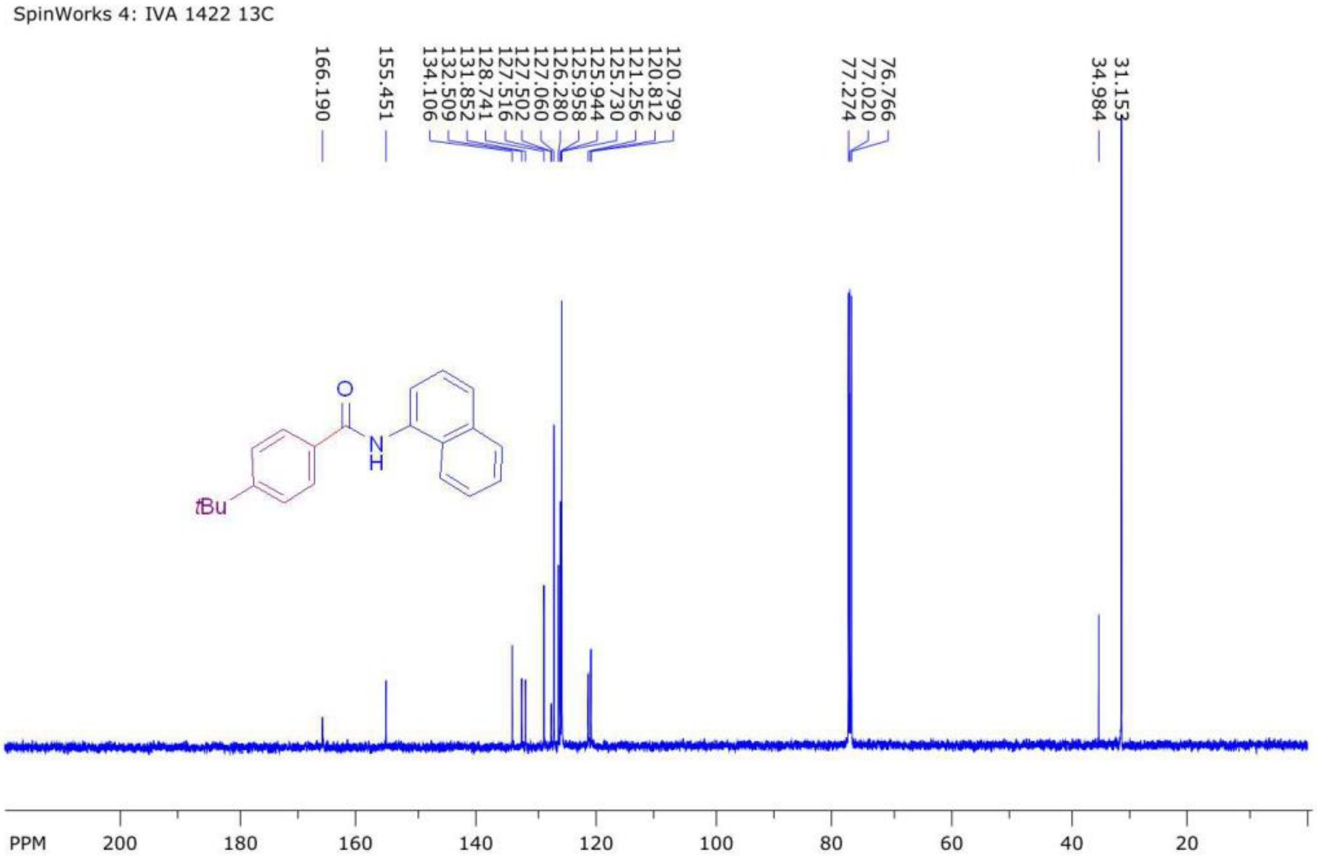

file: D:\NAPO\NMR\500-2\mkr12812\10\fid expt: <zgpg30>
transmitter freq.: 125.772879 MHz
time domain size: 65536 points
width: 36057.69 Hz = 286.6889 ppm = 0.550197 Hz/pt
number of scans: 512

freq. of 0 ppm: 125.757798 MHz
processed size: 32768 complex points
LB: 2.000 GF: 0.0000
Hz/cm: 1107.258 ppm/cm: 8.80363

Compound 5c (NMR in $CDCl_3$)

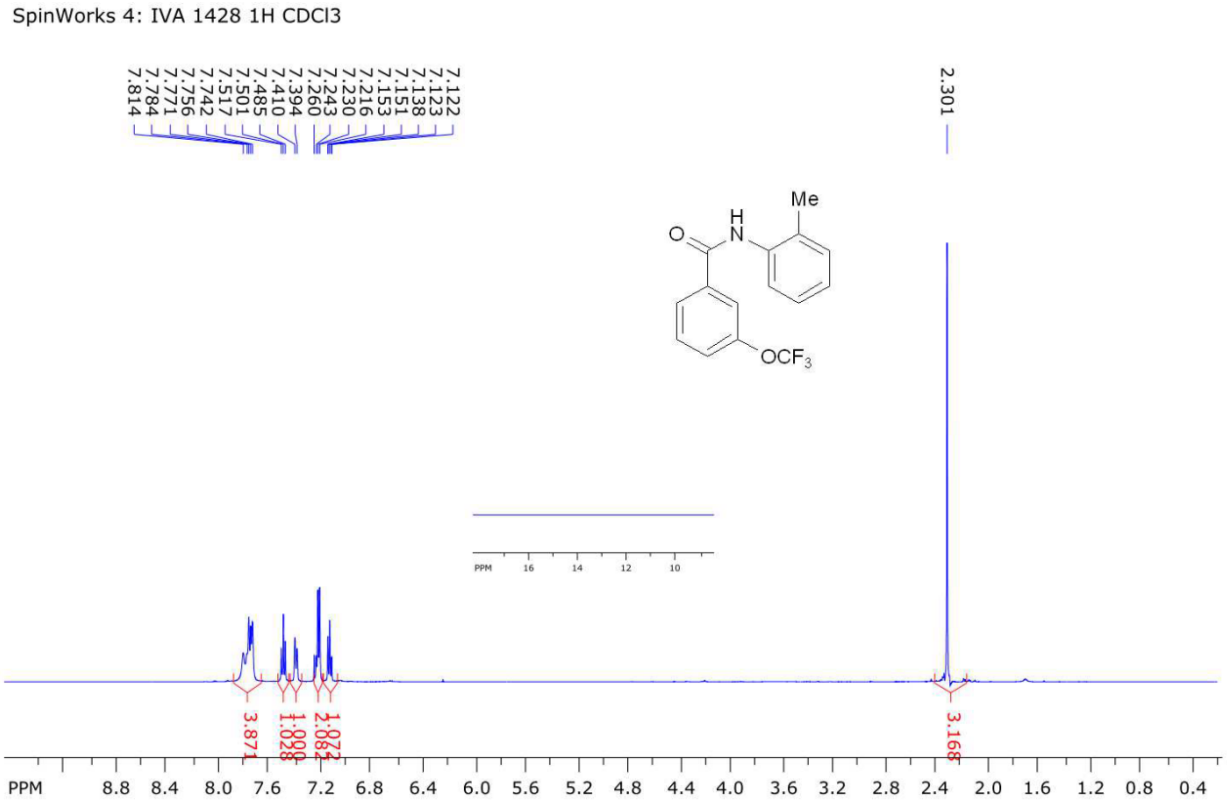

file: D:\NAPO\NMR\500-2\mkr11801\21\fid expt: <zg30>
transmitter freq.: 500.133001 MHz
time domain size: 65536 points
width: 12335.53 Hz = 24.6645 ppm = 0.188225 Hz/pt
number of scans: 24

freq. of 0 ppm: 500.130023 MHz
processed size: 65536 complex points
LB: 0.300 GF: 0.0000
Hz/cm: 190.126 ppm/cm: 0.38015

Compound 5c (NMR in $CDCl_3$)

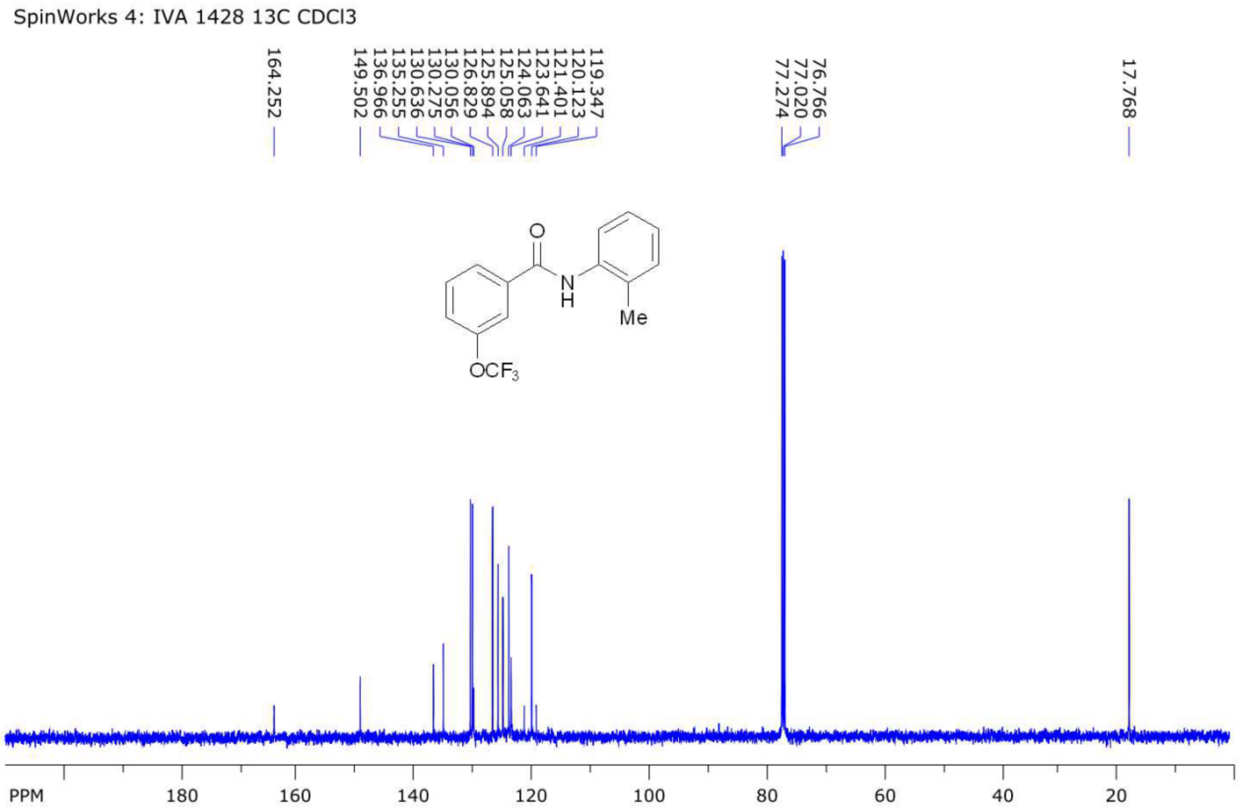

file: D:\NAPO\NMR\500-2\mkr11801\22\fid expt: <zgpg30>
transmitter freq.: 125.772879 MHz
time domain size: 65536 points
width: 36057.69 Hz = 286.6889 ppm = 0.550197 Hz/pt
number of scans: 256

freq. of 0 ppm: 125.757795 MHz
processed size: 32768 complex points
LB: 2.000 GF: 0.0000
Hz/cm: 1055.266 ppm/cm: 8.39025

Compound 5d (NMR in $CDCl_3$)

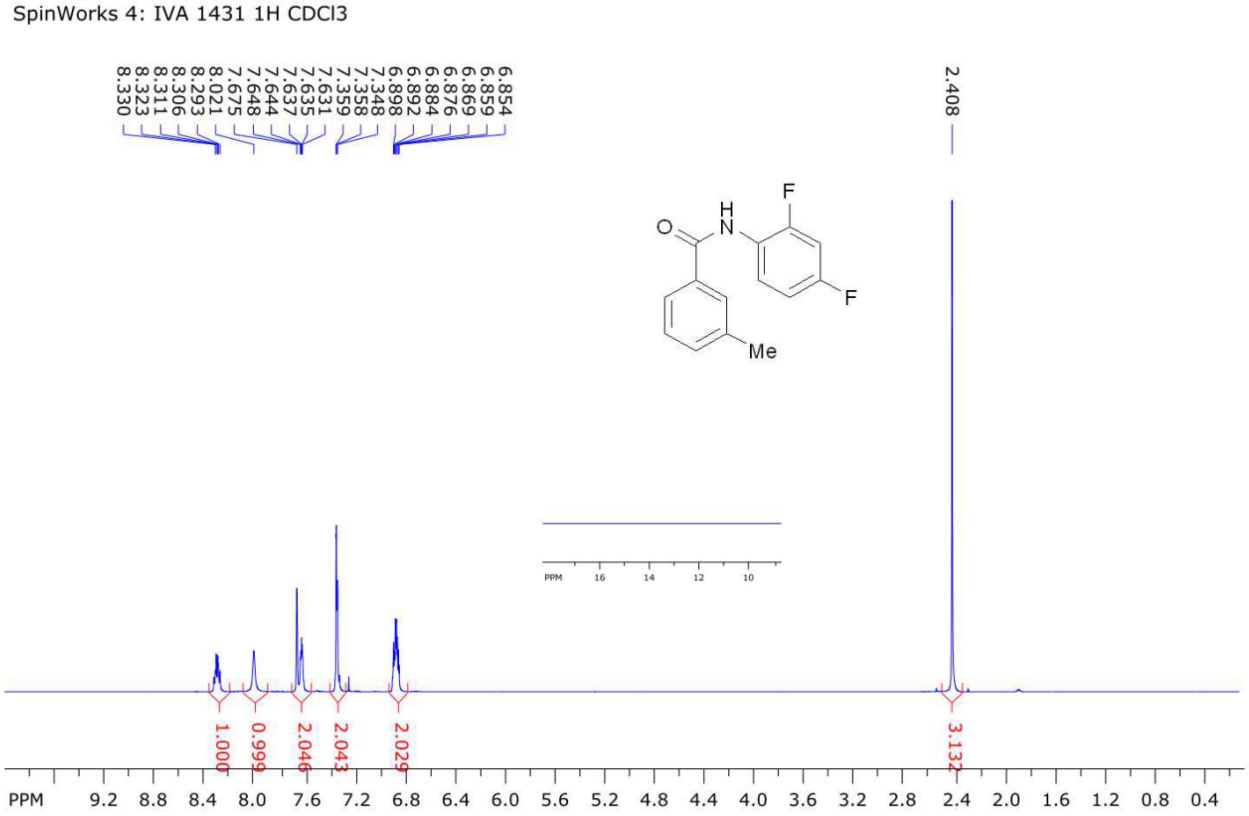

file: D:\NAPO\NMR\500-2\mkr11712\11\fid expt: <zg30>
transmitter freq.: 500.133001 MHz
time domain size: 65536 points
width: 12335.53 Hz = 24.6645 ppm = 0.188225 Hz/pt
number of scans: 24

freq. of 0 ppm: 500.130024 MHz
processed size: 65536 complex points
LB: 0.300 GF: 0.0000
Hz/cm: 198.613 ppm/cm: 0.39712

Compound 5d (NMR in $CDCl_3$)

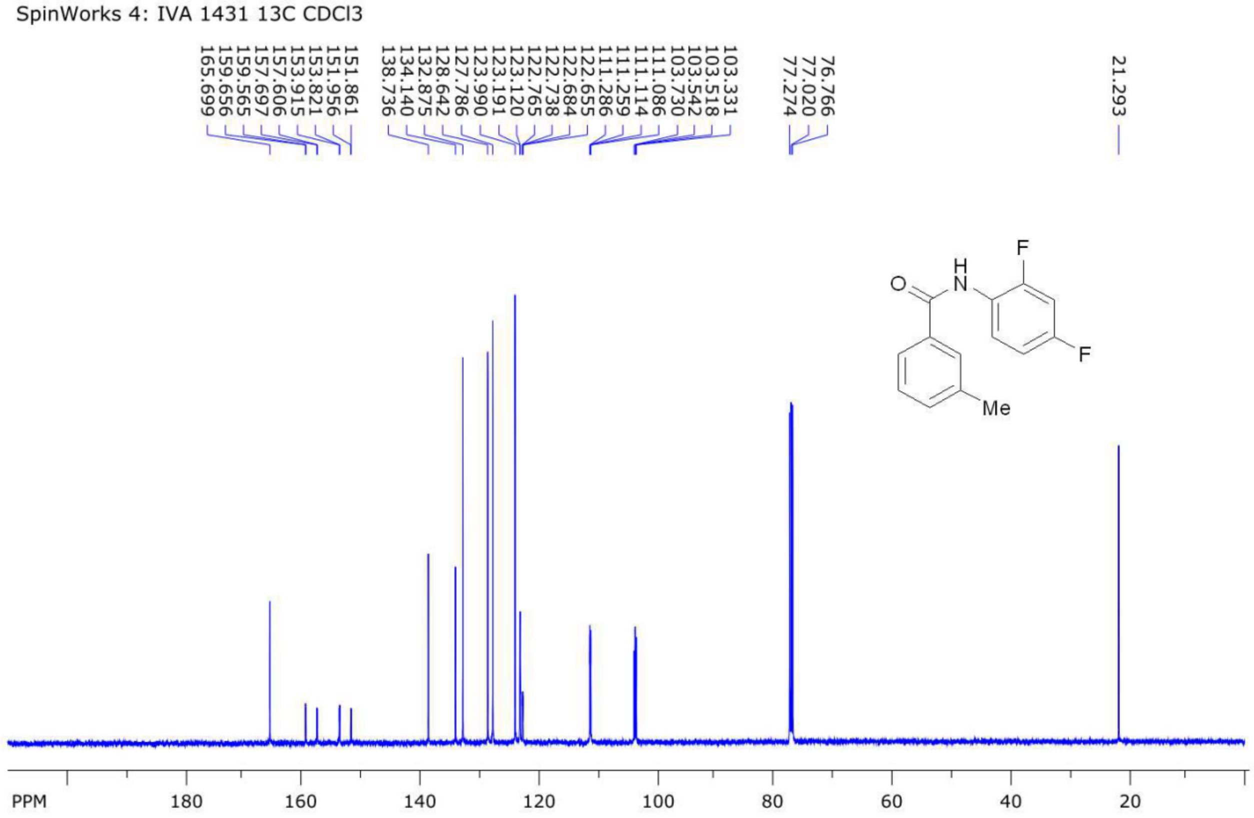

file: D:\NAPO\NMR\500-2\mkr11712\12\fid expt: <zgpg30>
transmitter freq.: 125.772879 MHz
time domain size: 65536 points
width: 36057.69 Hz = 286.6889 ppm = 0.550197 Hz/pt
number of scans: 1024

freq. of 0 ppm: 125.757800 MHz
processed size: 32768 complex points
LB: 2.000 GF: 0.0000
Hz/cm: 1058.574 ppm/cm: 8.41656

Compound 5e (NMR in $CDCl_3$)

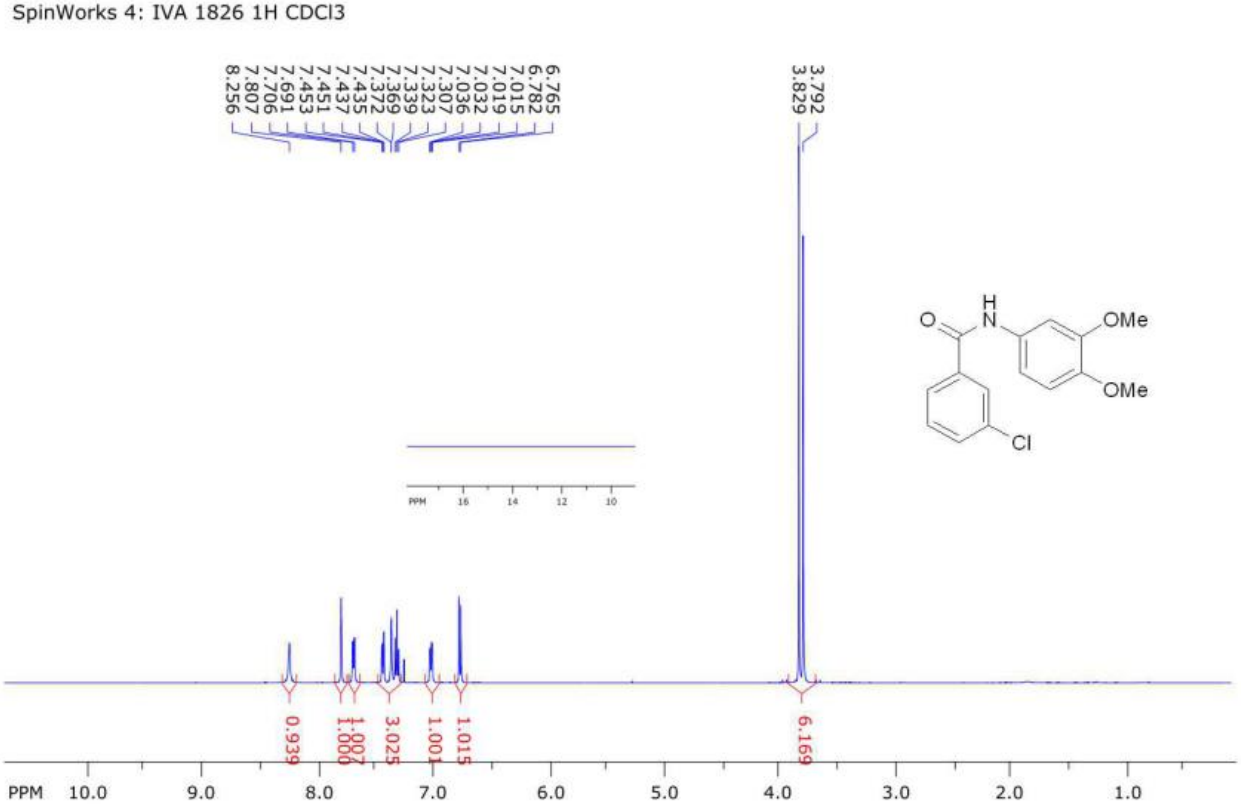

file: D:\NAPO\NMR\500-2\mkr11204\11\fid expt: <zg30>
transmitter freq.: 500.133001 MHz
time domain size: 65536 points
width: 12335.53 Hz = 24.6645 ppm = 0.188225 Hz/pt
number of scans: 24

freq. of 0 ppm: 500.130023 MHz
processed size: 65536 complex points
LB: 0.300 GF: 0.0000
Hz/cm: 213.325 ppm/cm: 0.42654

Compound 5e (NMR in $CDCl_3$)

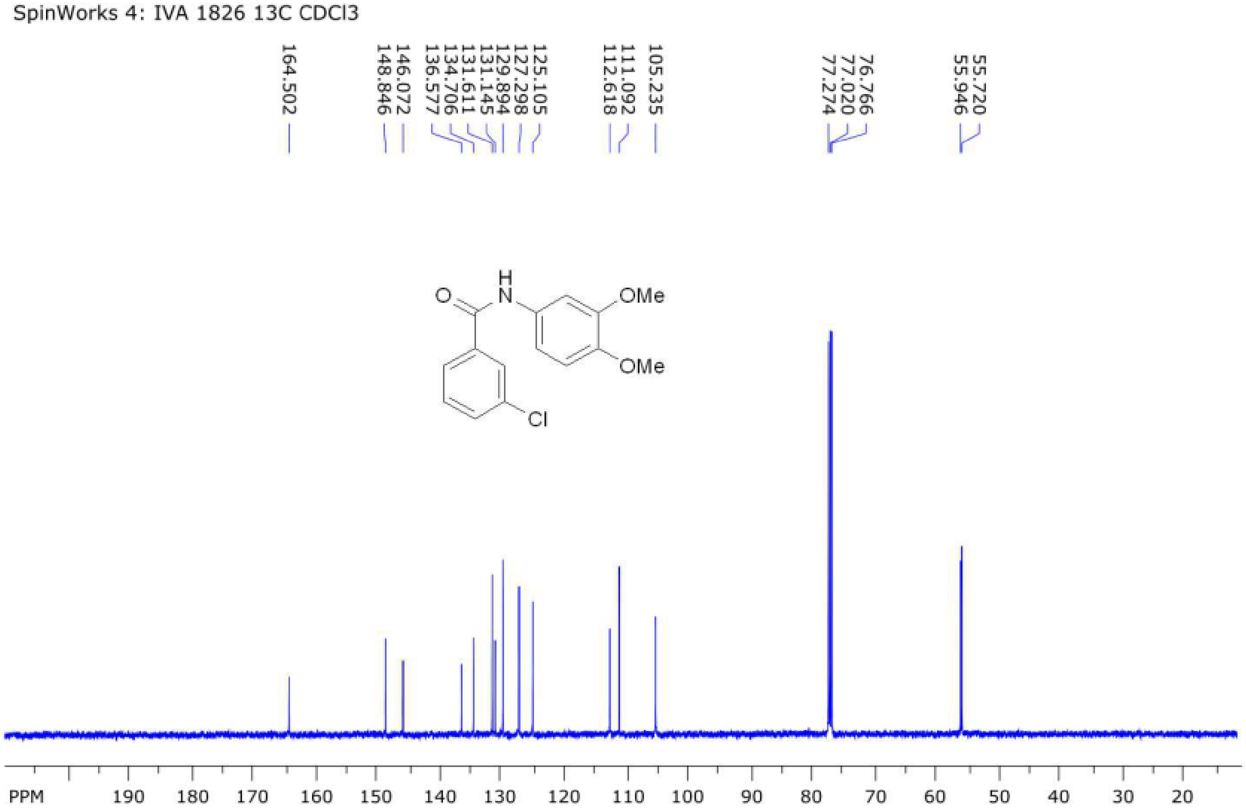

file: D:\NAPO\NMR\500-2\mkr11204\12\fid expt: <zgpg30>
transmitter freq.: 125.772879 MHz
time domain size: 65536 points
width: 36057.69 Hz = 286.6889 ppm = 0.550197 Hz/pt
number of scans: 512

freq. of 0 ppm: 125.757800 MHz
processed size: 32768 complex points
LB: 2.000 GF: 0.0000
Hz/cm: 1002.184 ppm/cm: 7.96820

Compound 5f (NMR in $CDCl_3$)

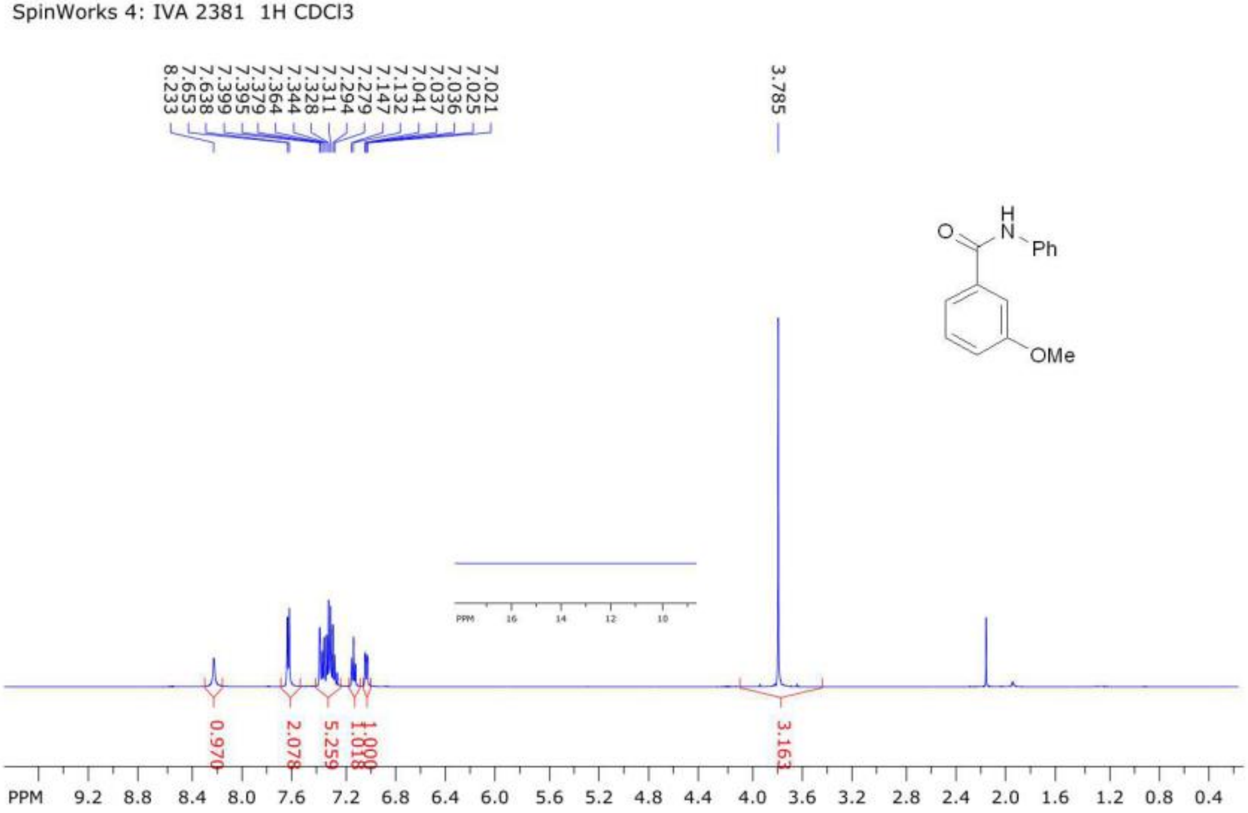

file: D:\NAPO\NMR\500-2\mkr23004\17\fid expt: <zg30>
transmitter freq.: 500.133001 MHz
time domain size: 65536 points
width: 12335.53 Hz = 24.6645 ppm = 0.188225 Hz/pt
number of scans: 24

freq. of 0 ppm: 500.130022 MHz
processed size: 65536 complex points
LB: 0.300 GF: 0.0000
Hz/cm: 194.652 ppm/cm: 0.38920

Compound 5f (NMR in $CDCl_3$)

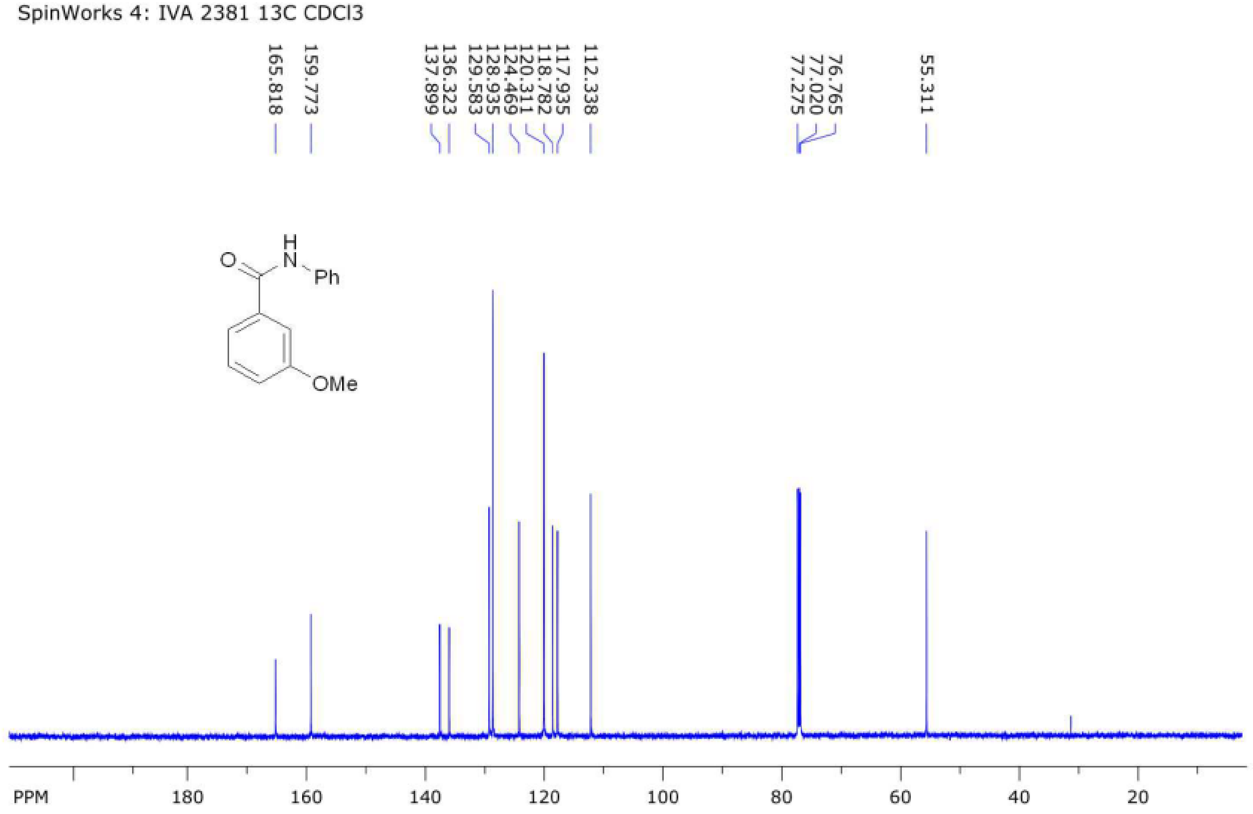

file: D:\NAPO\NMR\500-2\mkr23004\18\fid expt: <zgpg30>
transmitter freq.: 125.772879 MHz
time domain size: 65536 points
width: 36057.69 Hz = 286.6889 ppm = 0.550197 Hz/pt
number of scans: 512

freq. of 0 ppm: 125.757803 MHz
processed size: 32768 complex points
LB: 2.000 GF: 0.0000
Hz/cm: 1051.958 ppm/cm: 8.36395

Compound 5g (NMR in DMSO-d_6)

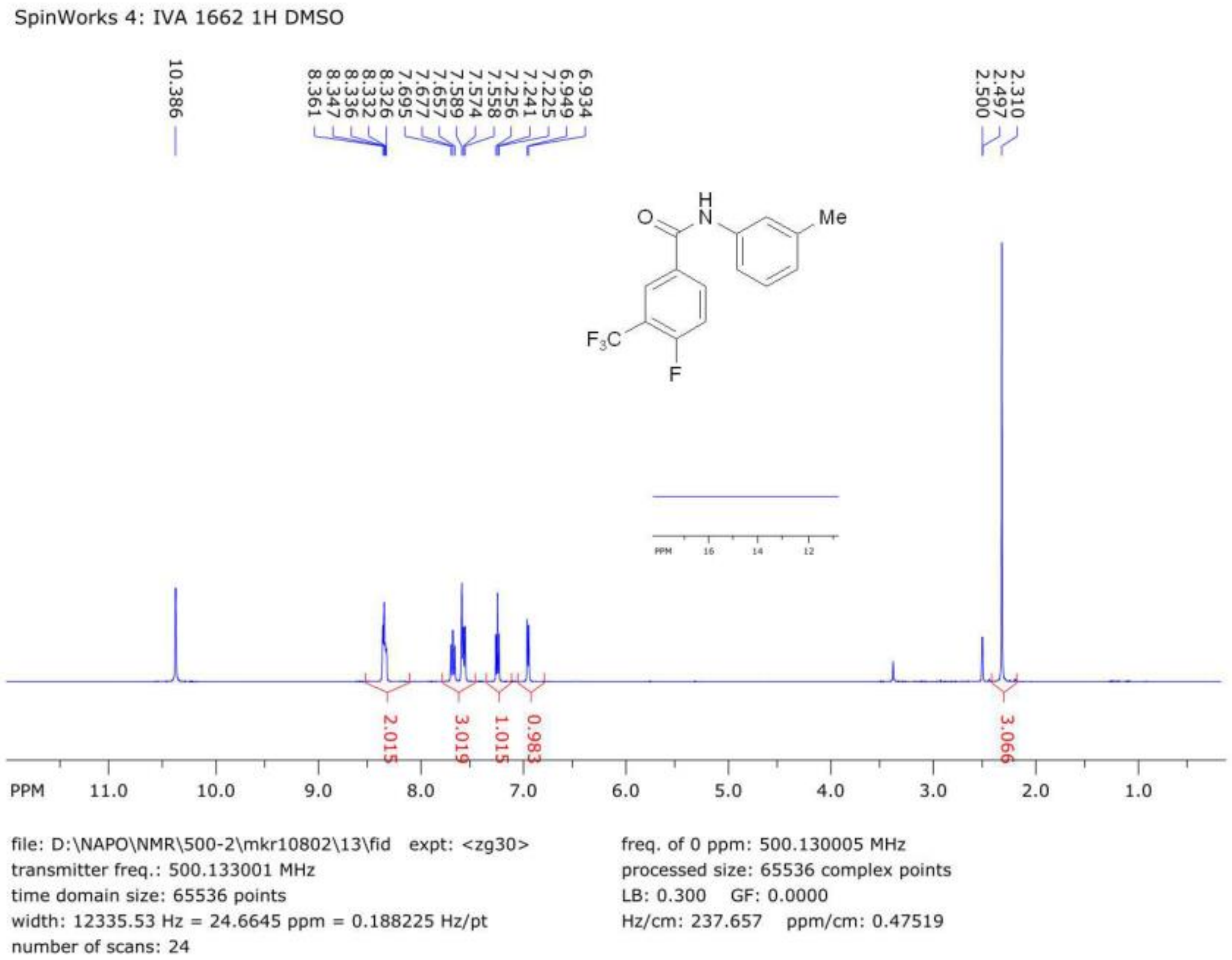

Compound 5g (NMR in DMSO-d_6)

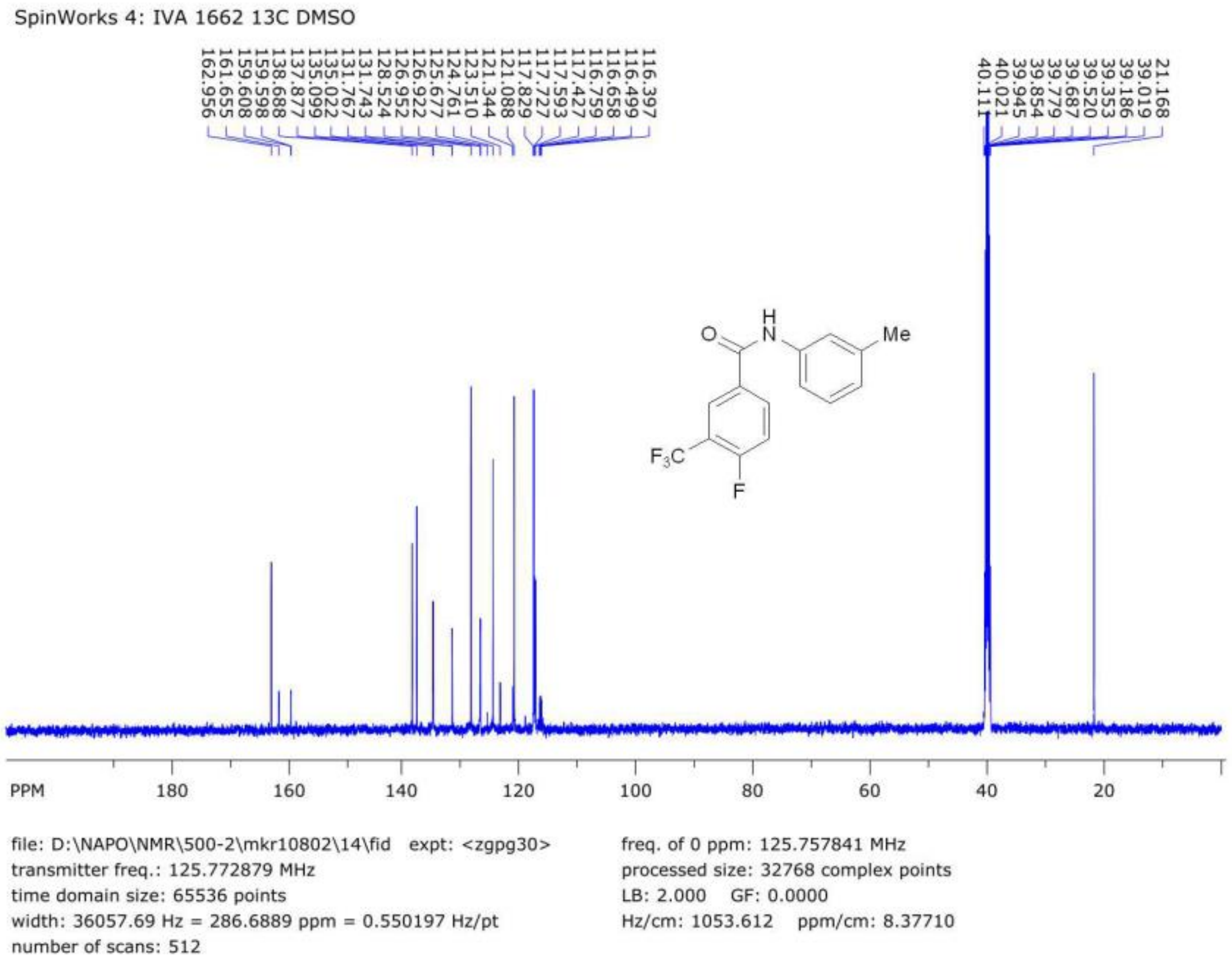

Compound 5f (NMR in $CDCl_3$)

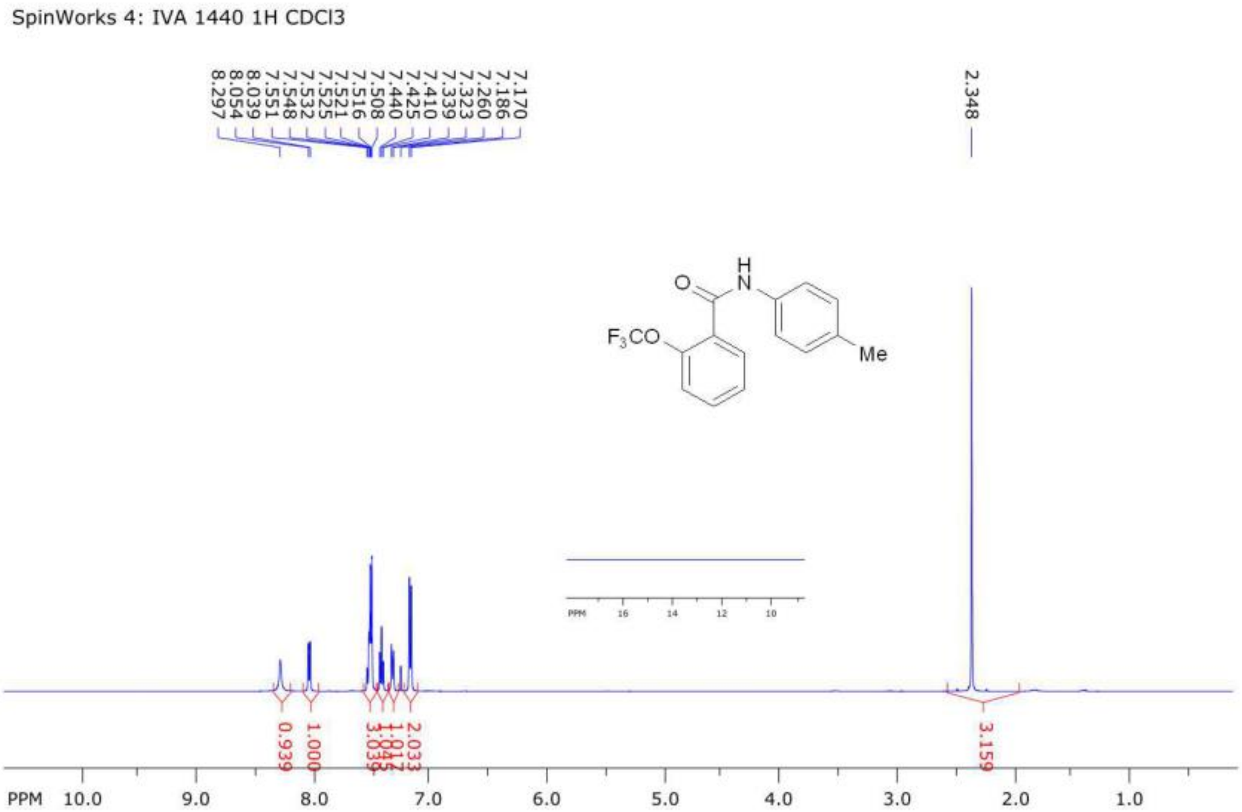

file: D:\NAPO\NMR\500-2\mkr11712\1\fid expt: <zg30>
transmitter freq.: 500.133001 MHz
time domain size: 65536 points
width: 12335.53 Hz = 24.6645 ppm = 0.188225 Hz/pt
number of scans: 24

freq. of 0 ppm: 500.130023 MHz
processed size: 65536 complex points
LB: 0.300 GF: 0.0000
Hz/cm: 211.628 ppm/cm: 0.42314

Compound 5h (NMR in $CDCl_3$)

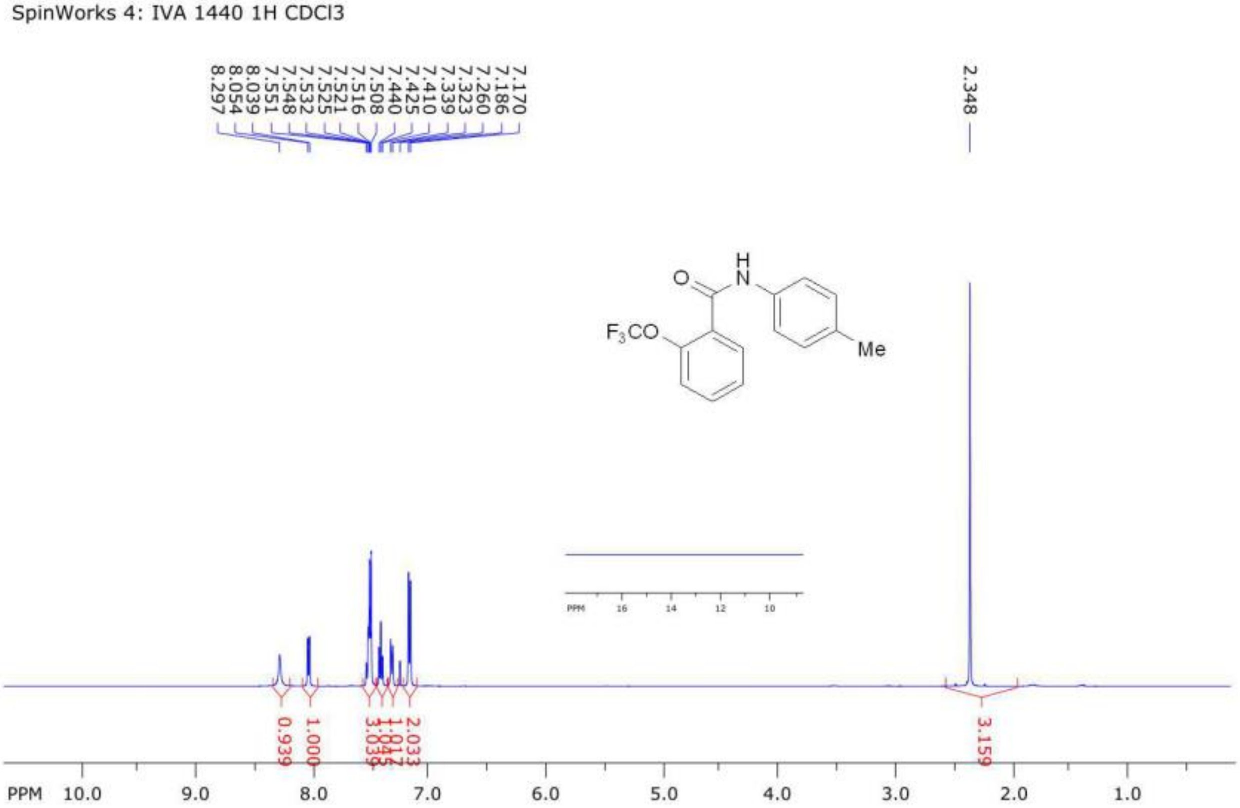

file: D:\NAPO\NMR\500-2\mkr11712\1\fid expt: <zg30>
transmitter freq.: 500.133001 MHz
time domain size: 65536 points
width: 12335.53 Hz = 24.6645 ppm = 0.188225 Hz/pt
number of scans: 24

freq. of 0 ppm: 500.130023 MHz
processed size: 65536 complex points
LB: 0.300 GF: 0.0000
Hz/cm: 211.628 ppm/cm: 0.42314

Compound 5h (NMR in $CDCl_3$)

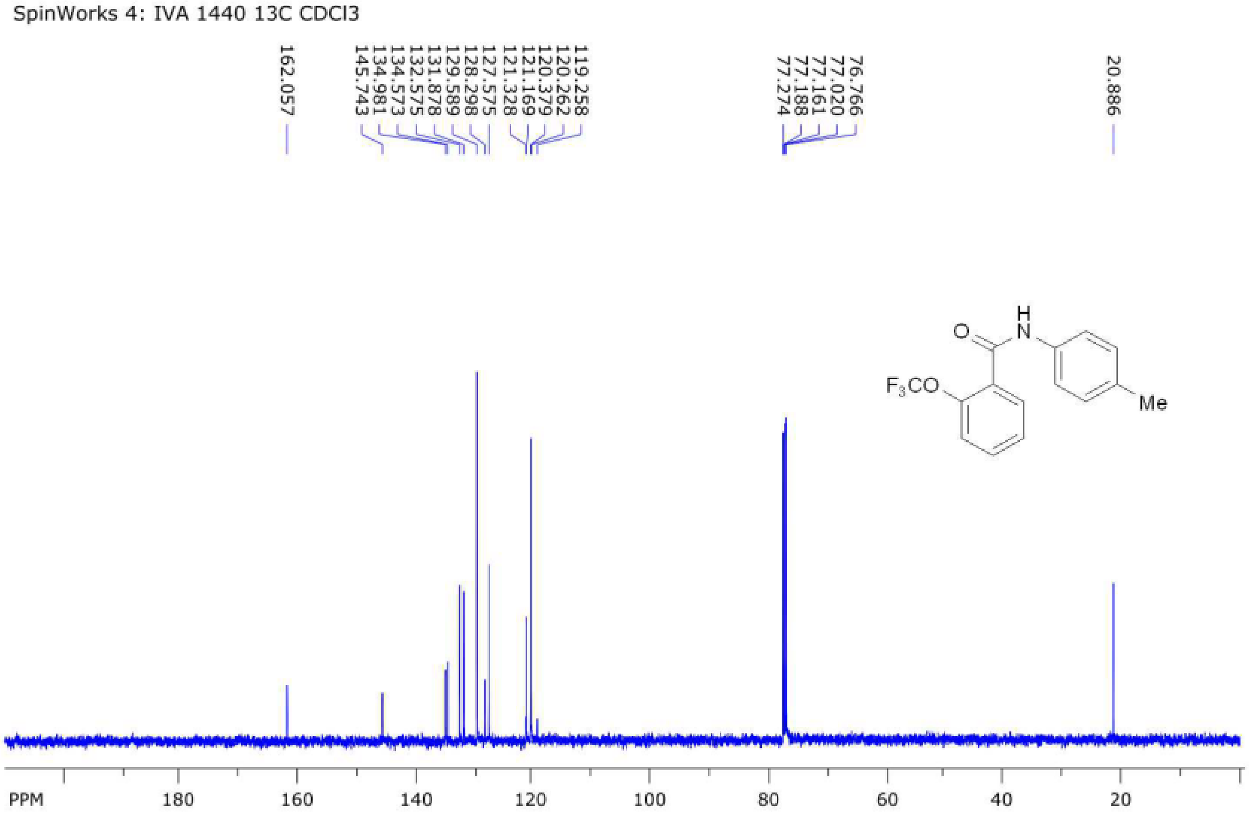

file: D:\NAPO\NMR\500-2\mkr11712\2\fid expt: <zgpg30>
transmitter freq.: 125.772879 MHz
time domain size: 65536 points
width: 36057.69 Hz = 286.6889 ppm = 0.550197 Hz/pt
number of scans: 140

freq. of 0 ppm: 125.757796 MHz
processed size: 32768 complex points
LB: 2.000 GF: 0.0000
Hz/cm: 1061.882 ppm/cm: 8.44286

Compound 5i (NMR in $CDCl_3$)

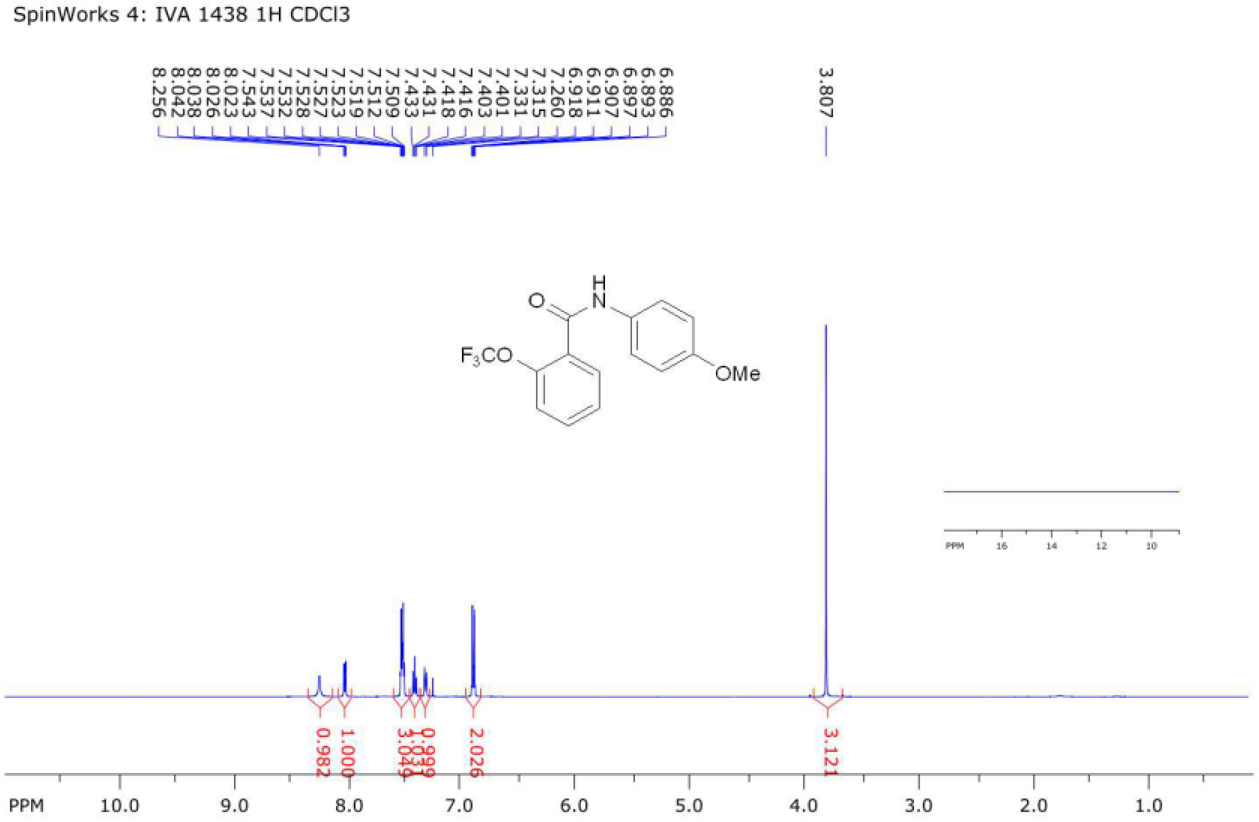

file: D:\NAPO\NMR\500-2\mkr12812\15\fid expt: <zg30>
transmitter freq.: 500.133001 MHz
time domain size: 65536 points
width: 12335.53 Hz = 24.6645 ppm = 0.188225 Hz/pt
number of scans: 24

freq. of 0 ppm: 500.130023 MHz
processed size: 65536 complex points
LB: 0.300 GF: 0.0000
Hz/cm: 218.418 ppm/cm: 0.43672

Compound 5i (NMR in $CDCl_3$)

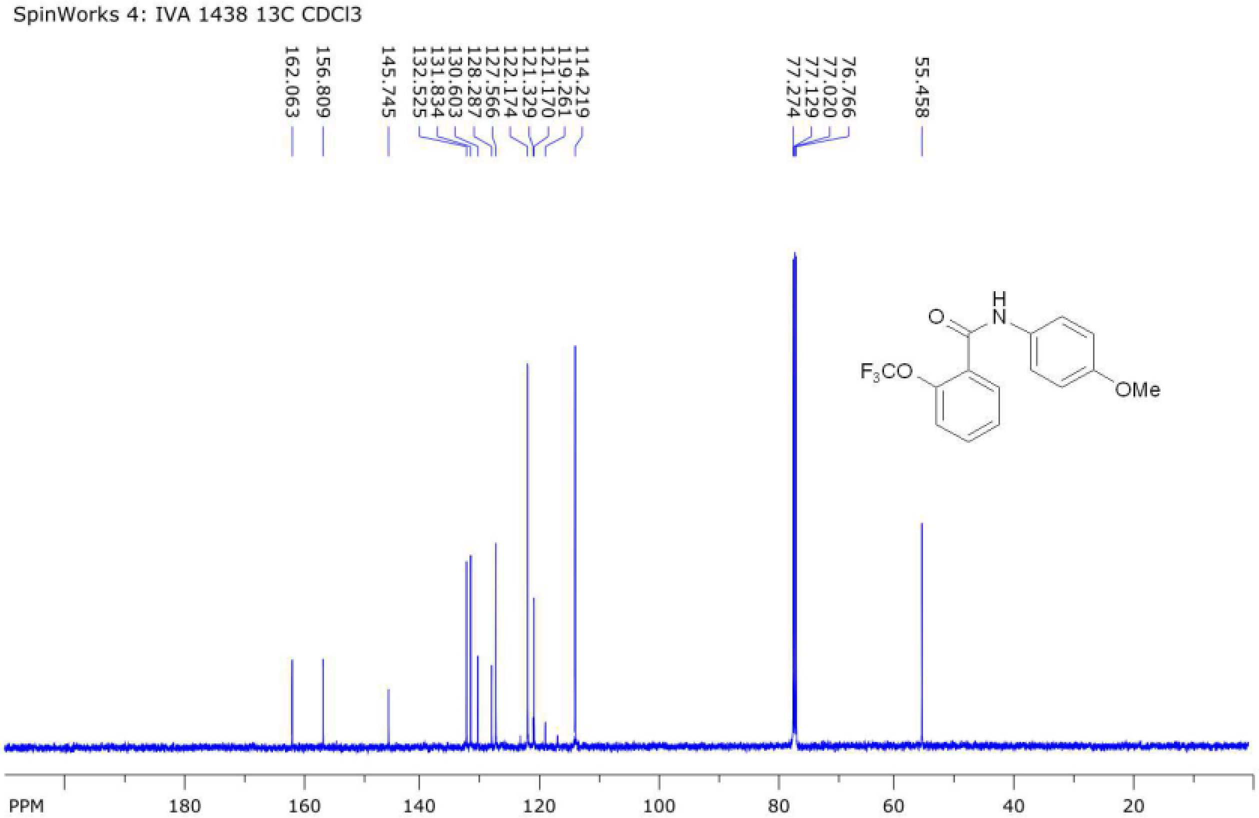

file: D:\NAPO\NMR\500-2\mkr12812\16\fid expt: <zgpg30>
transmitter freq.: 125.772879 MHz
time domain size: 65536 points
width: 36057.69 Hz = 286.6889 ppm = 0.550197 Hz/pt
number of scans: 512

freq. of 0 ppm: 125.757796 MHz
processed size: 32768 complex points
LB: 2.000 GF: 0.0000
Hz/cm: 1058.574 ppm/cm: 8.41656

Compound 5j (NMR in $CDCl_3$)

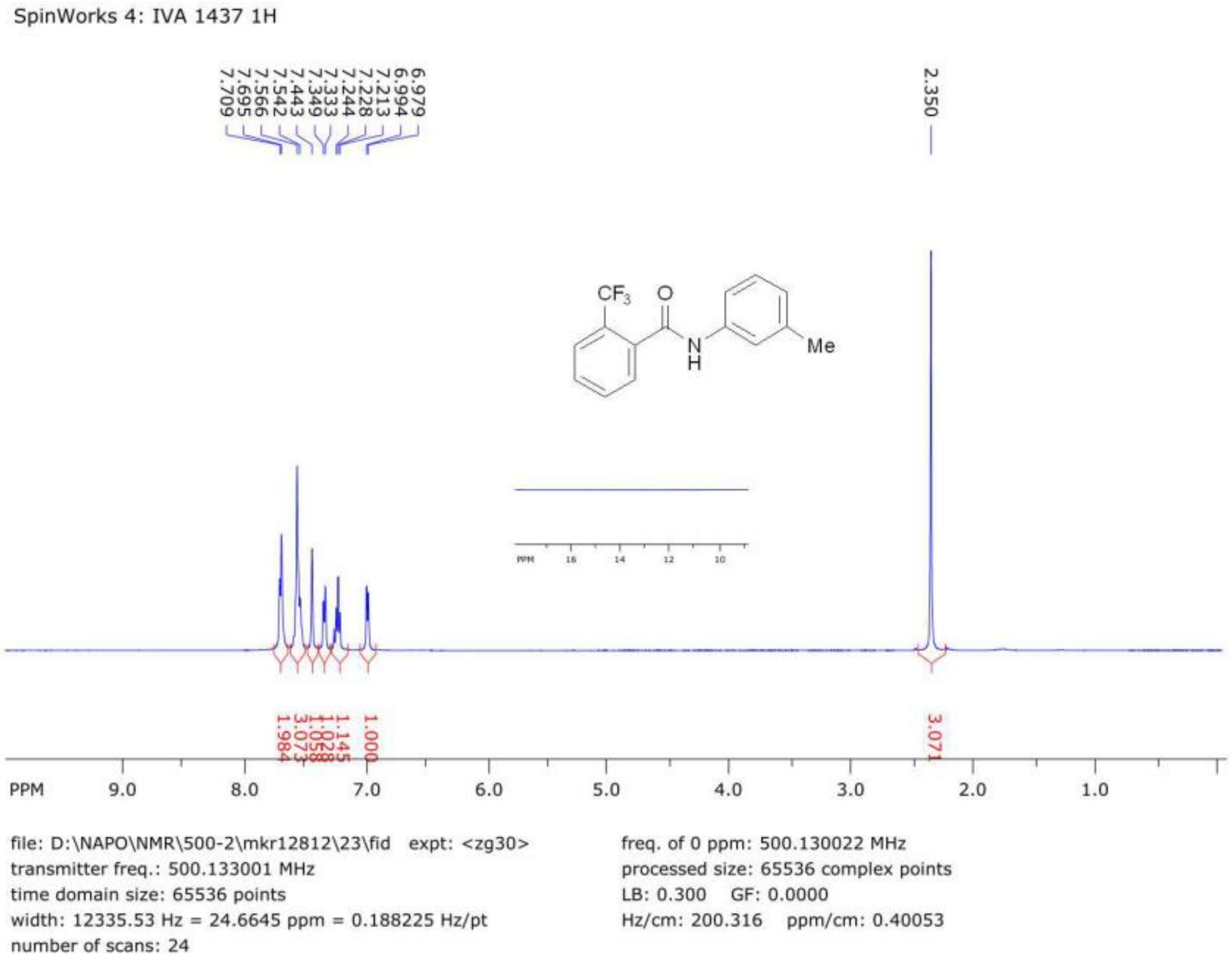

Compound 5j (NMR in $CDCl_3$)

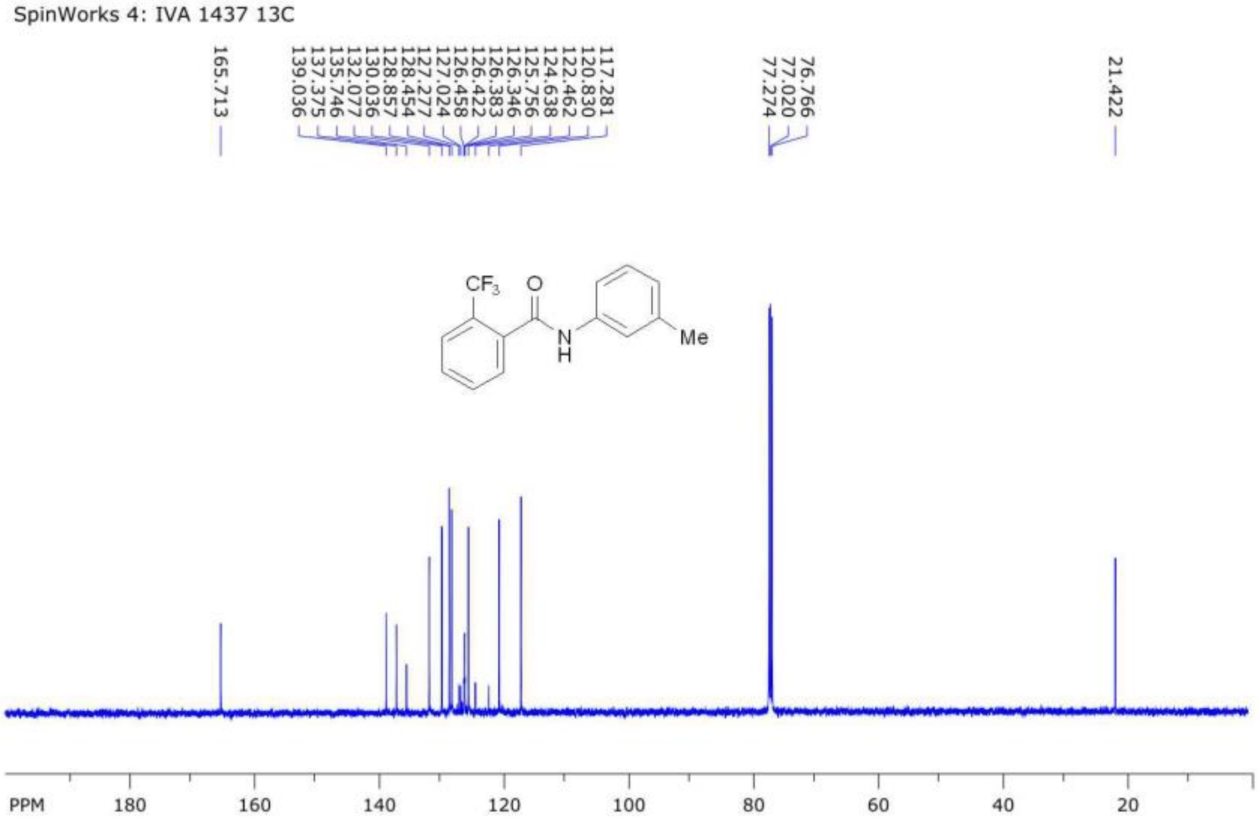

file: D:\NAPO\NMR\500-2\mkr12812\24\fid expt: <zgpg30>
transmitter freq.: 125.772879 MHz
time domain size: 65536 points
width: 36057.69 Hz = 286.6889 ppm = 0.550197 Hz/pt
number of scans: 512

freq. of 0 ppm: 125.757798 MHz
processed size: 32768 complex points
LB: 2.000 GF: 0.0000
Hz/cm: 1008.339 ppm/cm: 8.01714

Compound 5k (NMR in $CDCl_3$)

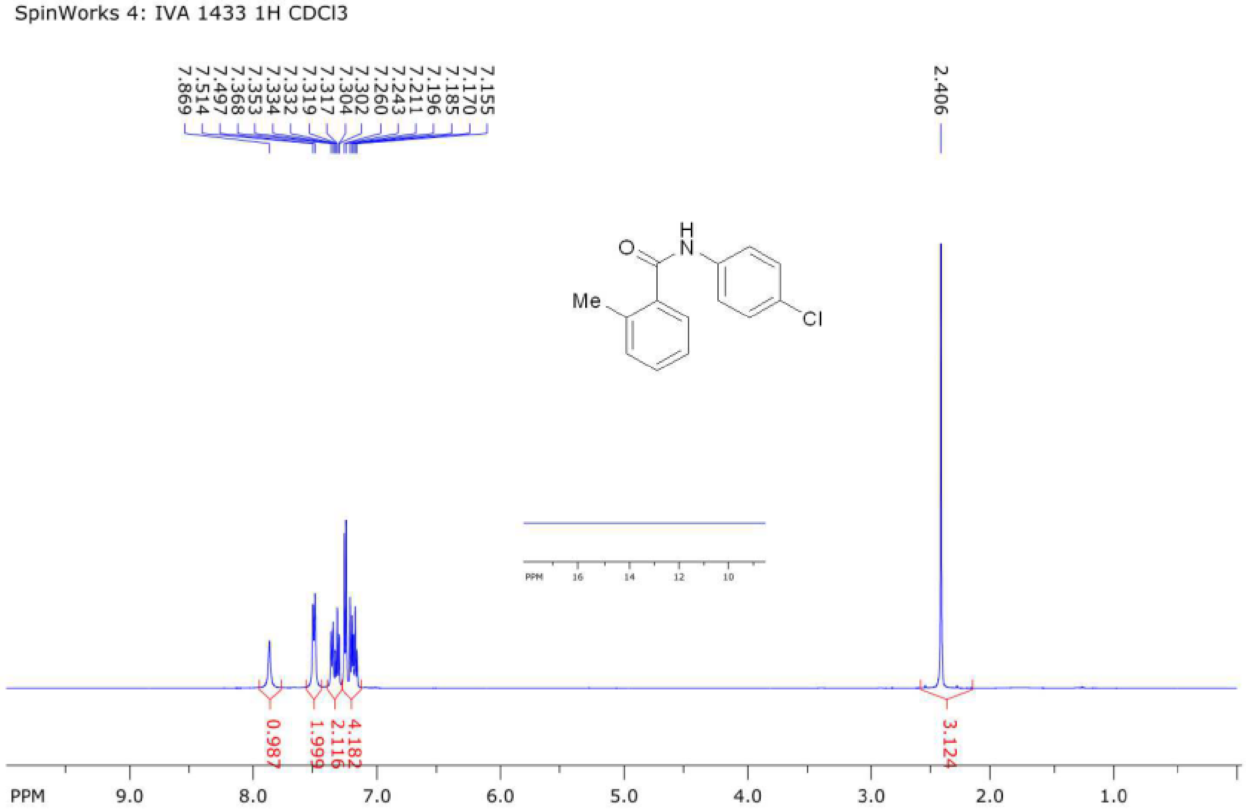

file: D:\NAPO\NMR\500-2\mkr12812\25\fid expt: <zg30>
transmitter freq.: 500.133001 MHz
time domain size: 65536 points
width: 12335.53 Hz = 24.6645 ppm = 0.188225 Hz/pt
number of scans: 24

freq. of 0 ppm: 500.130032 MHz
processed size: 65536 complex points
LB: 0.300 GF: 0.0000
Hz/cm: 200.311 ppm/cm: 0.40052

Compound 5k (NMR in $CDCl_3$)

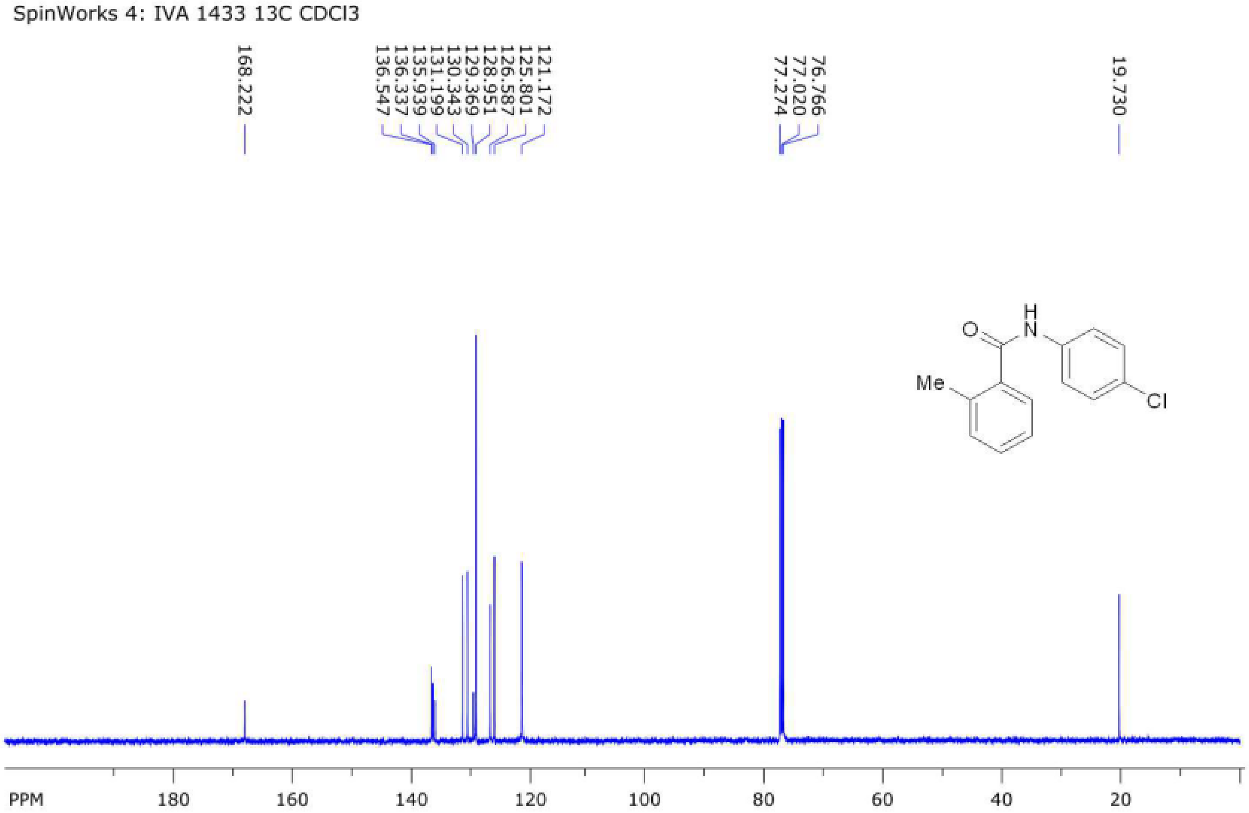

file: D:\NAPO\NMR\500-2\mkr12812\26\fid expt: <zgpg30>
transmitter freq.: 125.772879 MHz
time domain size: 65536 points
width: 36057.69 Hz = 286.6889 ppm = 0.550197 Hz/pt
number of scans: 512

freq. of 0 ppm: 125.757799 MHz
processed size: 32768 complex points
LB: 2.000 GF: 0.0000
Hz/cm: 1055.266 ppm/cm: 8.39025

Compound 5l (NMR in $CDCl_3$)

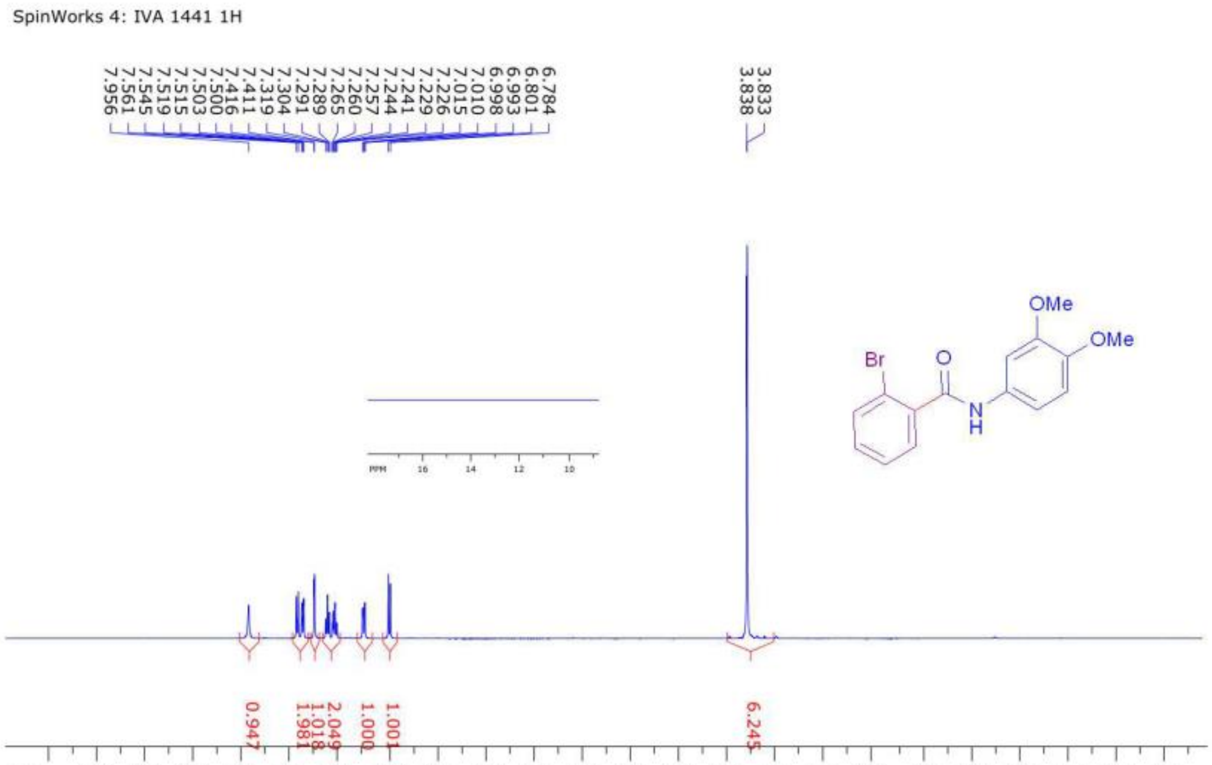

file: D:\NAPO\NMR\500-2\mkr12901\11\fid expt: <zg30>
transmitter freq.: 500.133001 MHz
time domain size: 65536 points
width: 12335.53 Hz = 24.6645 ppm = 0.188225 Hz/pt
number of scans: 12

freq. of 0 ppm: 500.130020 MHz
processed size: 65536 complex points
LB: 0.300 GF: 0.0000
Hz/cm: 198.133 ppm/cm: 0.39616

Compound 5l (NMR in $CDCl_3$)

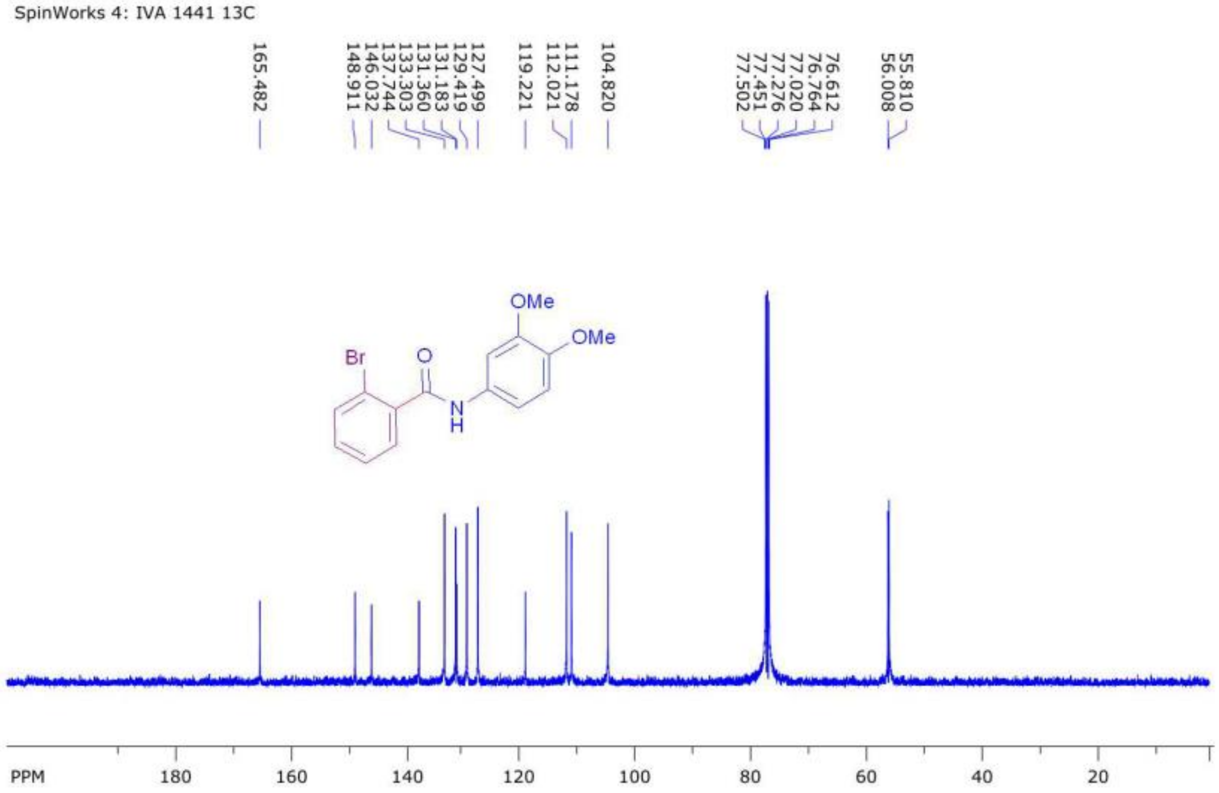

file: D:\NAPO\NMR\500-2\mkr12901\12\fid expt: <zgpg30>
transmitter freq.: 125.772879 MHz
time domain size: 65536 points
width: 36057.69 Hz = 286.6889 ppm = 0.550197 Hz/pt
number of scans: 80

freq. of 0 ppm: 125.757803 MHz
processed size: 32768 complex points
LB: 2.000 GF: 0.0000
Hz/cm: 1054.608 ppm/cm: 8.38502

Compound 5m (NMR in $CDCl_3$)

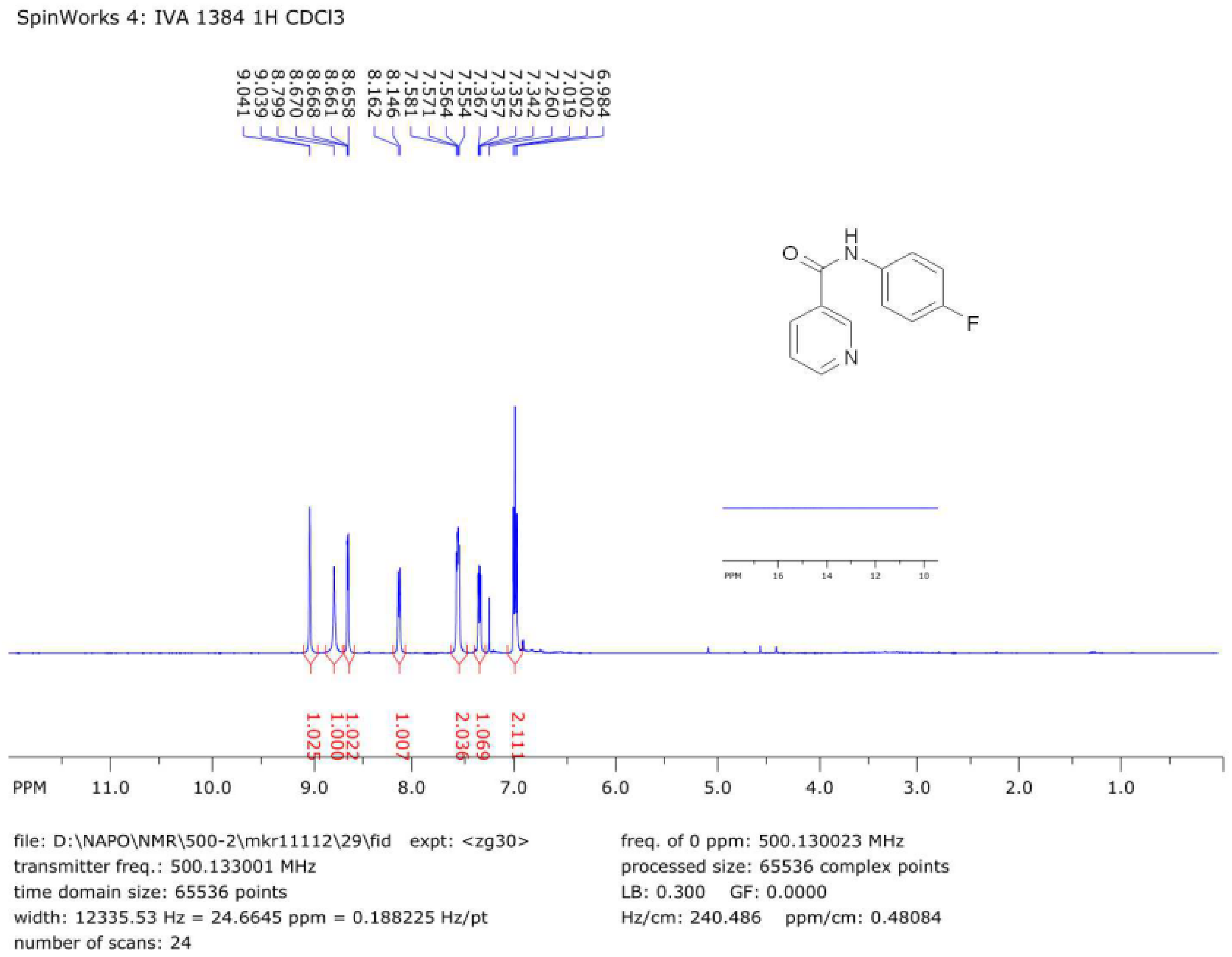

Compound 5m (NMR in $CDCl_3$)

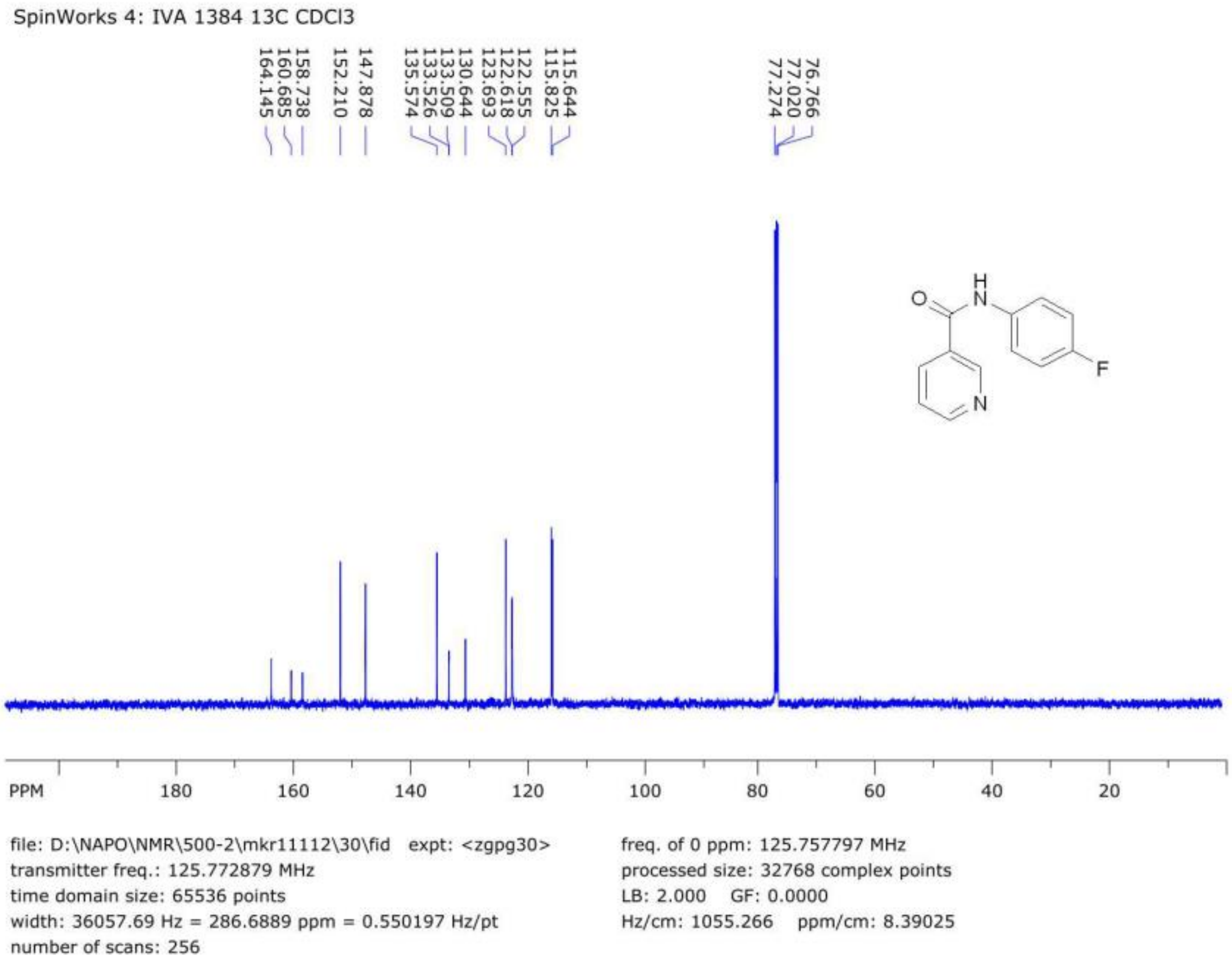

Compound 5n (NMR in $CDCl_3$)

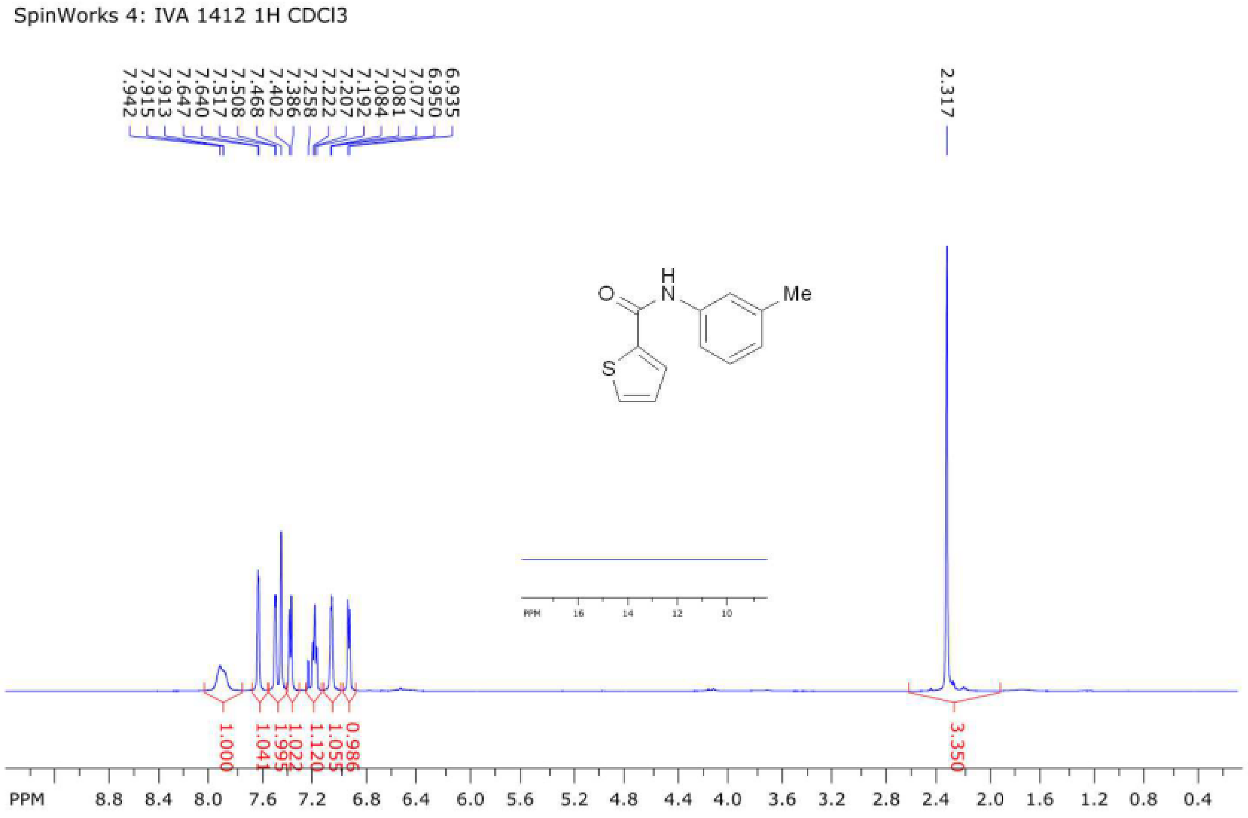

file: D:\NAPO\NMR\500-2\mkr13001\11\fid expt: <zg30>
transmitter freq.: 500.133001 MHz
time domain size: 65536 points
width: 12335.53 Hz = 24.6645 ppm = 0.188225 Hz/pt
number of scans: 24

freq. of 0 ppm: 500.130024 MHz
processed size: 65536 complex points
LB: 0.300 GF: 0.0000
Hz/cm: 190.691 ppm/cm: 0.38128

Compound 5n (NMR in $CDCl_3$)

SpinWorks 4: IVA 1412 13C CDCl3

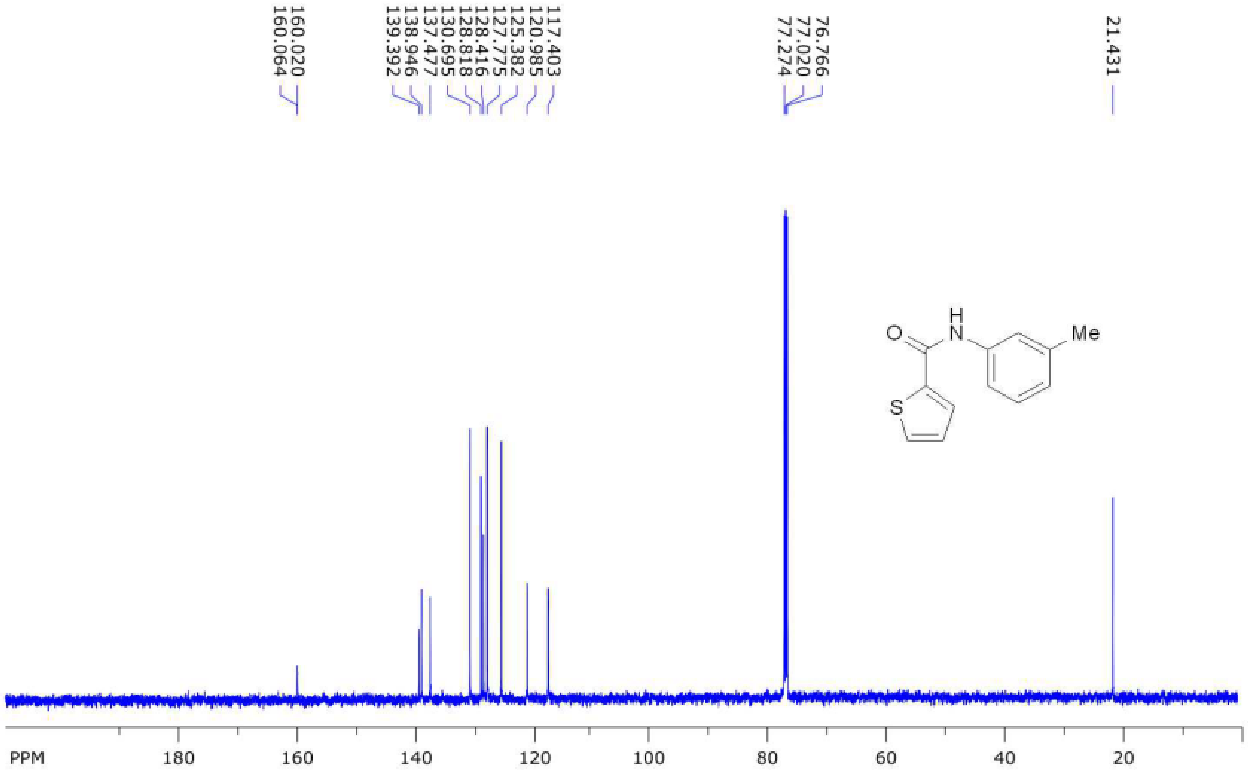

file: D:\NAPO\NMR\500-2\mkr13001\12\fid expt: <zgpg30>
transmitter freq.: 125.772879 MHz
time domain size: 65536 points
width: 36057.69 Hz = 286.6889 ppm = 0.550197 Hz/pt
number of scans: 512

freq. of 0 ppm: 125.757798 MHz
processed size: 32768 complex points
LB: 2.000 GF: 0.0000
Hz/cm: 1053.612 ppm/cm: 8.37710

Compound 5o (NMR in DMSO-d_6)

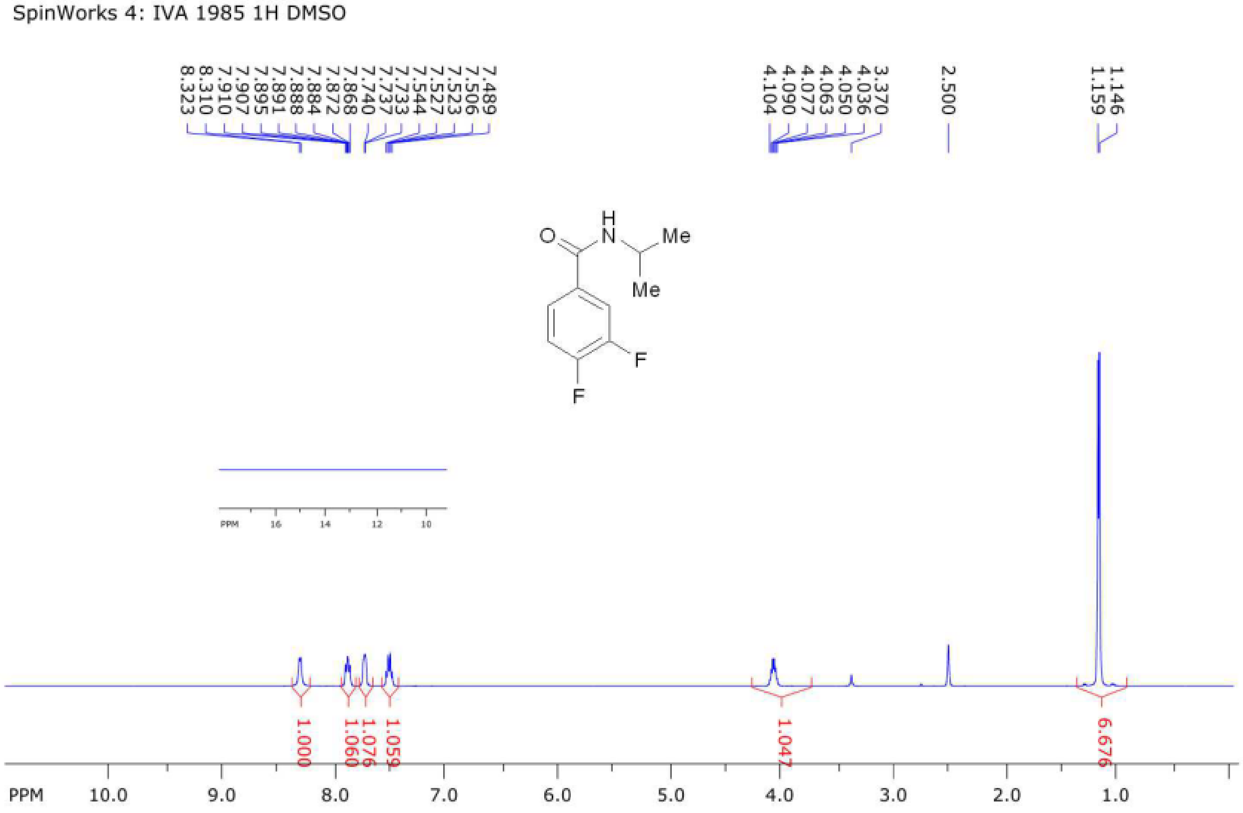

file: D:\NAPO\NMR\500-2\mkr11802\25\fid expt: <zg30>
transmitter freq.: 500.133001 MHz
time domain size: 65536 points
width: 12335.53 Hz = 24.6645 ppm = 0.188225 Hz/pt
number of scans: 24

freq. of 0 ppm: 500.130005 MHz
processed size: 65536 complex points
LB: 0.300 GF: 0.0000
Hz/cm: 220.681 ppm/cm: 0.44125

Compound 5o (NMR in DMSO-d_6)

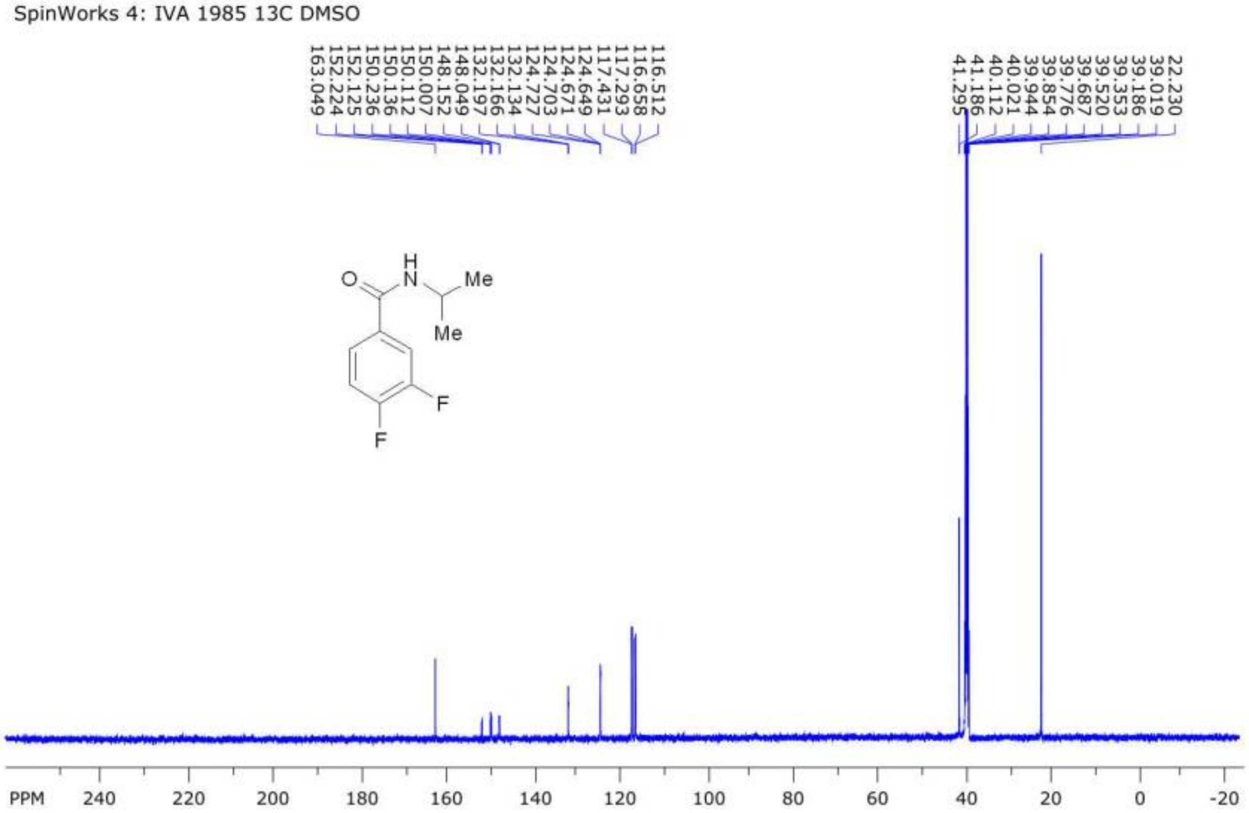

file: D:\NAPO\NMR\500-2\mkr11802\26\fid expt: <zgpg30>
transmitter freq.: 125.772879 MHz
time domain size: 65536 points
width: 36057.69 Hz = 286.6889 ppm = 0.550197 Hz/pt
number of scans: 512

freq. of 0 ppm: 125.757841 MHz
processed size: 32768 complex points
LB: 2.000 GF: 0.0000
Hz/cm: 1442.308 ppm/cm: 11.46756

Compound 5p (NMR in $CDCl_3$)

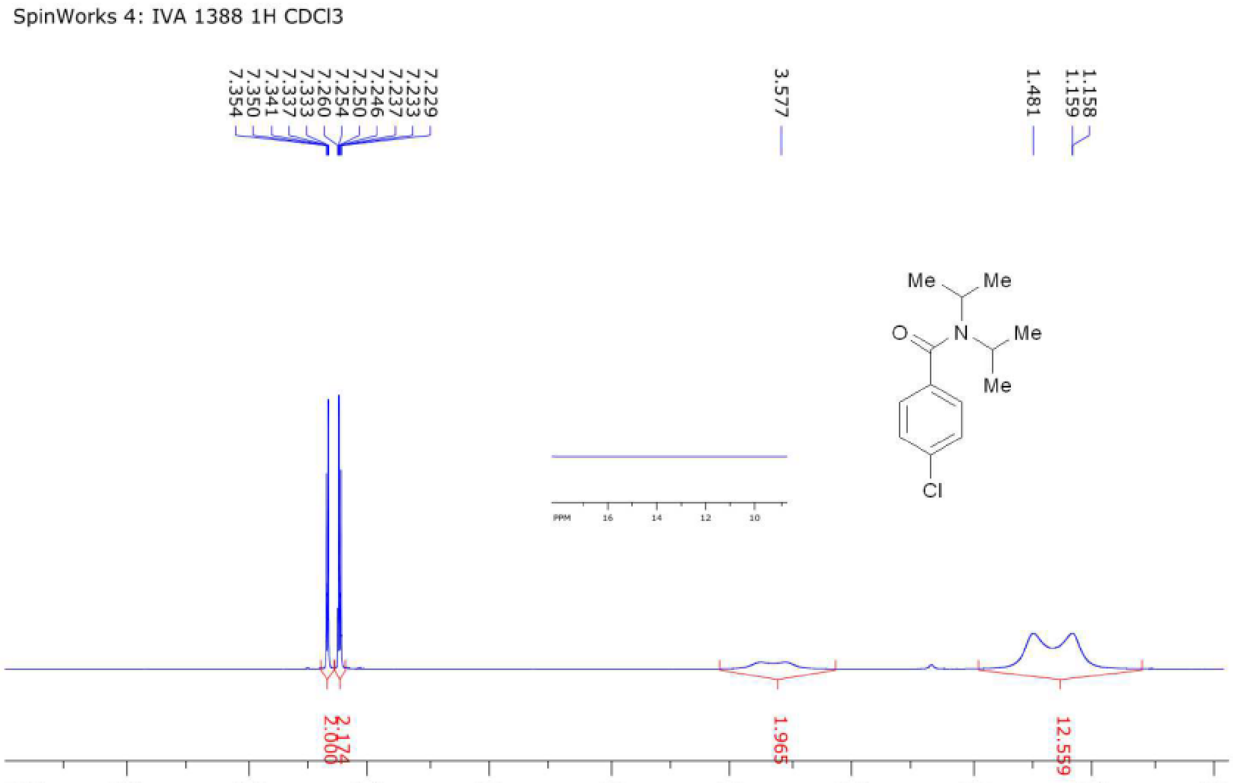

file: D:\NAPO\NMR\500-2\mkr11112\25\fid expt: <zg30>
transmitter freq.: 500.133001 MHz
time domain size: 65536 points
width: 12335.53 Hz = 24.6645 ppm = 0.188225 Hz/pt
number of scans: 24

freq. of 0 ppm: 500.130024 MHz
processed size: 65536 complex points
LB: 0.300 GF: 0.0000
Hz/cm: 202.574 ppm/cm: 0.40504

Compound 5p (NMR in $CDCl_3$)

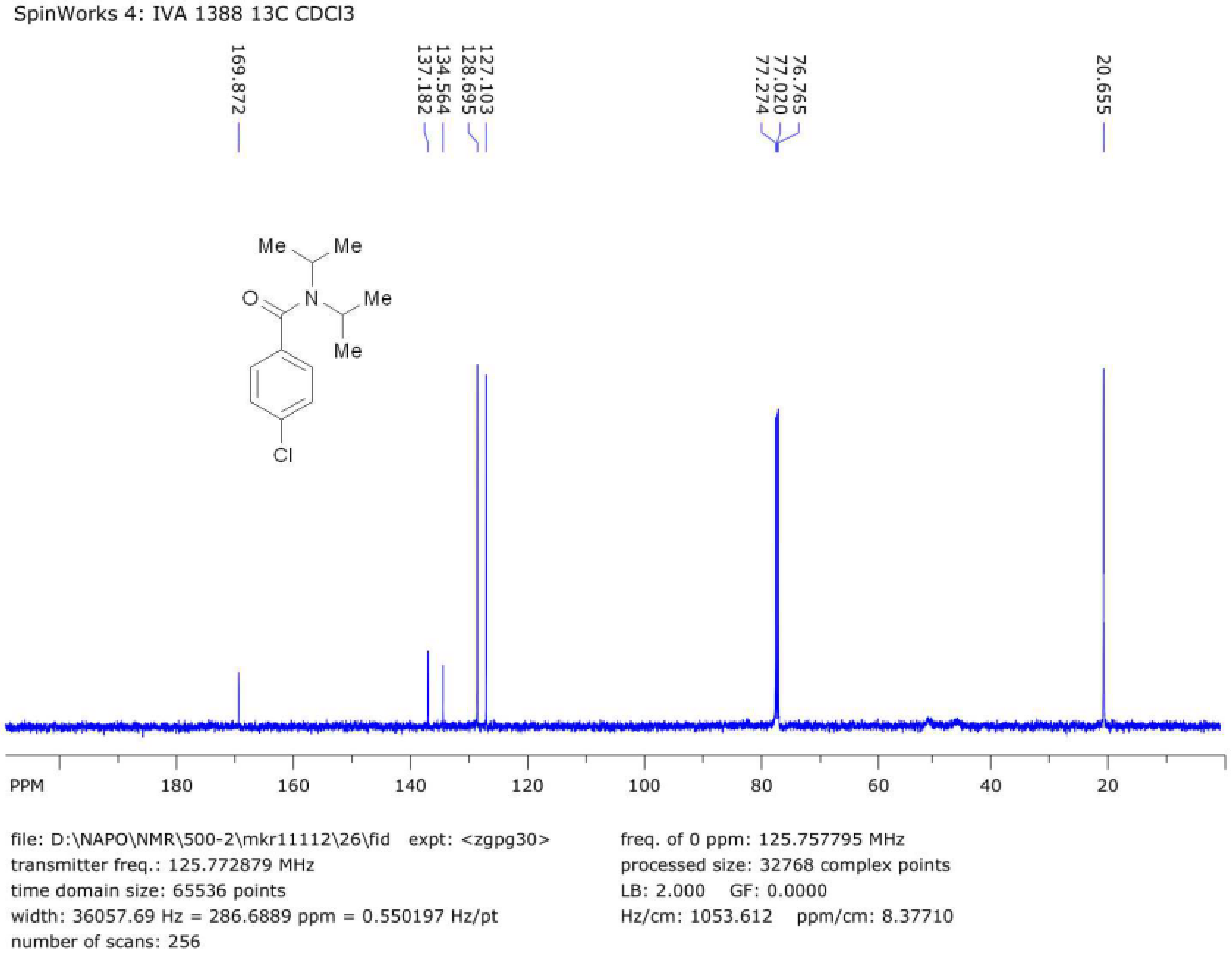

Compound 5q (NMR in $CDCl_3$)

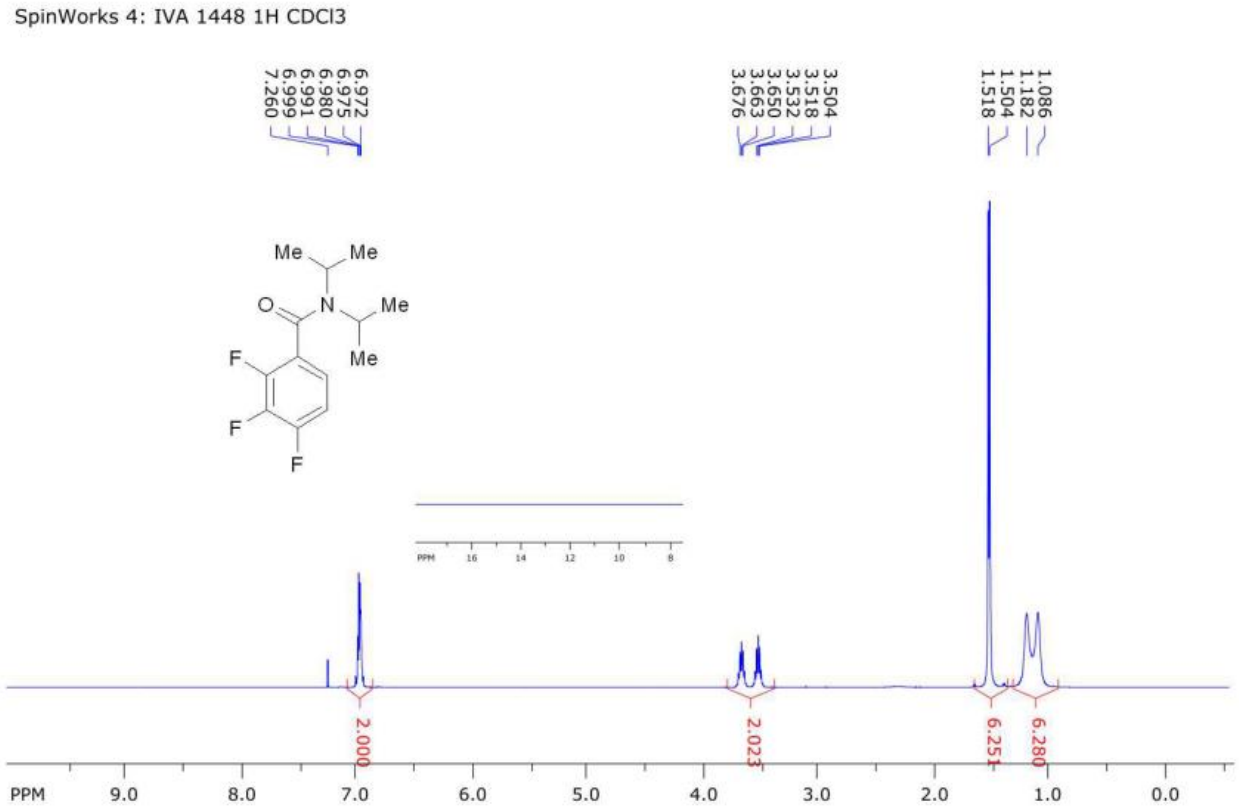

file: D:\NAPO\NMR\500-2\mkr11712\7\fid expt: <zg30>
transmitter freq.: 500.133001 MHz
time domain size: 65536 points
width: 12335.53 Hz = 24.6645 ppm = 0.188225 Hz/pt
number of scans: 24

freq. of 0 ppm: 500.130023 MHz
processed size: 65536 complex points
LB: 0.300 GF: 0.0000
Hz/cm: 212.760 ppm/cm: 0.42541

Compound 5q (NMR in $CDCl_3$)

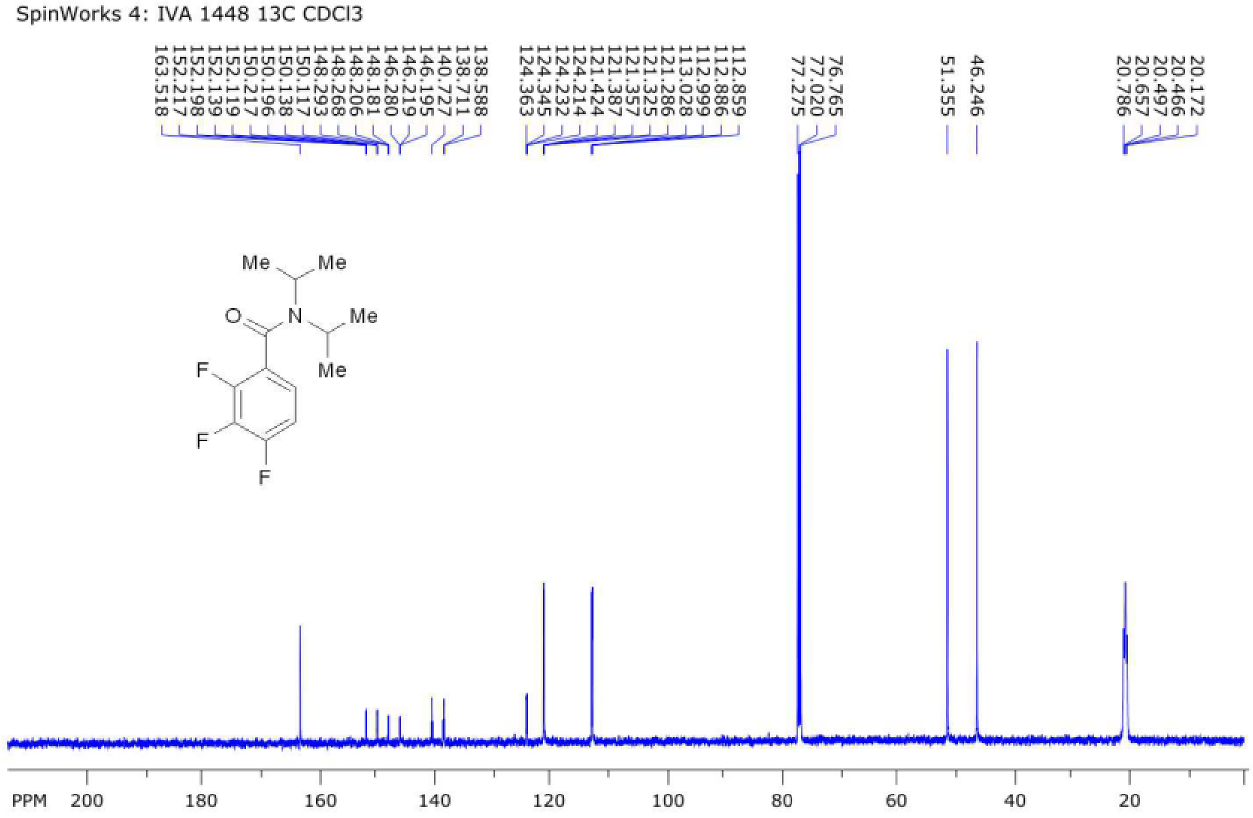

file: D:\NAPO\NMR\500-2\mkr11712\8\fid expt: <zgpg30>
transmitter freq.: 125.772879 MHz
time domain size: 65536 points
width: 36057.69 Hz = 286.6889 ppm = 0.550197 Hz/pt
number of scans: 1024

freq. of 0 ppm: 125.757795 MHz
processed size: 32768 complex points
LB: 2.000 GF: 0.0000
Hz/cm: 1078.423 ppm/cm: 8.57437

Compound 5r (NMR in $CDCl_3$)

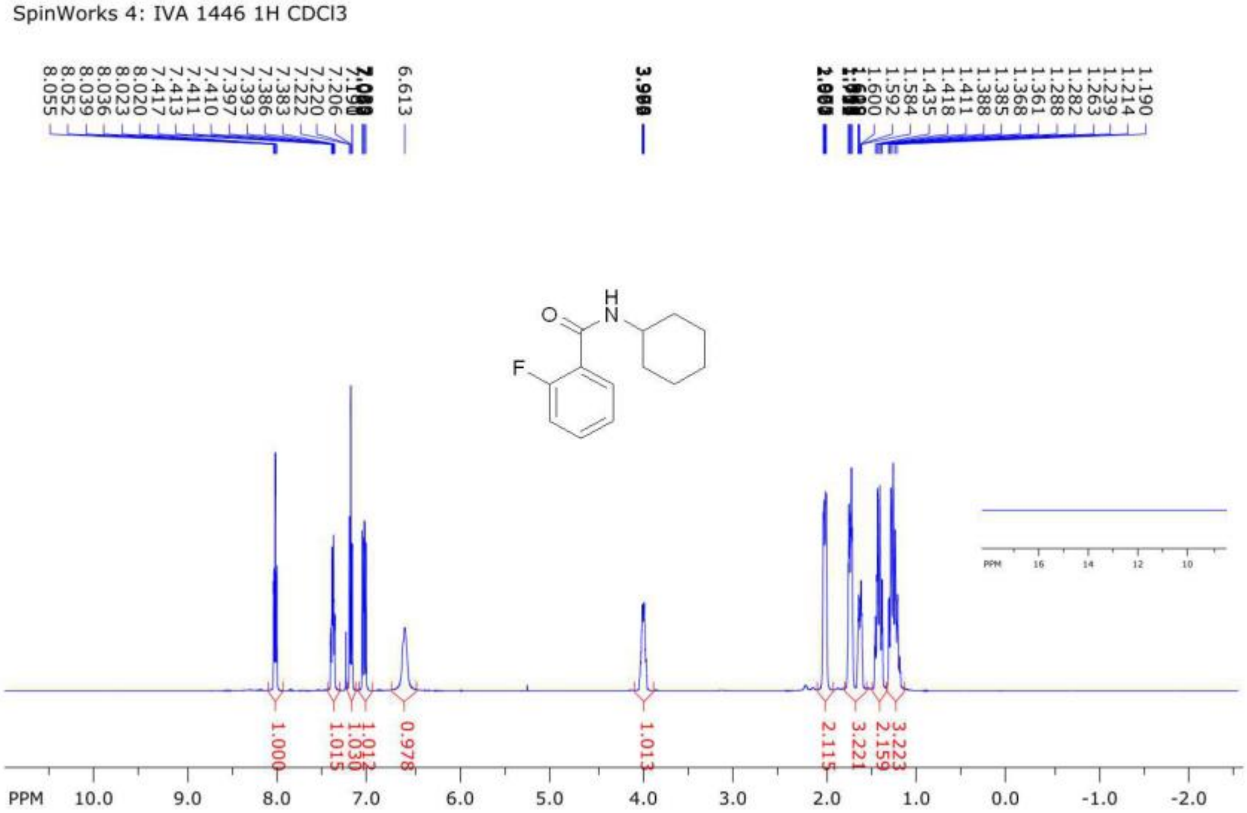

file: D:\NAPO\NMR\500-2\mkr11712\25\fid expt: <zg30>
transmitter freq.: 500.133001 MHz
time domain size: 65536 points
width: 12335.53 Hz = 24.6645 ppm = 0.188225 Hz/pt
number of scans: 24

freq. of 0 ppm: 500.130023 MHz
processed size: 65536 complex points
LB: 0.300 GF: 0.0000
Hz/cm: 271.608 ppm/cm: 0.54307

Compound 5r (NMR in $CDCl_3$)

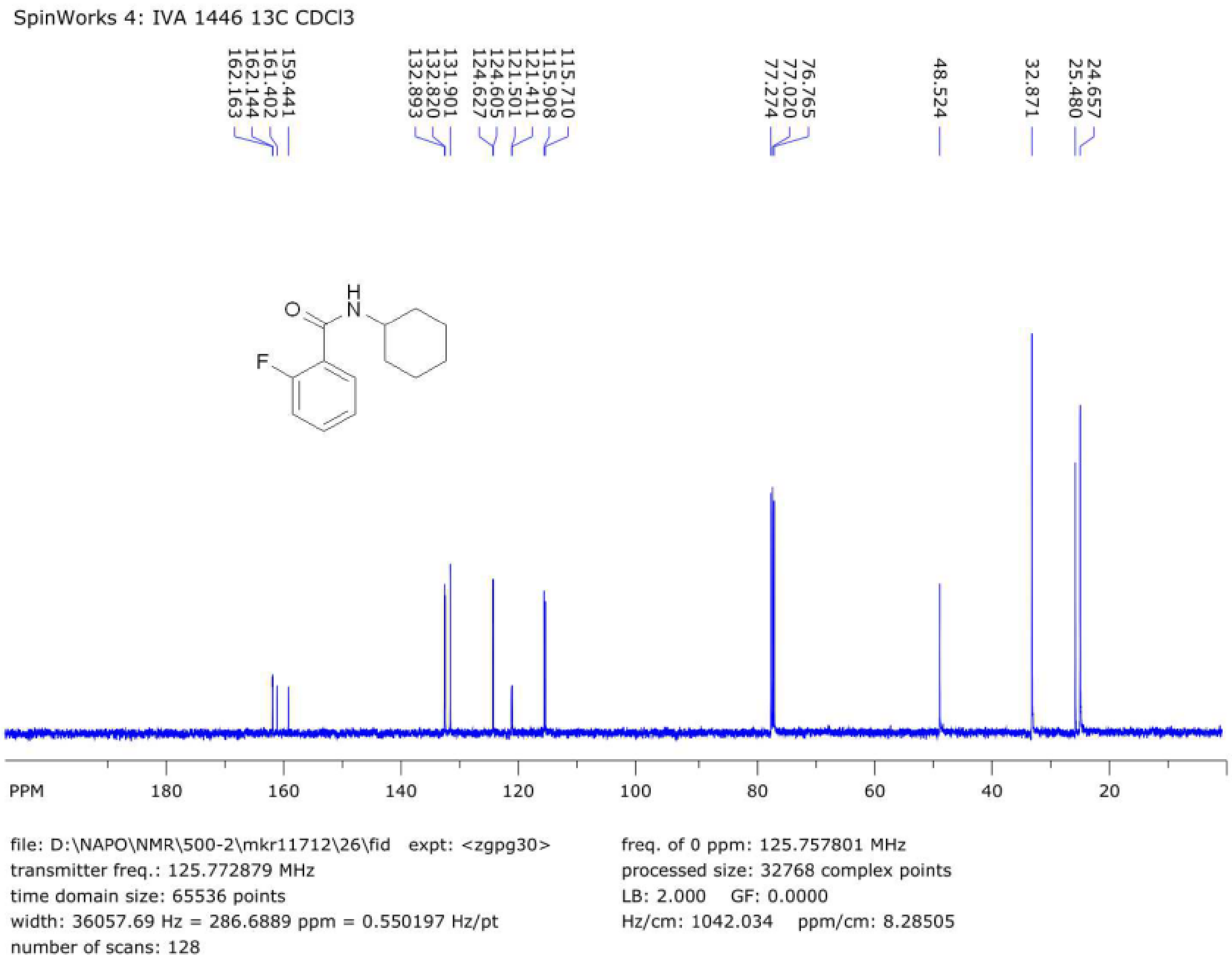

Compound 5s (NMR in $CDCl_3$)

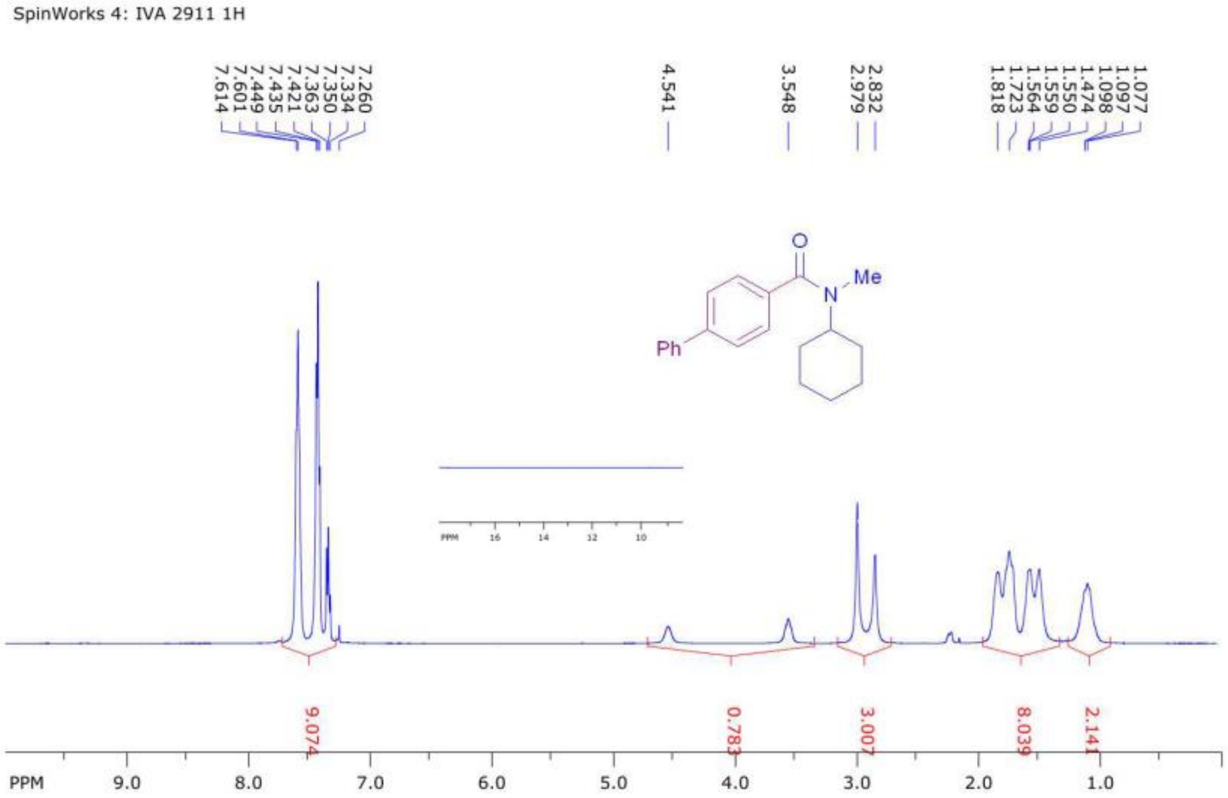

file: ...APO\NMR\500-2\mkr10606\11 2911\fid expt: <zg30>
transmitter freq.: 500.133001 MHz
time domain size: 65536 points
width: 12335.53 Hz = 24.6645 ppm = 0.188225 Hz/pt
number of scans: 24

freq. of 0 ppm: 500.130024 MHz
processed size: 65536 complex points
LB: 0.300 GF: 0.0000
Hz/cm: 200.316 ppm/cm: 0.40053

Compound 5s (NMR in $CDCl_3$)

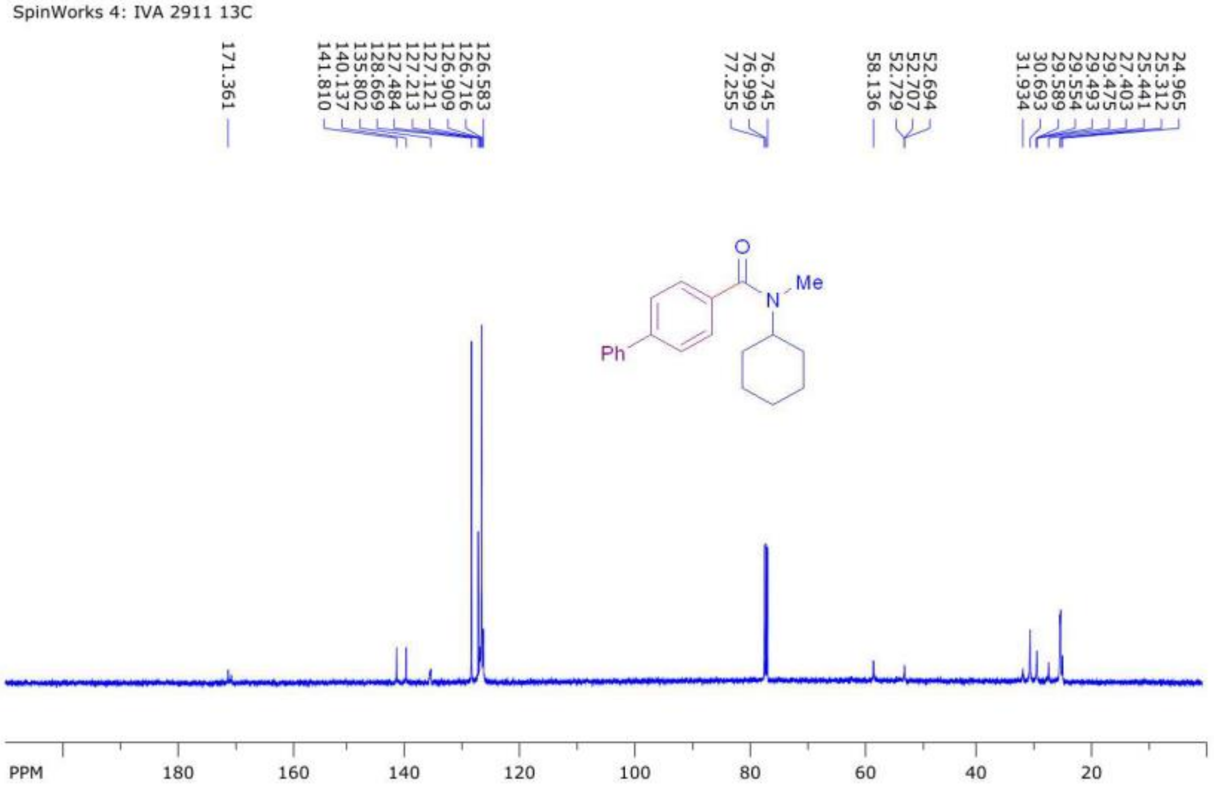

file: D:\NAPO\NMR\500-2\mkr10606\12\fid expt: <zgpg30>
transmitter freq.: 125.772879 MHz
time domain size: 65536 points
width: 36057.69 Hz = 286.6889 ppm = 0.550197 Hz/pt
number of scans: 512

freq. of 0 ppm: 125.757818 MHz
processed size: 32768 complex points
LB: 2.000 GF: 0.0000
Hz/cm: 1056.203 ppm/cm: 8.39770

Compound 5t (NMR in CDCl$_3$)

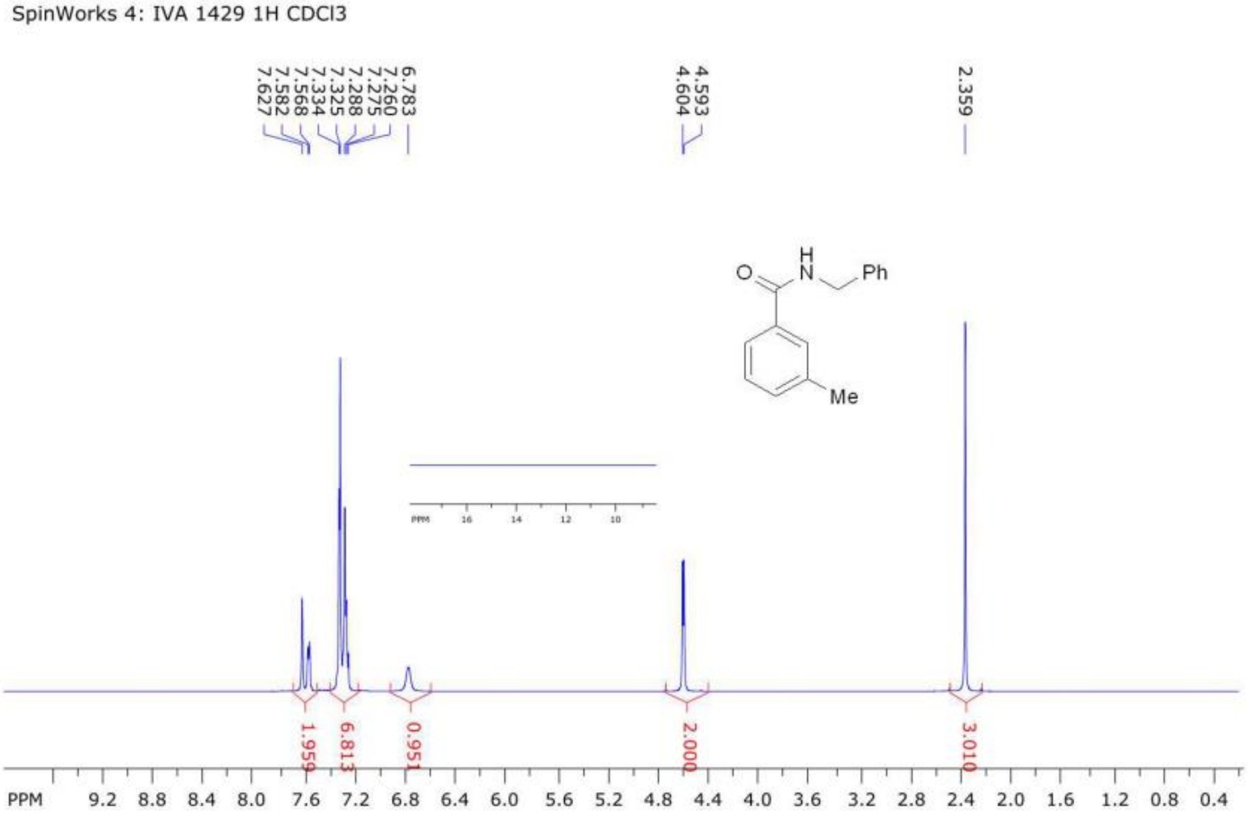

file: D:\NAPO\NMR\500-2\mkr11712\13\fid expt: <zg30>
transmitter freq.: 500.133001 MHz
time domain size: 65536 points
width: 12335.53 Hz = 24.6645 ppm = 0.188225 Hz/pt
number of scans: 24

freq. of 0 ppm: 500.130023 MHz
processed size: 65536 complex points
LB: 0.300 GF: 0.0000
Hz/cm: 196.350 ppm/cm: 0.39260

Compound 5t (NMR in $CDCl_3$)

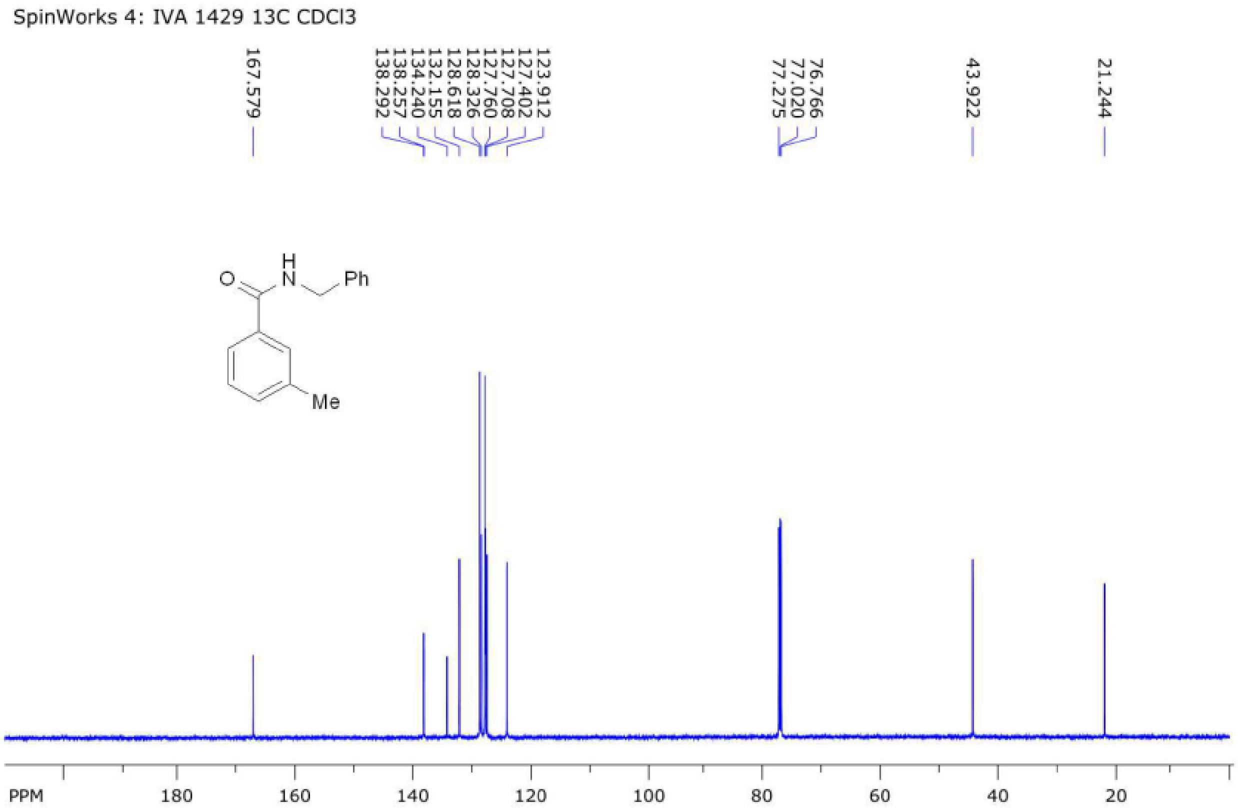

file: D:\NAPO\NMR\500-2\mkr11712\14\fid expt: <zgpg30>
transmitter freq.: 125.772879 MHz
time domain size: 65536 points
width: 36057.69 Hz = 286.6889 ppm = 0.550197 Hz/pt
number of scans: 1024

freq. of 0 ppm: 125.757805 MHz
processed size: 32768 complex points
LB: 2.000 GF: 0.0000
Hz/cm: 1058.574 ppm/cm: 8.41656

Compound 5u (NMR in $CDCl_3$)

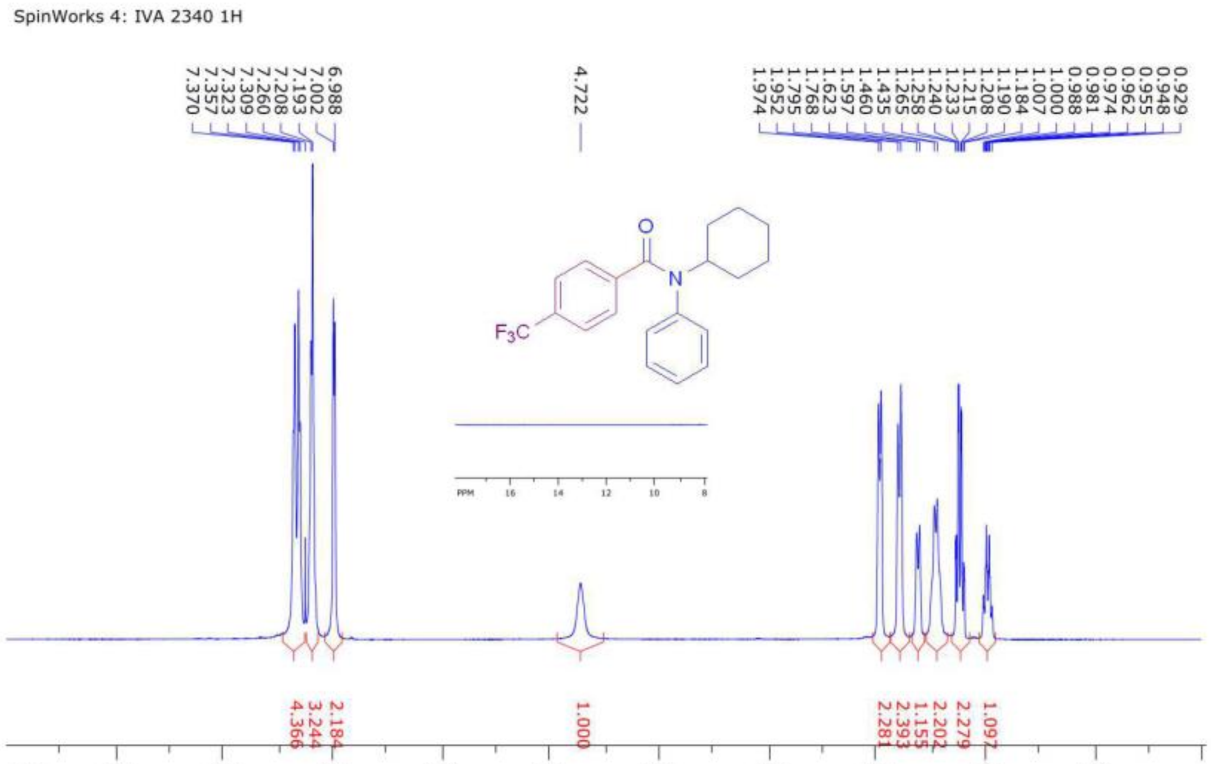

file: D:\NAPO\NMR\500-2\mkr10904\1\fid expt: <zg30>
transmitter freq.: 500.133001 MHz
time domain size: 65536 points
width: 12335.53 Hz = 24.6645 ppm = 0.188225 Hz/pt
number of scans: 24

freq. of 0 ppm: 500.130023 MHz
processed size: 65536 complex points
LB: 0.300 GF: 0.0000
Hz/cm: 220.511 ppm/cm: 0.44091

Compound 5u (NMR in $CDCl_3$)

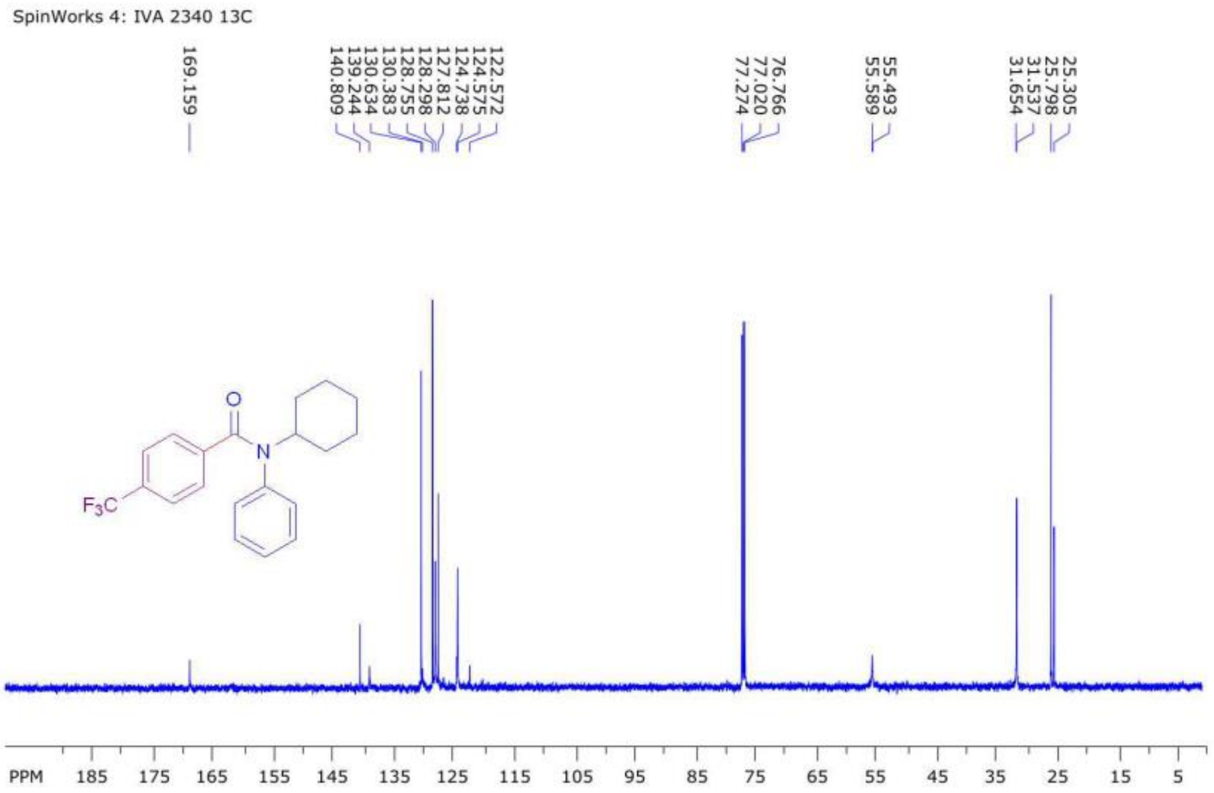

file: D:\NAPO\NMR\500-2\mkr10904\2\fid expt: <zgpg30>
transmitter freq.: 125.772879 MHz
time domain size: 65536 points
width: 36057.69 Hz = 286.6889 ppm = 0.550197 Hz/pt
number of scans: 512

freq. of 0 ppm: 125.757795 MHz
processed size: 32768 complex points
LB: 2.000 GF: 0.0000
Hz/cm: 1001.957 ppm/cm: 7.96640

Compound 5v (NMR in $CDCl_3$)

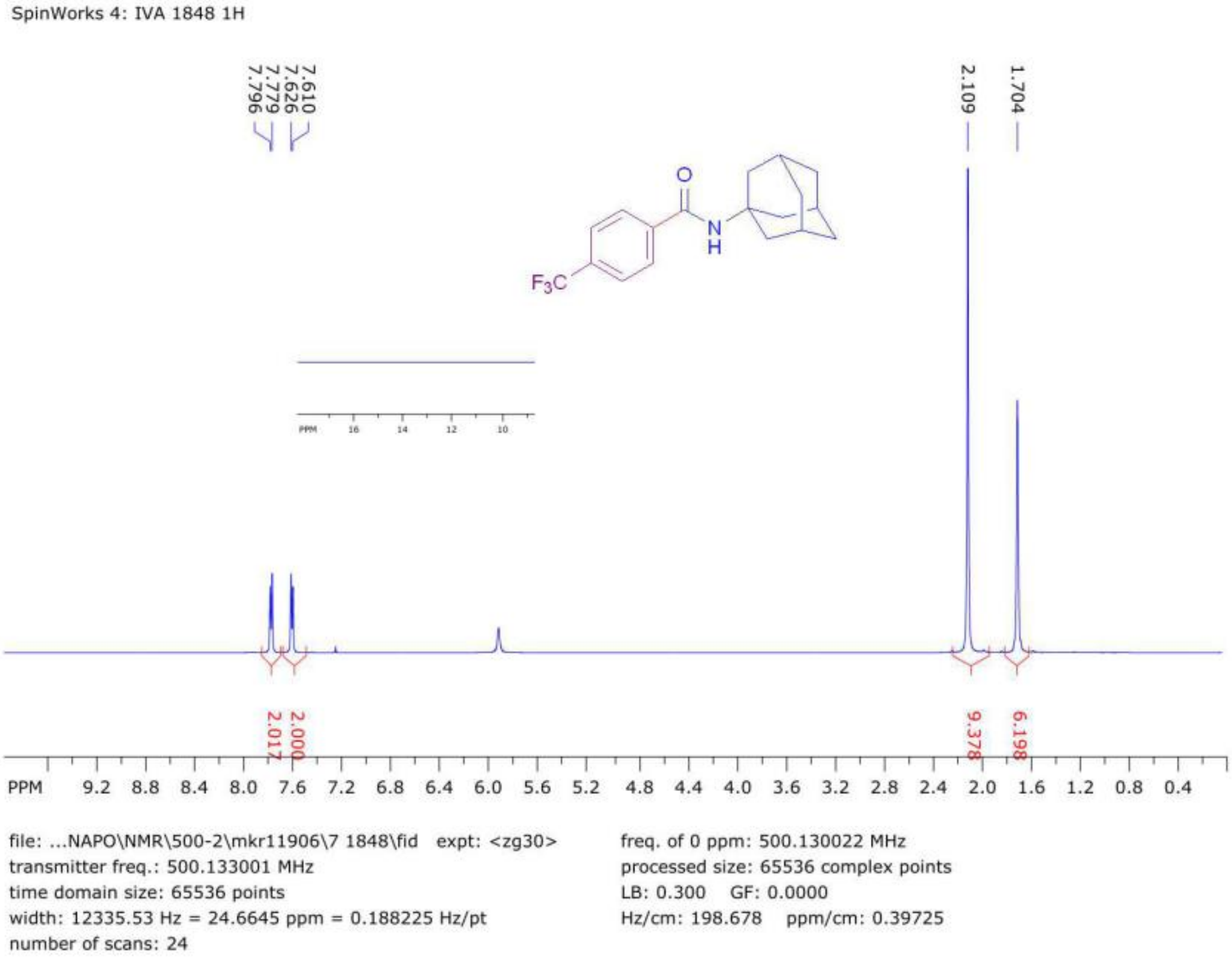

Compound 5v (NMR in $CDCl_3$)

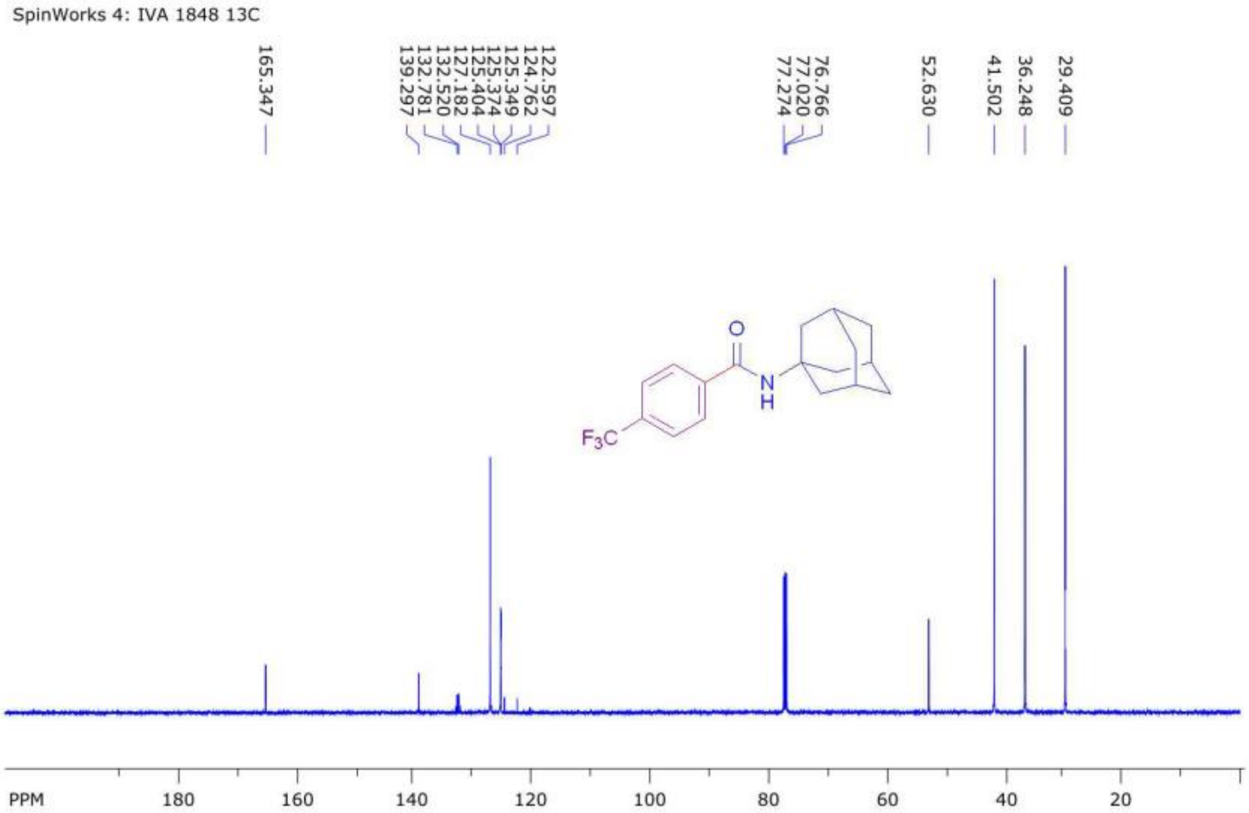

file: D:\NAPO\NMR\500-2\mkr11906\8\fid expt: <zgpg30>
transmitter freq.: 125.772879 MHz
time domain size: 65536 points
width: 36057.69 Hz = 286.6889 ppm = 0.550197 Hz/pt
number of scans: 512

freq. of 0 ppm: 125.757796 MHz
processed size: 32768 complex points
LB: 2.000 GF: 0.0000
Hz/cm: 1056.203 ppm/cm: 8.39770

Compound 5w (NMR in $CDCl_3$)

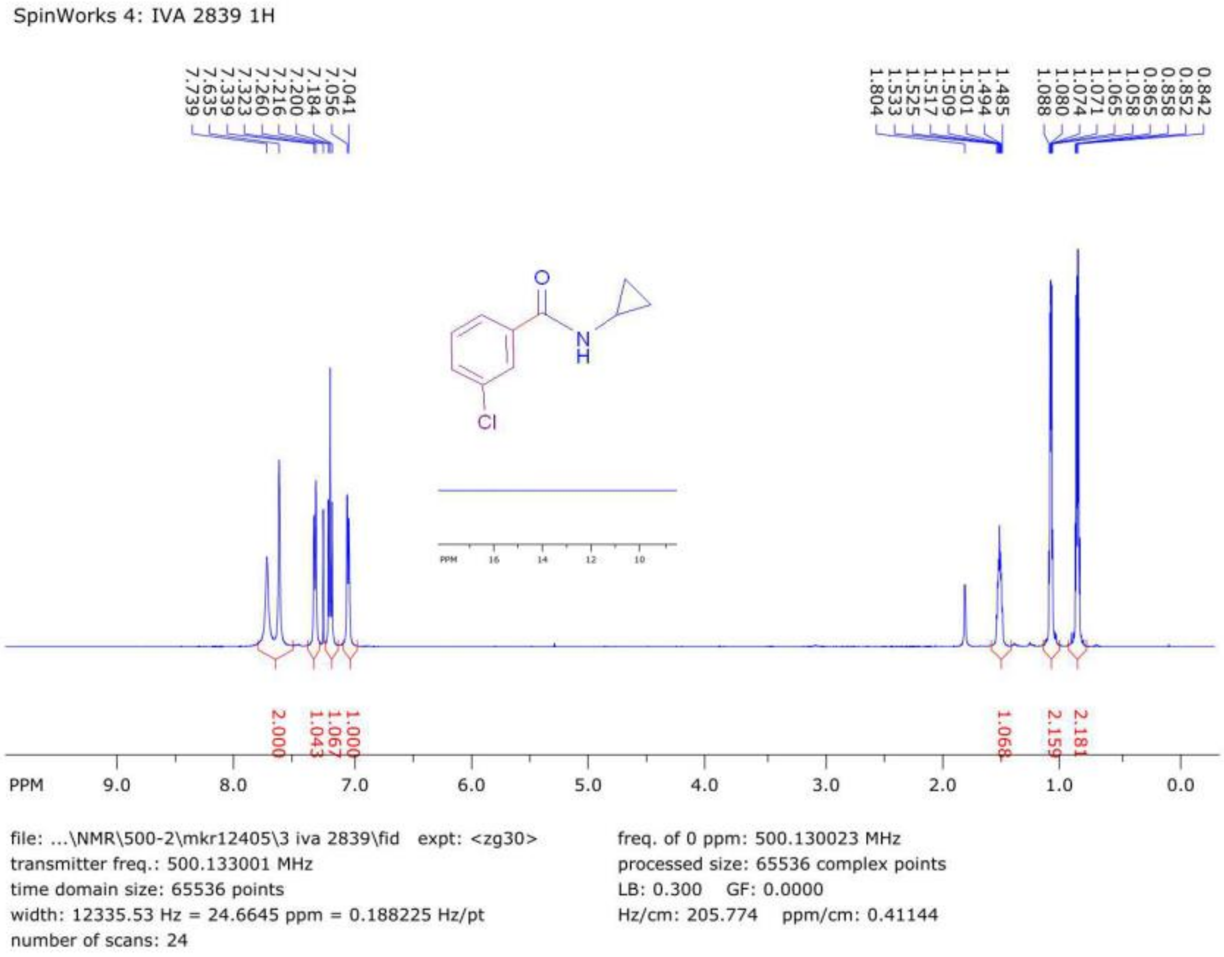

Compound 5w (NMR in $CDCl_3$)

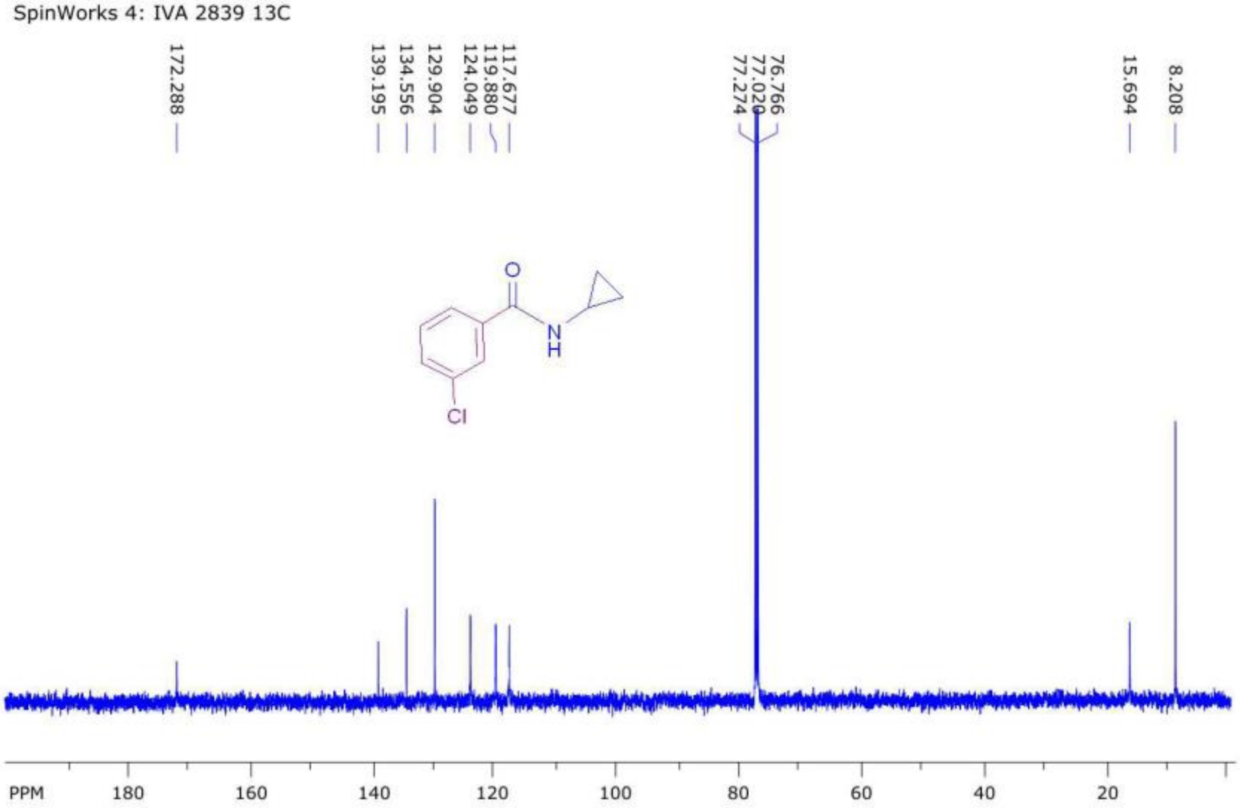

file: D:\NAPO\NMR\500-2\mkr12405\4\fid expt: <zgpg30>
transmitter freq.: 125.772879 MHz
time domain size: 65536 points
width: 36057.69 Hz = 286.6889 ppm = 0.550197 Hz/pt
number of scans: 256

freq. of 0 ppm: 125.757794 MHz
processed size: 32768 complex points
LB: 2.000 GF: 0.0000
Hz/cm: 1013.125 ppm/cm: 8.05520

Compound 5x (NMR in $CDCl_3$)

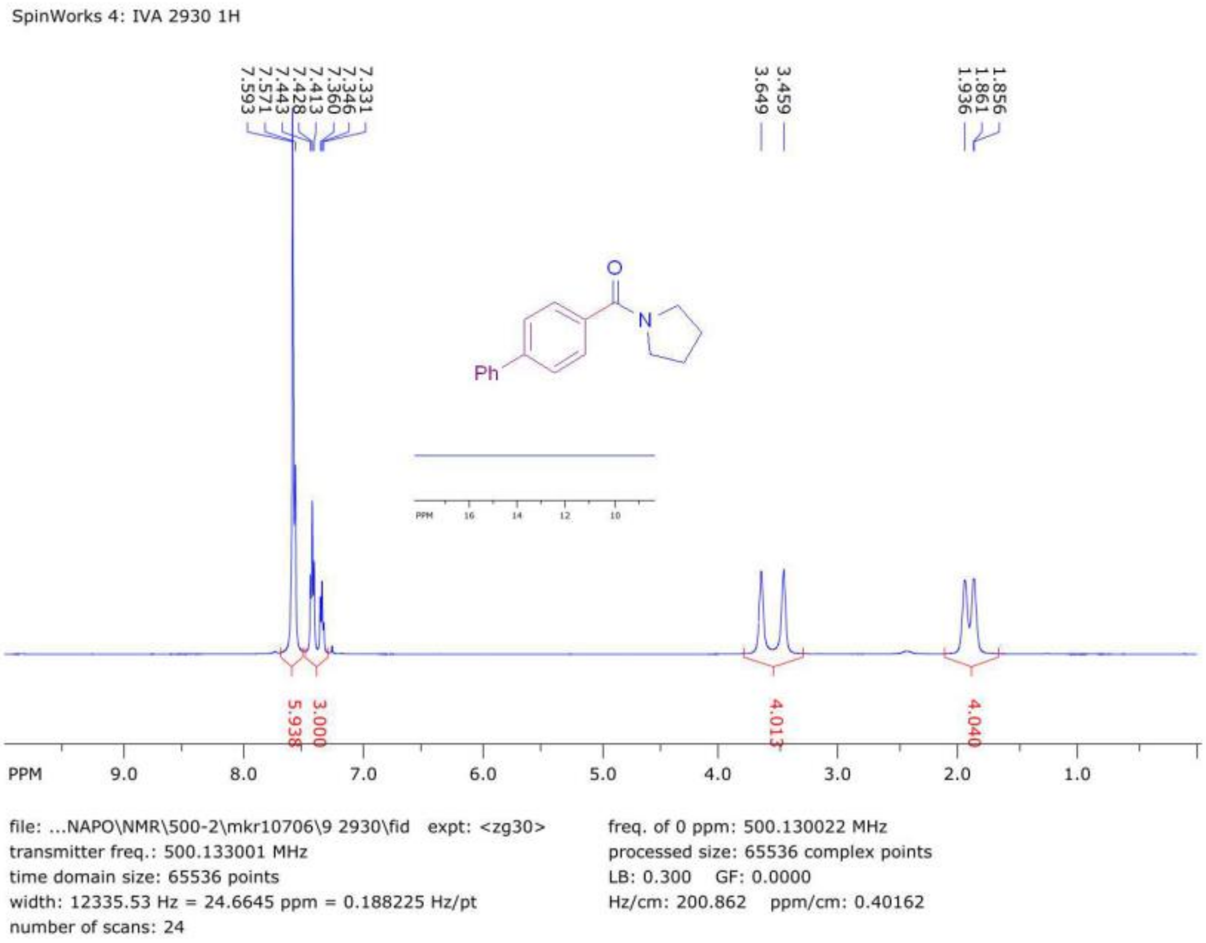

Compound 5x (NMR in $CDCl_3$)

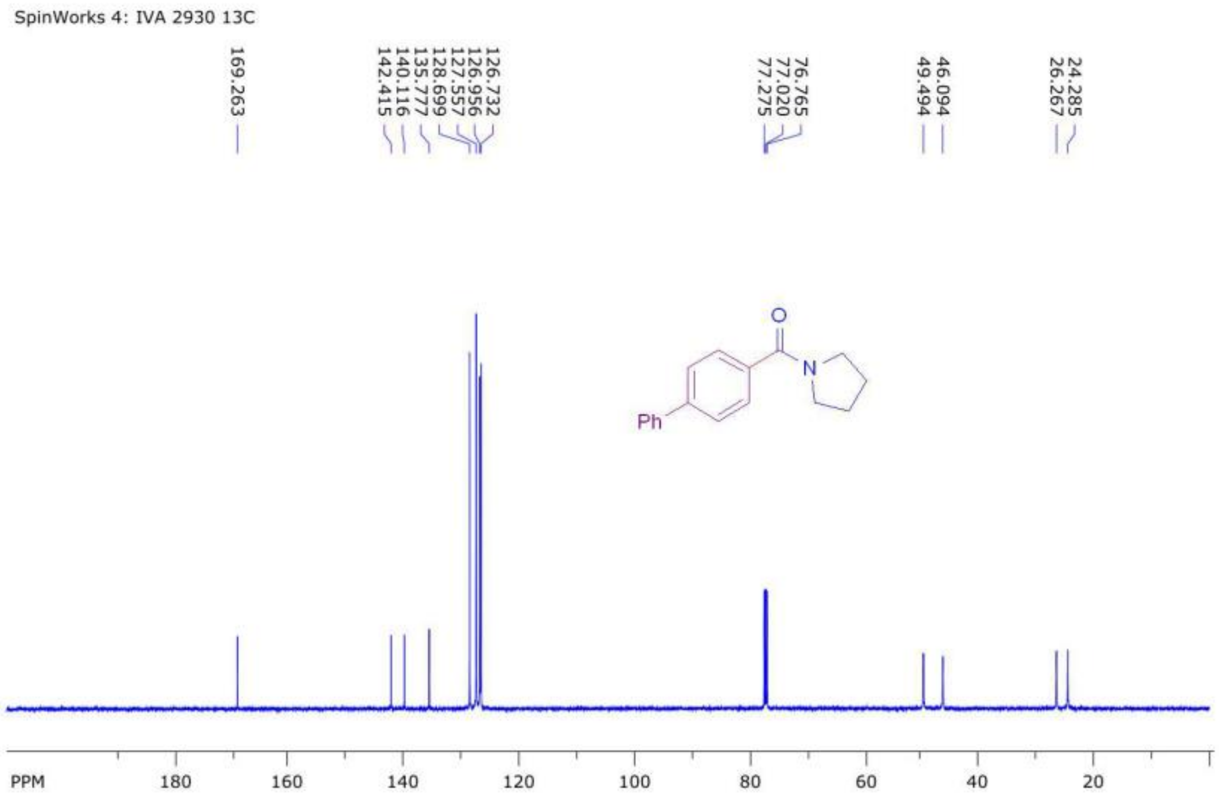

file: D:\NAPO\NMR\500-2\mkr10706\10\fid expt: <zgpg30>
transmitter freq.: 125.772879 MHz
time domain size: 65536 points
width: 36057.69 Hz = 286.6889 ppm = 0.550197 Hz/pt
number of scans: 512

freq. of 0 ppm: 125.757814 MHz
processed size: 32768 complex points
LB: 2.000 GF: 0.0000
Hz/cm: 1056.203 ppm/cm: 8.39770

Compound 5y (NMR in CDCl$_3$)

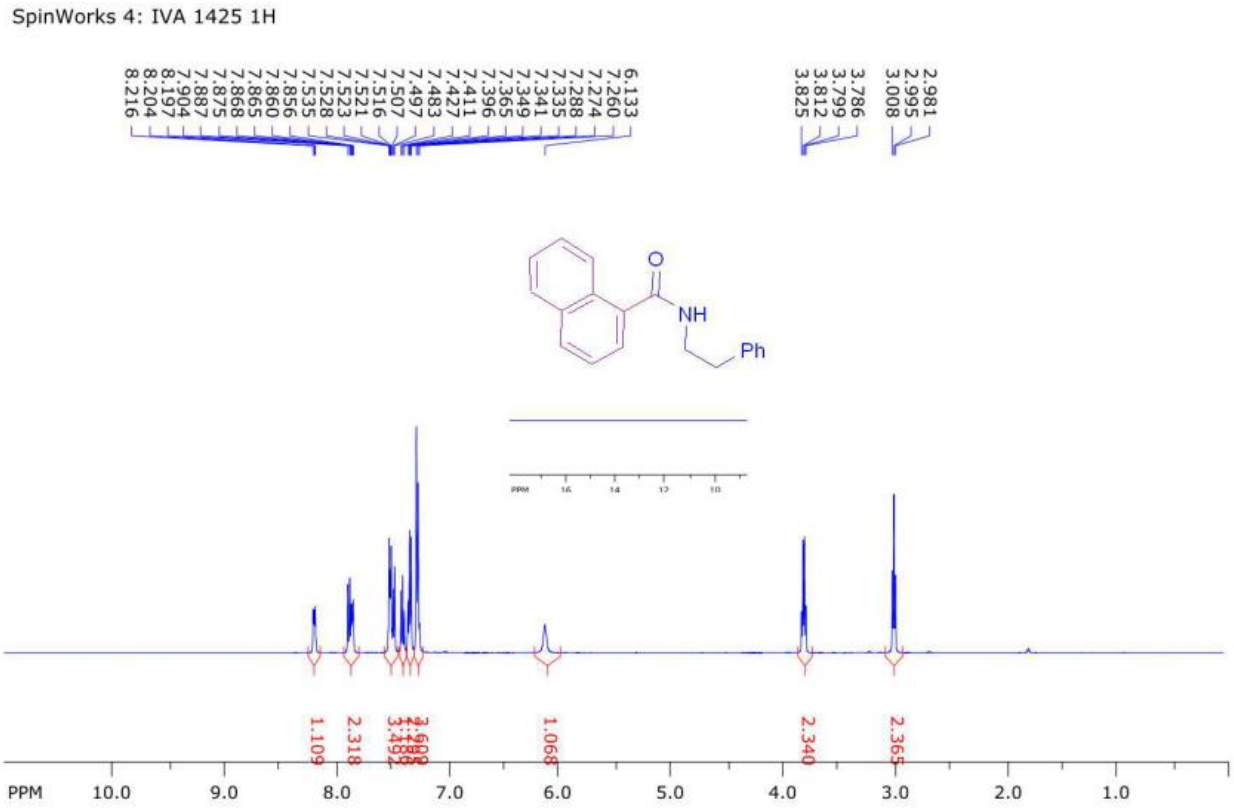

file: D:\NAPO\NMR\500-2\mkr100201\7\fid expt: <zg30>
transmitter freq.: 500.133001 MHz
time domain size: 65536 points
width: 12335.53 Hz = 24.6645 ppm = 0.188225 Hz/pt
number of scans: 24

freq. of 0 ppm: 500.130011 MHz
processed size: 65536 complex points
LB: 0.300 GF: 0.0000
Hz/cm: 219.420 ppm/cm: 0.43872

Compound 5y (NMR in $CDCl_3$)

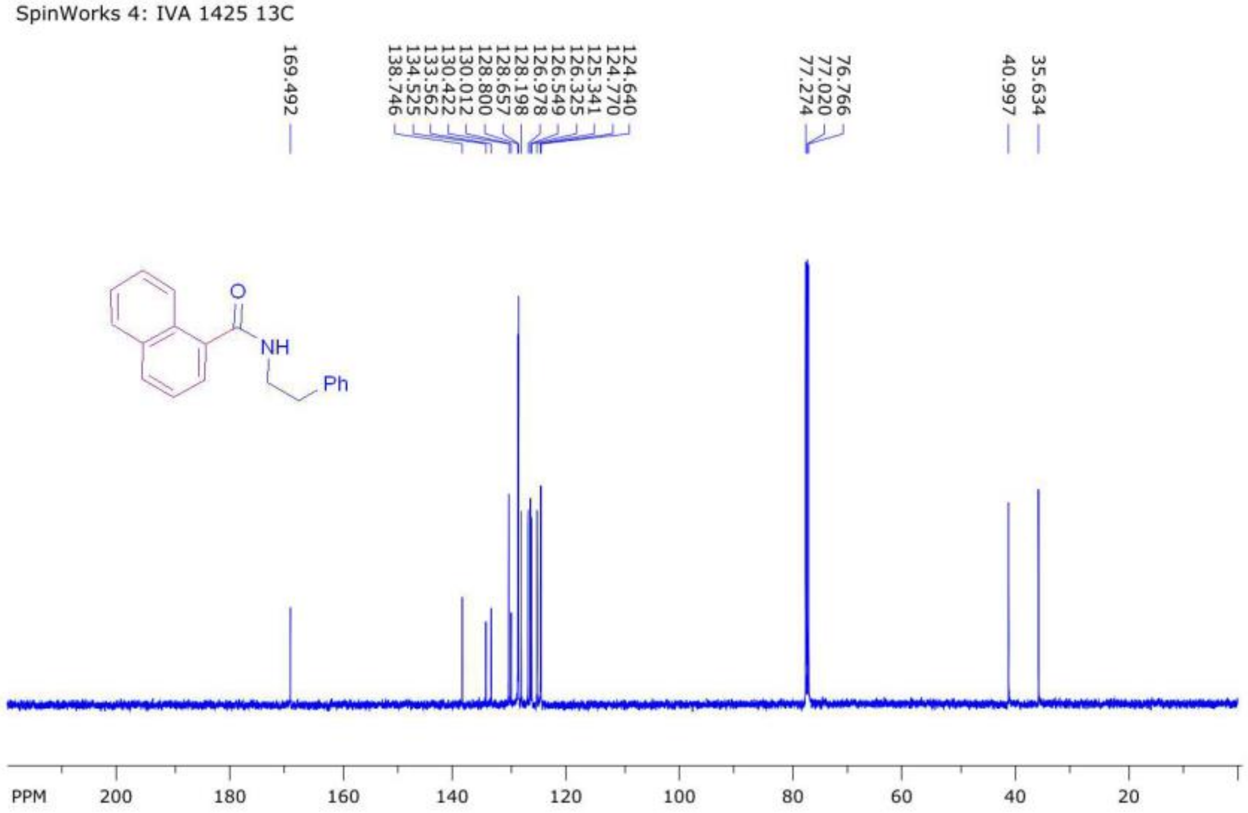

file: D:\NAPO\NMR\500-2\mkr100201\8\fid expt: <zgpg30>
transmitter freq.: 125.772879 MHz
time domain size: 65536 points
width: 36057.69 Hz = 286.6889 ppm = 0.550197 Hz/pt
number of scans: 512

freq. of 0 ppm: 125.757800 MHz
processed size: 32768 complex points
LB: 2.000 GF: 0.0000
Hz/cm: 1107.258 ppm/cm: 8.80363

Compound 5z (NMR in $CDCl_3$)

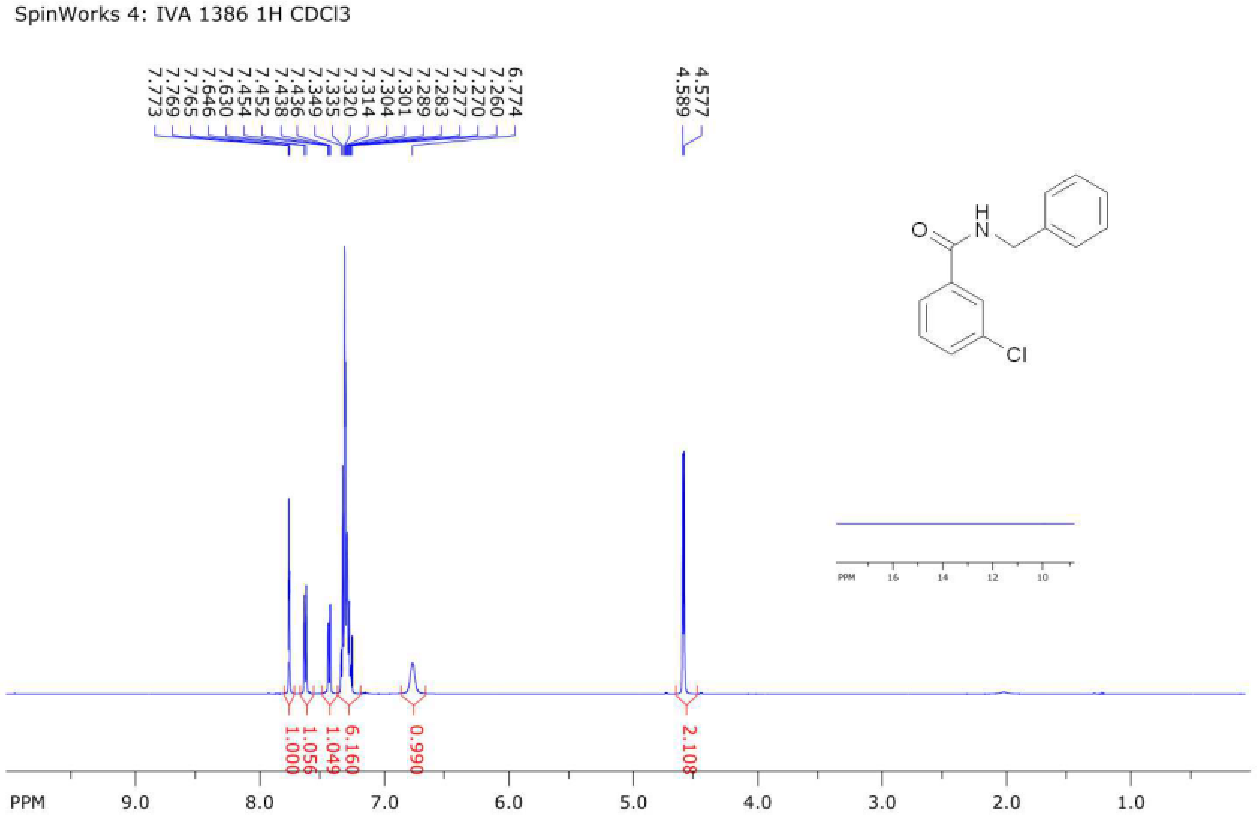

file: D:\NAPO\NMR\500-2\mkr11112\23\fid expt: <zg30>
transmitter freq.: 500.133001 MHz
time domain size: 65536 points
width: 12335.53 Hz = 24.6645 ppm = 0.188225 Hz/pt
number of scans: 24

freq. of 0 ppm: 500.130023 MHz
processed size: 65536 complex points
LB: 0.300 GF: 0.0000
Hz/cm: 200.311 ppm/cm: 0.40052

Compound 5z (NMR in $CDCl_3$)

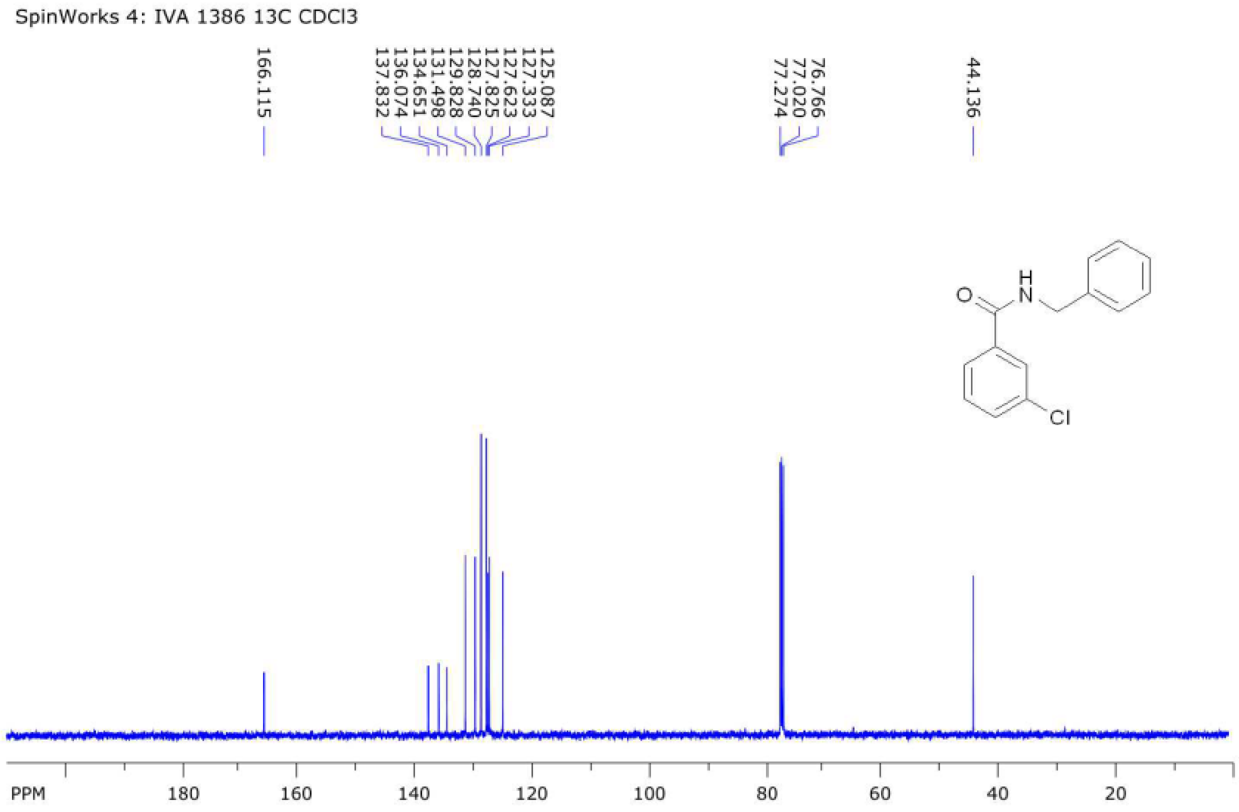

file: D:\NAPO\NMR\500-2\mkr11112\24\fid expt: <zgpg30>
transmitter freq.: 125.772879 MHz
time domain size: 65536 points
width: 36057.69 Hz = 286.6889 ppm = 0.550197 Hz/pt
number of scans: 256

freq. of 0 ppm: 125.757799 MHz
processed size: 32768 complex points
LB: 2.000 GF: 0.0000
Hz/cm: 1056.920 ppm/cm: 8.40340

Compound 5aa (NMR in $CDCl_3$)

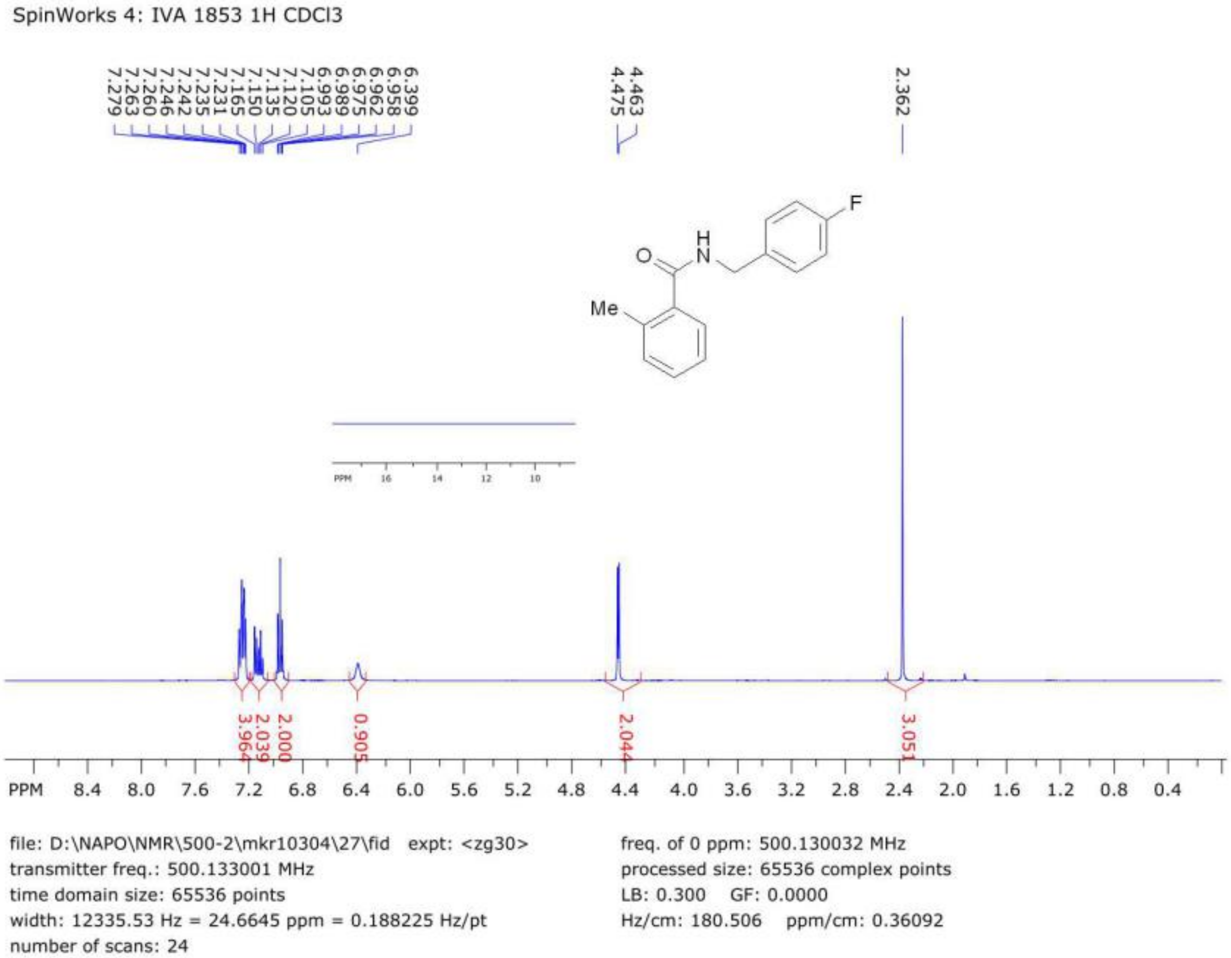

Compound 5aa (NMR in $CDCl_3$)

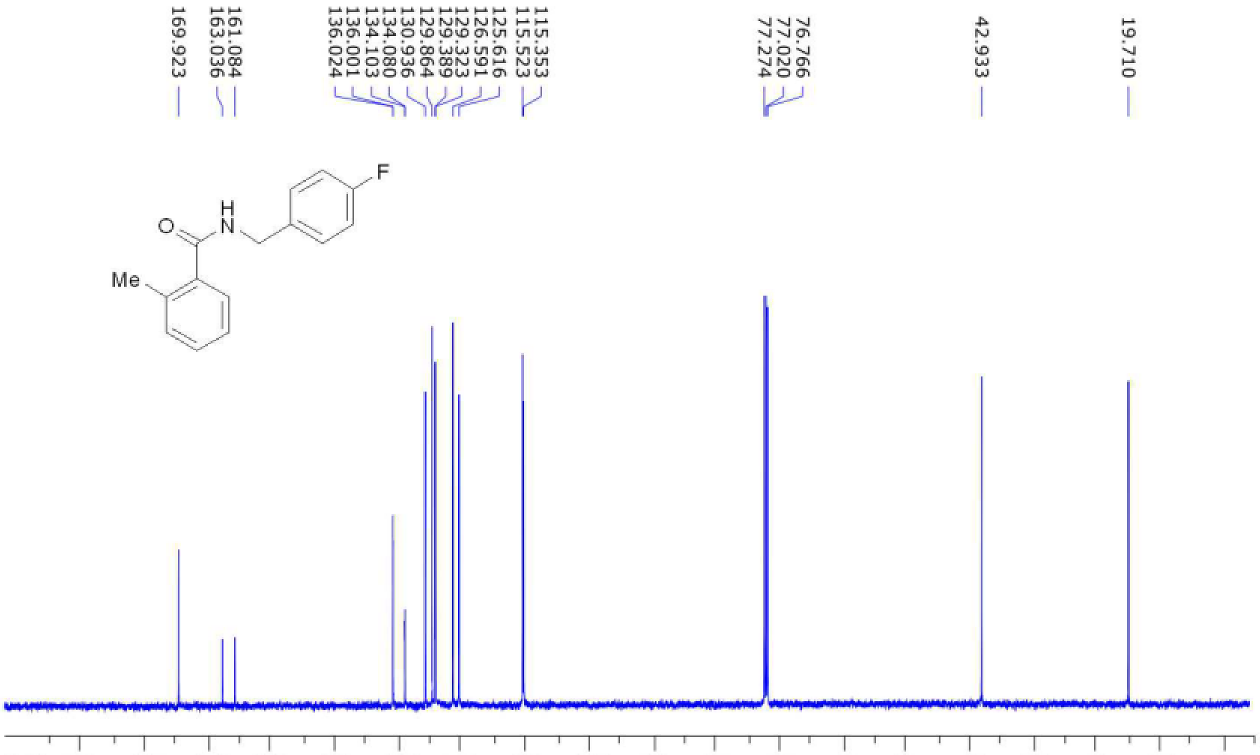

file: D:\NAPO\NMR\500-2\mkr10304\28\fid expt: <zgpg30>
transmitter freq.: 125.772879 MHz
time domain size: 65536 points
width: 36057.69 Hz = 286.6889 ppm = 0.550197 Hz/pt
number of scans: 512

freq. of 0 ppm: 125.757802 MHz
processed size: 32768 complex points
LB: 2.000 GF: 0.0000
Hz/cm: 990.760 ppm/cm: 7.87737

Compound 5ab (NMR in $CDCl_3$)

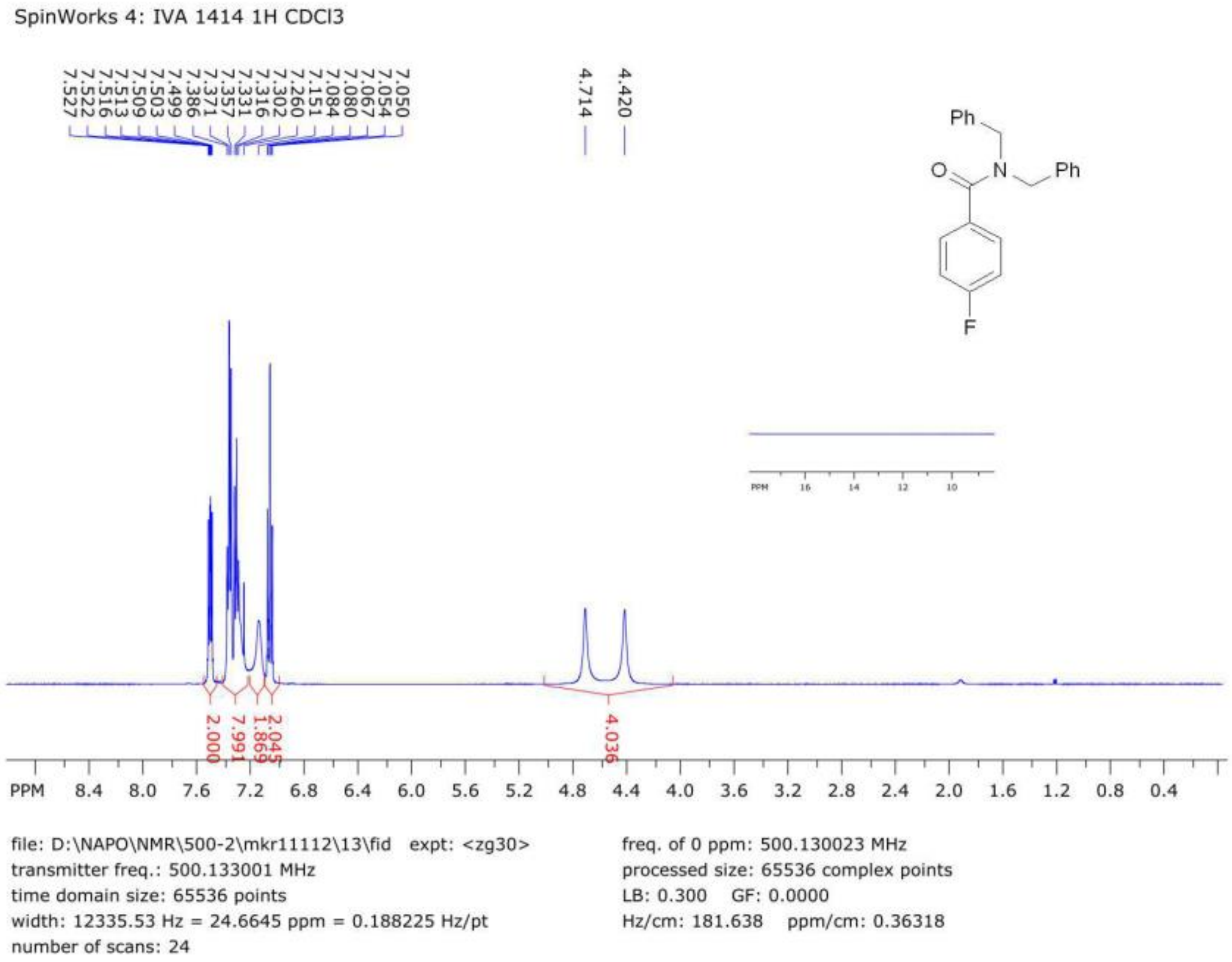

Compound 5ab (NMR in $CDCl_3$)

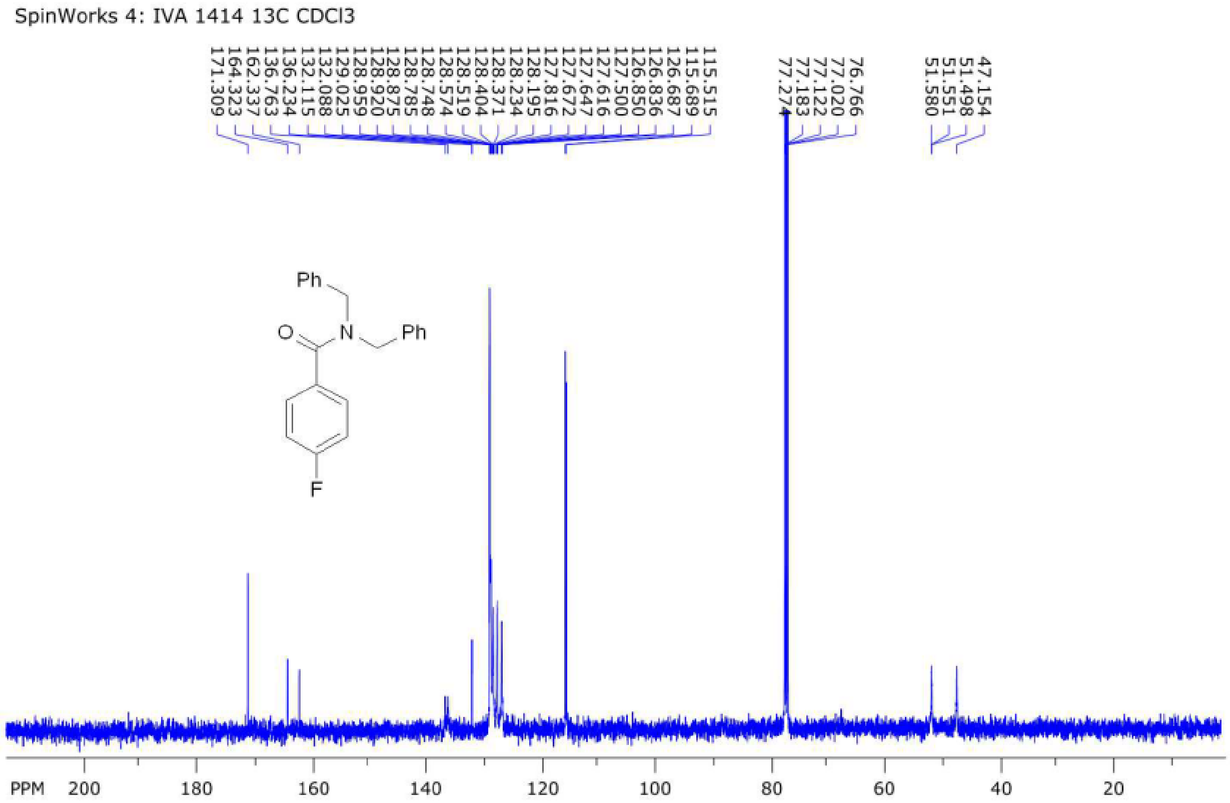

file: D:\NAPO\NMR\500-2\mkr11112\14\fid expt: <zgpg30>
transmitter freq.: 125.772879 MHz
time domain size: 65536 points
width: 36057.69 Hz = 286.6889 ppm = 0.550197 Hz/pt
number of scans: 256

freq. of 0 ppm: 125.757798 MHz
processed size: 32768 complex points
LB: 2.000 GF: 0.0000
Hz/cm: 1070.153 ppm/cm: 8.50861

Compound 5ac (NMR in $CDCl_3$)

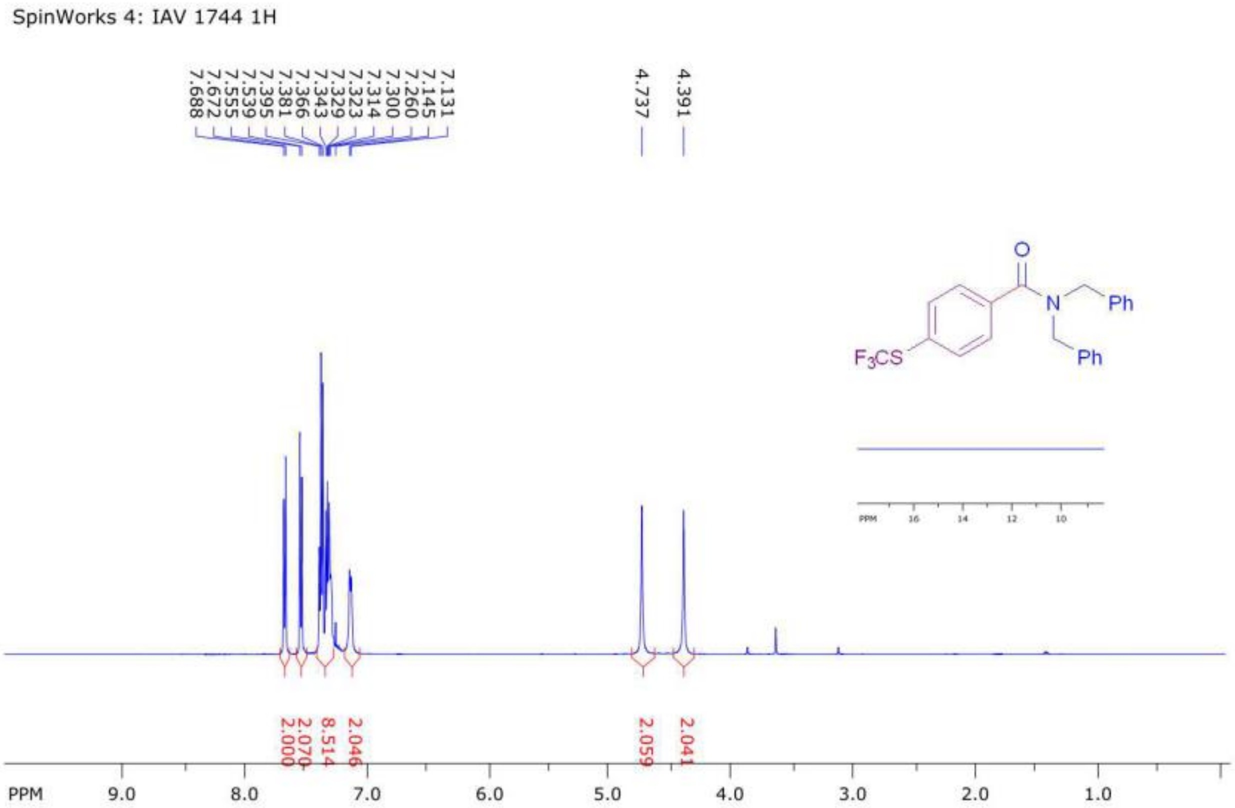

file: D:\NAPO\NMR\500-2\mkr21801\7\fid expt: <zg30>
transmitter freq.: 500.133001 MHz
time domain size: 65536 points
width: 12335.53 Hz = 24.6645 ppm = 0.188225 Hz/pt
number of scans: 24

freq. of 0 ppm: 500.130023 MHz
processed size: 65536 complex points
LB: 0.300 GF: 0.0000
Hz/cm: 201.407 ppm/cm: 0.40271

Compound 5ac (NMR in $CDCl_3$)

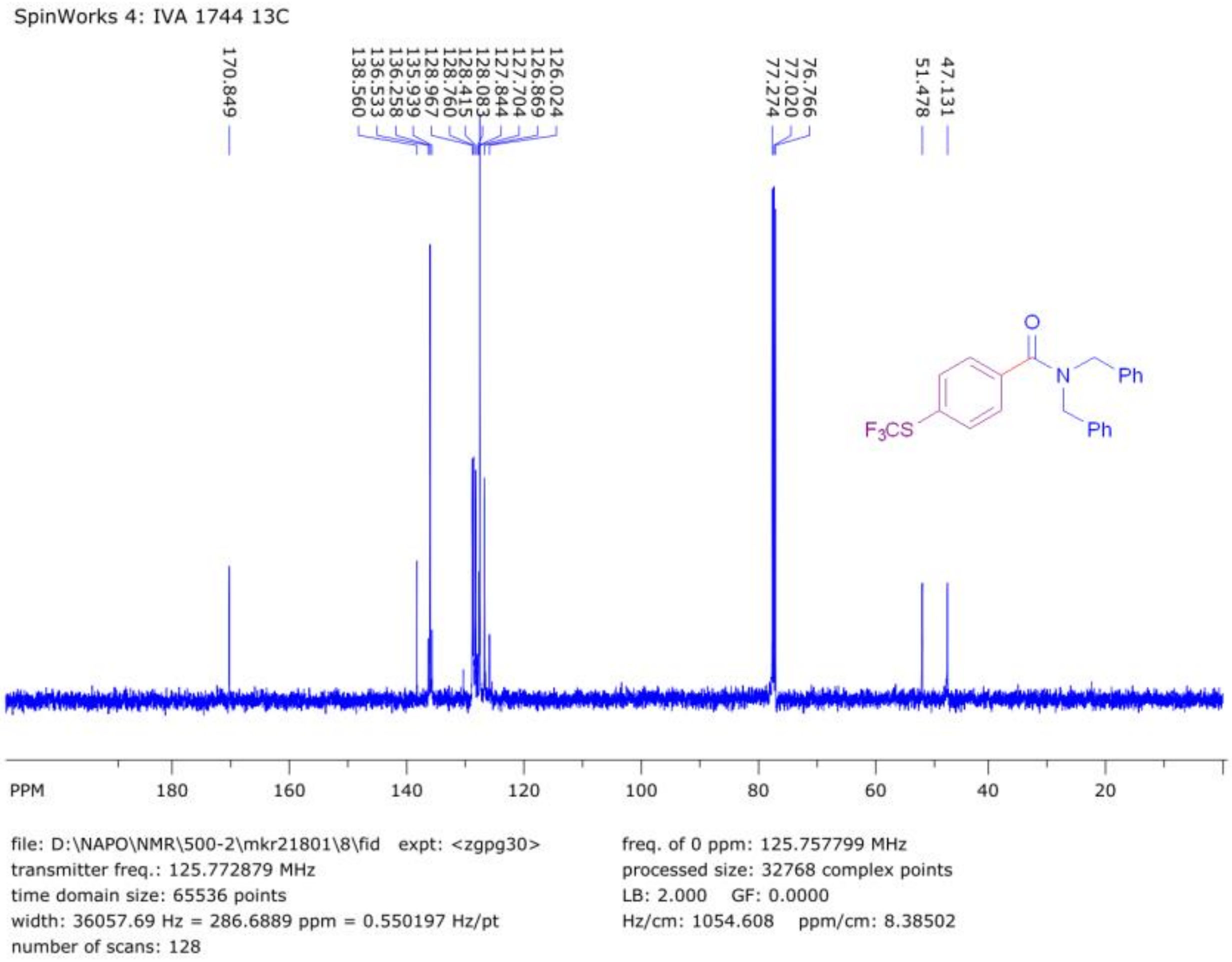

Compound 5ad (NMR in CDCl$_3$)

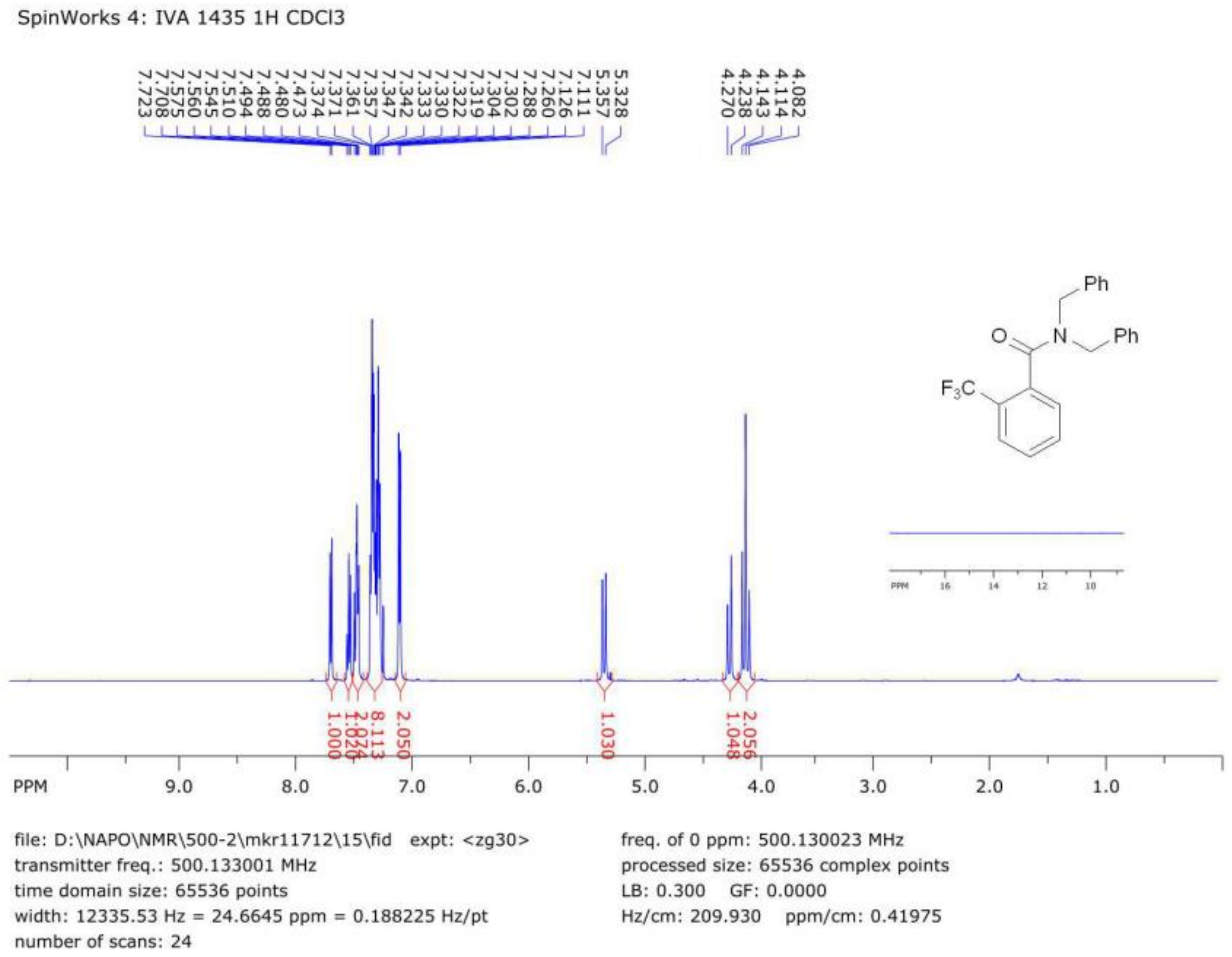

Compound 5ad (NMR in $CDCl_3$)

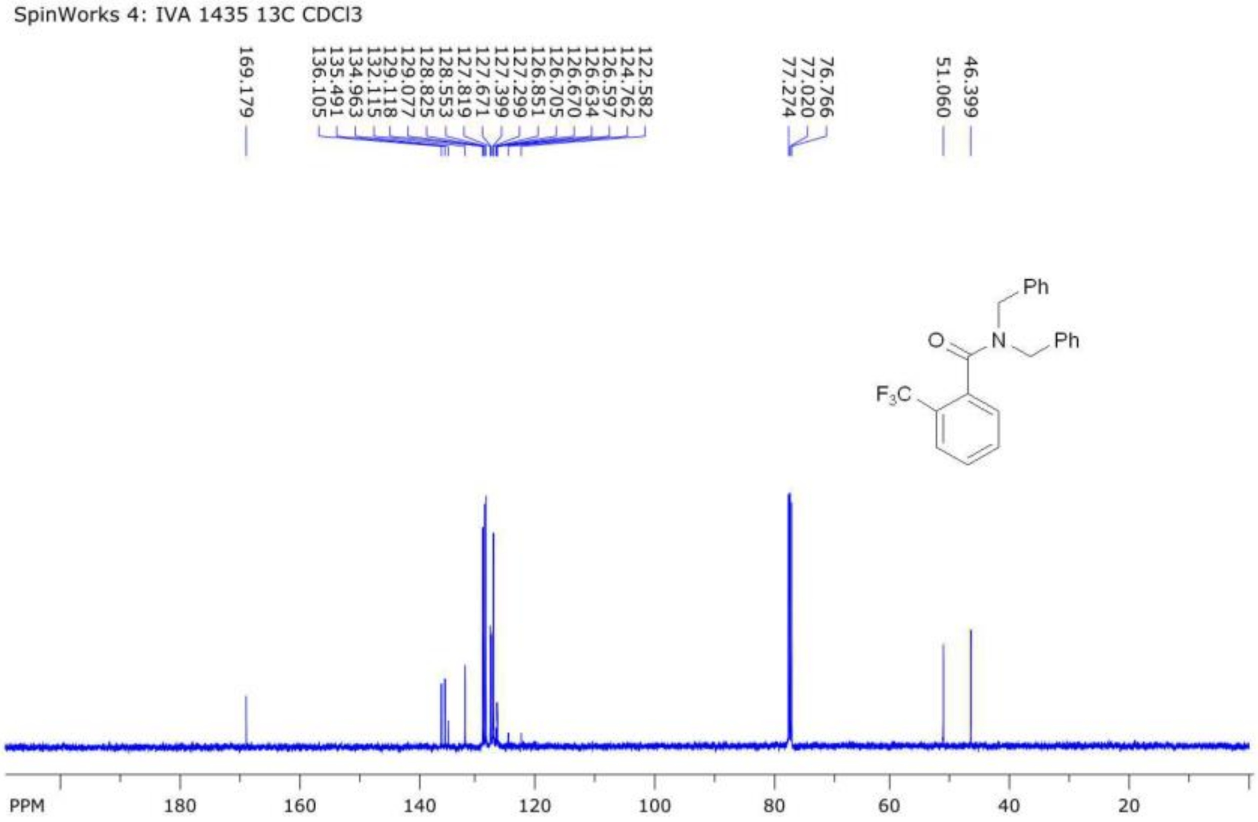

file: D:\NAPO\NMR\500-2\mkr11712\16\fid expt: <zgpg30>
transmitter freq.: 125.772879 MHz
time domain size: 65536 points
width: 36057.69 Hz = 286.6889 ppm = 0.550197 Hz/pt
number of scans: 256

freq. of 0 ppm: 125.757797 MHz
processed size: 32768 complex points
LB: 2.000 GF: 0.0000
Hz/cm: 1060.228 ppm/cm: 8.42971

Compound 5ae (NMR in $CDCl_3$)

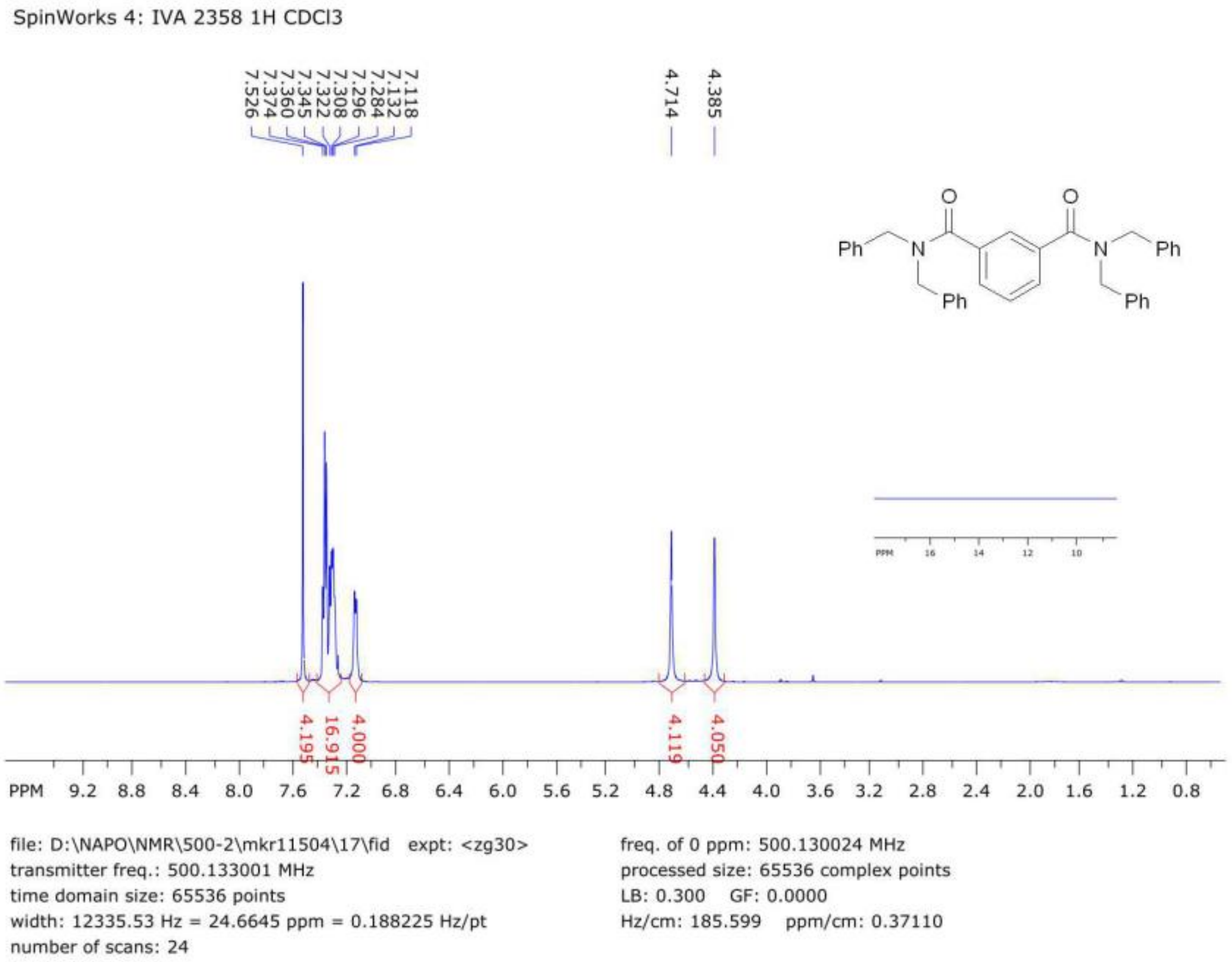

Compound 5ae (NMR in $CDCl_3$)

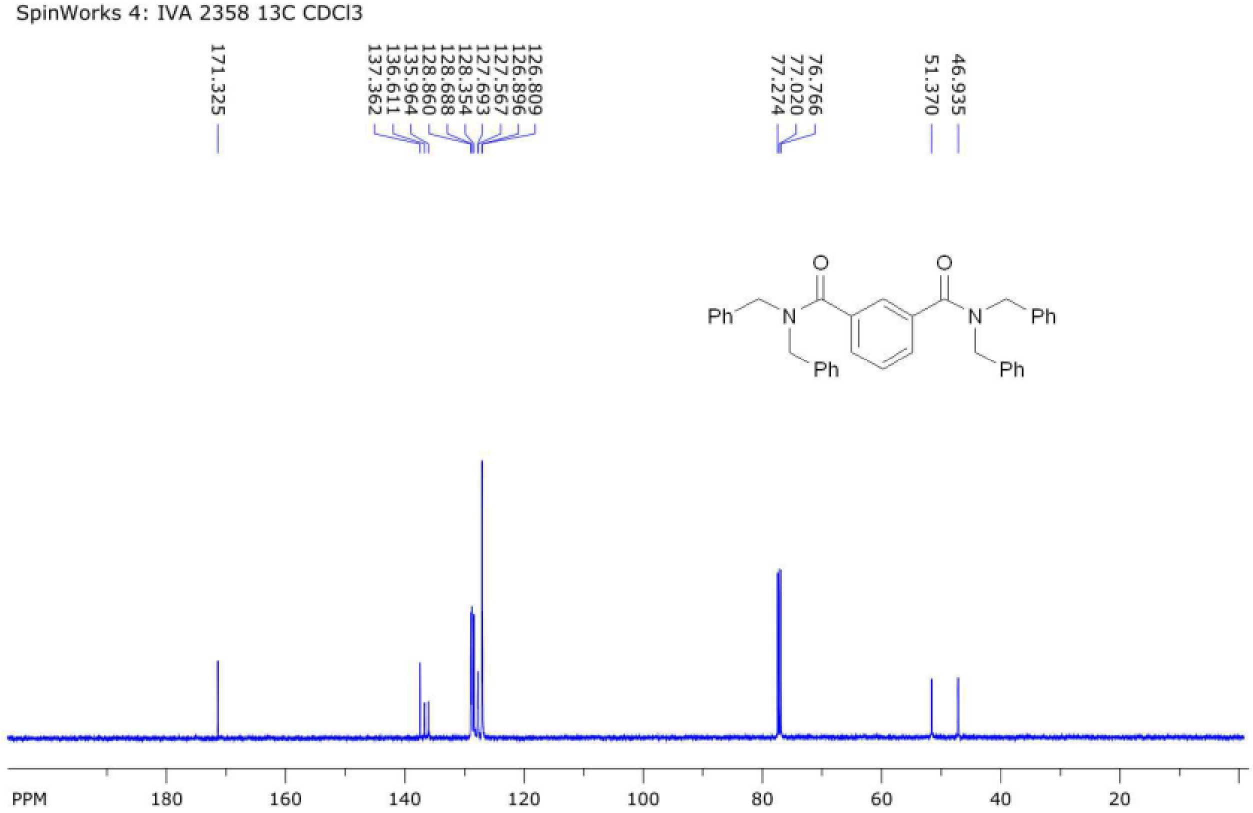

file: D:\NAPO\NMR\500-2\mkr11504\18\fid expt: <zgpg30>
transmitter freq.: 125.772879 MHz
time domain size: 65536 points
width: 36057.69 Hz = 286.6889 ppm = 0.550197 Hz/pt
number of scans: 512

freq. of 0 ppm: 125.757803 MHz
processed size: 32768 complex points
LB: 2.000 GF: 0.0000
Hz/cm: 1045.342 ppm/cm: 8.31135
